# AAV capsid prioritization in normal and steatotic human livers maintained by machine perfusion

**DOI:** 10.1038/s41587-024-02523-6

**Published:** 2025-01-29

**Authors:** Jae-Jun Kim, Simone N. T. Kurial, Pervinder K. Choksi, Miguel Nunez, Tyler Lunow-Luke, Jan Bartel, Julia Driscoll, Chris L. Her, Simaron Dhillon, William Yue, Abhishek Murti, Tin Mao, Julian N. Ramos, Amita Tiyaboonchai, Markus Grompe, Aras N. Mattis, Shareef M. Syed, Bruce M. Wang, Jacquelyn J. Maher, Garrett R. Roll, Holger Willenbring

**Affiliations:** 1 Department of Surgery, University of California, San Francisco, San Francisco, CA USA; 2 Eli and Edythe Broad Center of Regeneration Medicine and Stem Cell Research, University of California, San Francisco, San Francisco, CA USA; 3 Biomedical Sciences Graduate Program, University of California, San Francisco, San Francisco, CA USA; 4 Tetrad Graduate Program, University of California, San Francisco, San Francisco, CA USA; 5 Department of Medicine, University of California, San Francisco, San Francisco, CA USA; 6 Liver Center, University of California, San Francisco, San Francisco, CA USA; 7 Ambys Medicines, South San Francisco, CA USA; 8 Oregon Stem Cell Center, Oregon Health & Science University, Portland, OR USA; 9 Department of Pediatrics, Oregon Health & Science University, Portland, OR USA; 10 Department of Molecular and Medical Genetics, Oregon Health & Science University, Portland, OR USA; 11 Department of Pathology, University of California, San Francisco, San Francisco, CA USA; 12 Present Address: School of Medicine, University of California, Irvine, Irvine, CA USA; 13 Present Address: Pliant Therapeutics, South San Francisco, CA USA; 14 Present Address: Stone Research Foundation, San Francisco, CA USA; 15 Present Address: Genentech, South San Francisco, CA USA; 16 Present Address: Adverum Biotechnologies, Redwood City, CA USA

**Keywords:** Gene therapy, Liver diseases

## Abstract

Therapeutic efficacy and safety of adeno-associated virus (AAV) liver gene therapy depend on capsid choice. To predict AAV capsid performance under near-clinical conditions, we established side-by-side comparison at single-cell resolution in human livers maintained by normothermic machine perfusion. AAV-LK03 transduced hepatocytes much more efficiently and specifically than AAV5, AAV8 and AAV6, which are most commonly used clinically, and AAV-NP59, which is better at transducing human hepatocytes engrafted in immune-deficient mice. AAV-LK03 preferentially transduced periportal hepatocytes in normal liver, whereas AAV5 targeted pericentral hepatocytes in steatotic liver. AAV5 and AAV8 transduced liver sinusoidal endothelial cells as efficiently as hepatocytes. AAV capsid and steatosis influenced vector episome formation, which determines gene therapy durability, with AAV5 delaying concatemerization. Our findings inform capsid choice in clinical AAV liver gene therapy, including consideration of disease-relevant hepatocyte zonation and effects of steatosis, and facilitate the development of AAV capsids that transduce hepatocytes or other therapeutically relevant cell types in the human liver with maximum efficiency and specificity.

## Abstract (web summary)

The suitability of different AAV capsids for gene therapy is assessed in human livers.

## Main

Adeno-associated virus (AAV) vectors proved to be effective and safe for hepatocyte-targeted reversal of factor IX and VIII deficiency in hemophilia B and A, respectively, with the AAV5 capsid being the first to receive Food and Drug Administration (FDA) approval^1,2^. However, these achievements of therapeutic efficacy cannot be readily extended to other liver diseases. Whereas hemophilias can be effectively treated by restoring coagulation factors in the blood to 5% of normal, most other liver diseases such as hyperammonemias require close to 40% of hepatocytes to be transduced^3–6^. Because the AAV vector doses required for effective gene therapy of hemophilia A and B are already high^3^, dose escalation is not a safe option, a painful lesson learned from four fatal cases of liver failure in individuals with X-linked myotubular myopathy who received an AAV8 vector^7^. Thus, broadening the reach of AAV liver gene therapy requires using capsids that maximize efficiency of hepatocyte transduction at a safe vector dose.

There is no shortage of naturally occurring or engineered AAV capsids with potential for human liver gene therapy^8^, but it is difficult to predict how these capsids perform in the human liver. Rodent and nonhuman primate (NHP) studies have overestimated therapeutic efficacy, partly because of species-specific differences in hepatocyte tropism of AAV capsids^9^. Immune-deficient mice repopulated with human hepatocytes address this limitation, but comparisons of AAV capsids have produced contradictory results^10–13^, probably because of differences in donor characteristics and degree of humanization, and largely undefined interactions of engrafted human hepatocytes with mouse liver cells. Prioritizing AAV capsids based on comparison of clinical trial data is complicated by differences in vector dose, production method and expression cassette, exemplified by each hemophilia gene therapy trial using a different promoter and transgene sequence^3^. In addition, limited availability of liver biopsies from individuals treated with AAV vectors impedes correlating therapeutic effects with capsid-specific efficiency of hepatocyte transduction^8^.

Better methods for predicting AAV capsid performance in the human liver are needed to realize the full potential of human liver gene therapy. For hemophilias and other rare genetic liver diseases such as Crigler–Najjar syndrome^14^ that benefit from few corrected hepatocytes, knowing which of the capsids used in clinical trials (most commonly AAV8, AAV5, AAV6 and AAV-LK03 (ref. ^1^)) has the most favorable human hepatocyte transduction profile would help achieve therapeutic efficacy at the smallest and safest vector dose. Along these lines, many liver diseases are impacted by zonation, the functional specification of hepatocytes along the portal vein-to-central vein axis^15^. Species-specific differences exist in zonation of hepatocyte transduction by the AAV8 capsid, with pericentral tropism in mice and periportal tropism in NHPs^16^. Which of these findings applies to humans and whether other AAV capsids also target specific hepatocyte zones is unknown. Illustrating the importance of these questions, ongoing clinical trials use the AAV8 capsid for gene therapy of pericentrally zonated Crigler–Najjar syndrome^17^ and periportally zonated ornithine transcarbamylase deficiency^18^ and phenylketonuria^19^. For other more prevalent liver diseases, new AAV capsids could be developed that transduce human hepatocytes with maximum efficiency and specificity, thereby limiting off-target effects. Other therapeutically relevant liver cell types could also be targeted with low risk of clinical failure.

Addressing this need is made possible by recent advances in human liver preservation or conditioning for transplantation based on ex vivo normothermic machine perfusion (NMP)^20–22^. By circulating oxygenated packed red blood cells (PRBCs), albumin, hormones and nutrients with anatomic fidelity, NMP can maintain human livers in a physiological state, as evidenced by viability and normal metabolic and synthetic function. A recent study supports the feasibility of using human liver NMP for AAV capsid screening but falls short of realizing its potential for informing clinical practice because of limitations in experimental design and implementation, including use of a single diseased split liver with active inflammation, insufficient exclusion of AAV capsid-neutralizing antibodies (NAbs) in the perfusate and analysis of bulk tissue, not single cells^23^. To reliably predict clinical performance, we emphasized bias-free analysis of AAV capsid tropism by using NMP to maintain healthy intact livers under physiological conditions and by excluding confounding factors such as NAbs. To capture AAV capsid-specific efficiency and specificity of transduction, we analyzed all cell types in the human liver individually.

To further inform AAV capsid choice or participant selection in the clinical setting, we investigated whether AAV capsid tropism is altered by hepatic steatosis, which affects 38% of the global population^24^. Studies in mouse models of steatotic liver disease reported decreased^25^ and unaltered^26^ hepatocyte transduction efficiency for the AAV8 and AAV9 capsids, respectively, but clarifying human studies are lacking. Knowing the effects of comorbidities and tailoring AAV vector transduction to disease-specific needs, such as targeting of the affected hepatocyte zone or other liver cell types, are needed to maximize the therapeutic benefit for individual patients.

## Results

### Setup of AAV capsid testing in human liver

**Extended Data Fig. 1:**
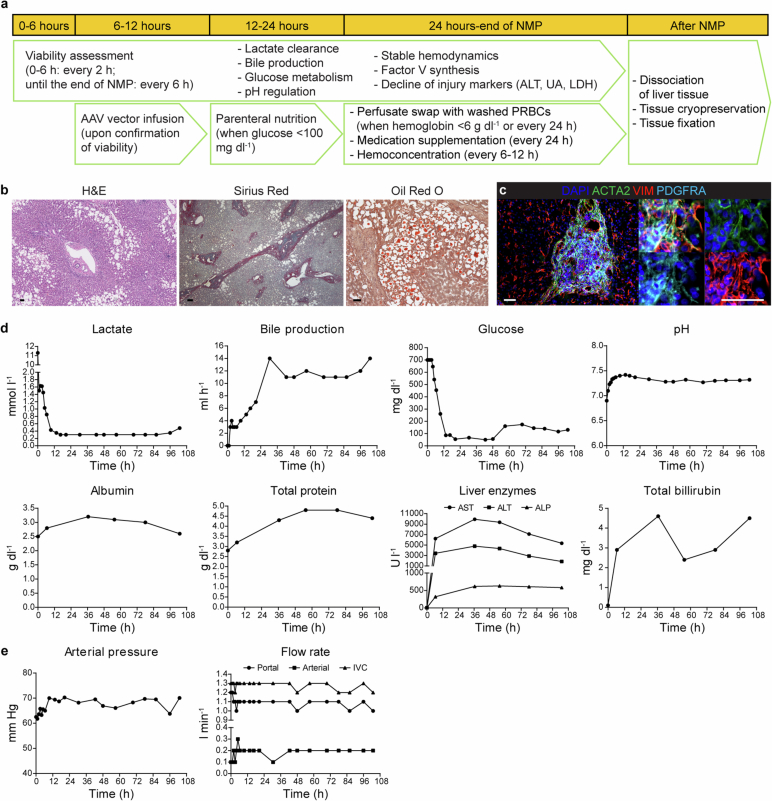
Long-term NMP of a steatotic and fibrotic human liver. **a**, Flowchart illustrating the workflow for long-term NMP and analysis of human livers. **b**, Hematoxylin and eosin (H&E), collagen (Sirius Red) and lipid (Oil Red O) stainings of liver tissue samples after NMP; scale bars, 100 µm. **c**, Immunofluorescence for ACTA2, VIM and PDGFRA in liver tissue samples after NMP; scale bars, 50 µm. **d**, Parameters of viability and function measured during NMP. **e**, Hemodynamic parameters measured during NMP. ALP, alkaline phosphatase; ALT, alanine aminotransferase; AST, aspartate aminotransferase; IVC, inferior vena cava; LDH, lactate dehydrogenase; UA, uric acid.

We first investigated the possibility of long-term NMP with the commercially available OrganOx metra system^20^ (beyond the 24 h used clinically) using a steatotic and fibrotic human liver (steatotic liver 1 (SL 1); Extended Data Fig. 1a–c and Supplementary Table 1). We replaced 20% of the perfusate with PRBCs every 24 h or when the hemoglobin dropped to 6 g dl^–1^. We evaluated the perfused liver according to the same criteria used clinically for selecting transplantable livers by short-term NMP^21^. The liver was viable and functional for 103 h, as evidenced by lactate clearance (<2.5 mmol l^–1^), glucose metabolism (responsiveness to insulin) and pH (7.3–7.4), all measured in the perfusate, and bile production (>4 ml h^–1^; Extended Data Fig. 1d). In addition, the liver remained responsive to continuous infusion of a vasodilator by maintaining a stable hemodynamic profile (Extended Data Fig. 1e).

**Extended Data Fig. 2:**
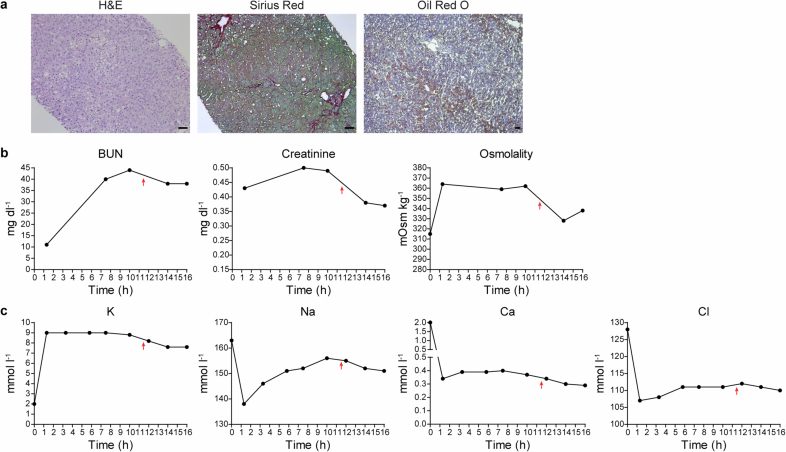
Hemoconcentration mimicking renal function. **a**, Stainings of liver tissue samples before (H&E, Sirius Red) and after (Oil Red O) NMP; scale bars, 50 µm. **b**,**c**, Levels of waste metabolites (**b**) and electrolytes (**c**) measured in perfusate during NMP. Red arrows indicate start of hemoconcentration at 11.5 h of NMP.

To optimize long-term NMP, we added basic renal function by incorporating a hemoconcentrator into the NMP circuit, which we tested in another steatotic liver (SL 2; Extended Data Figs. 1a and 2a). After 3 h of hemoconcentration, levels of blood urea nitrogen, creatinine and osmolality in the perfusate were reduced by about 20%, reflecting removal of waste metabolites (Extended Data Fig. 2b). We balanced electrolyte levels by replacing the hemofiltrate with isotonic dialysate (Extended Data Fig. 2c).

**Extended Data Fig. 3:**
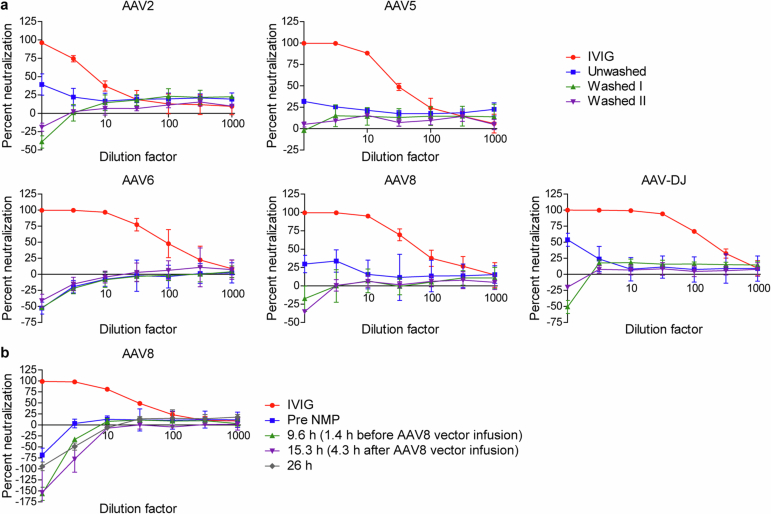
Removal of neutralizing antibodies against AAV capsids by PRBC washing. **a**, Measurement of NAbs against AAV capsids by in vitro neutralization assay in plasma from unwashed or washed PRBCs (washing protocols I and II described in Supplementary Methods) with AAV vectors. **b**, Measurement of NAbs against the AAV8 capsid in plasma from NL 1 perfusate during NMP. Human intravenous immunoglobulin (IVIG) was used as a positive control. Values are presented as mean ± s.d. (*n* = 3, technical replicates).

We also used perfusion of SL 2 to eliminate biases from our approach to AAV capsid evaluation. We removed residual plasma, which can include NAbs to AAV capsids^27,28^, by centrifugation-mediated washing of PRBCs^29^, after which NAbs to the AAV2, AAV5, AAV6, AAV8 and AAV-DJ capsids became nearly undetectable as assessed by luciferase-based neutralization assay (Extended Data Fig. 3a). We also excluded loss of AAV vectors by attachment to the surface of the silicone tubing in the NMP circuit^30^ (Supplementary Fig. 1).

Finally, we minimized the time required to detect functional transduction, that is, mRNA and protein expression by AAV vectors. We constructed a self-complementary AAV (scAAV) vector that expresses a fluorescent protein-encoding transgene from the cytomegalovirus (CMV) promoter. scAAV vectors express transgenes more rapidly than single-stranded AAV vectors because they bypass the need for second-strand DNA synthesis after uncoating of the viral particle in the nucleus^31^. The CMV promoter is ubiquitously active, which allows for comprehensive characterization of AAV capsid tropism^32^. Moreover, the CMV promoter provides rapid-onset and high-level transgene expression—it is subject to silencing in hepatocytes but only after 7 days^33^. To identify the earliest time point when functional transduction could be reliably detected, we intravenously injected scAAV vectors produced with AAV8, AAV5, AAV-LK03 and AAV6 capsids into mice. Expression of vector-derived mRNA and fluorescent protein increased moderately between 48 h and 7 days after injection, reflecting ongoing functional transduction, but the relative transduction efficiencies of the four AAV vectors were essentially the same between the two time points (Supplementary Fig. 2a,b). These results led us to use the scAAV-CMV vector for all subsequent experiments (Supplementary Table 2).

These results establish NMP as an experimental system for testing AAV capsids under physiological conditions in human livers and limit the critical perfusion time after AAV vector administration to 48 h.

### Transduction profile of the AAV8 capsid in normal human liver

**Fig. 1:**
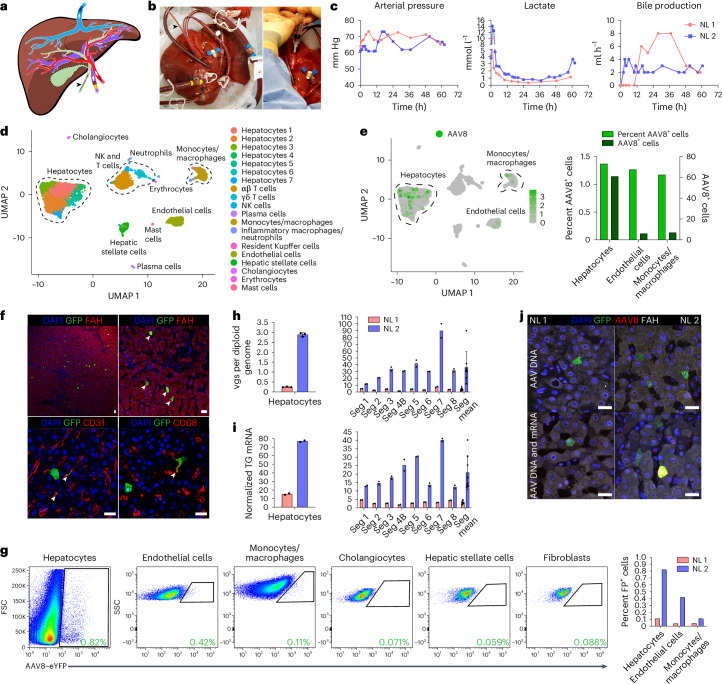
Efficiency and specificity of hepatocyte transduction by the AAV8 capsid in normal human liver. **a**, Cartoon showing cannulation of human liver and direction of flow in blood vessels and the bile duct (gall bladder removed). **b**, Images of cannulated and perfused NL 1 (left) and AAV8–eYFP vector infusion into the portal vein (right). Cannula colors are the same in **a** and **b**, where yellow indicates the portal vein, red indicates the hepatic artery, blue indicates the inferior vena cava, and the black arrowhead indicates the bile duct. **c**, Viability and function measured during NMP of NL 1 and NL 2. Of note, in NL 2, infusion of 1 U of PRBCs at 60 h precipitated an increase in lactate. In NL 1, bile production was not recorded between 0 and 11 h due to a mispositioned bile duct cannula. **d**, Uniform manifold approximation and projection (UMAP) of 6,959 liver cells analyzed by scRNA-seq and clustered according to cell identity; NK, natural killer. **e**, Left, distribution of AAV8^+^ cells in scRNA-seq. Right, percentage and absolute number of AAV8^+^ cells in each cell population. **f**, Immunofluorescence for GFP (AAV8), FAH (hepatocytes), CD31 (endothelial cells) and CD68 (monocytes/macrophages) in tissue samples from NL 2 after NMP. White arrowheads indicate cells positive for both GFP and the respective cell-type-specific marker; scale bars, 25 µm. **g**, Flow cytometry (left) with quantification (right) of AAV8-transduced cells released from NL 2 after 62 h of NMP; FP, fluorescent protein. **h**,**i**, Quantification of AAV vgs per diploid genome (**h**) and transgene (TG) mRNA expression (**i**) in isolated hepatocytes and tissue samples from NL 1 and NL 2. NL 1 received 5.7 × 10^12^ vgs and NL 2 received 1.6 × 10^13^ vgs. Values are presented as mean ± s.d. (*n* = 2 except *n* = 3 hepatocytes in **h**; technical replicates); Seg, segment; Seg mean, mean expression values of all eight segments. **j**, ISH of AAV vector DNA using an eGFP sense probe (top) and both AAV vector DNA and mRNA using an eGFP antisense probe (bottom) combined with immunofluorescence for GFP in tissue samples from NL 1 and NL 2; scale bars, 25 µm.

**Extended Data Fig. 4:**
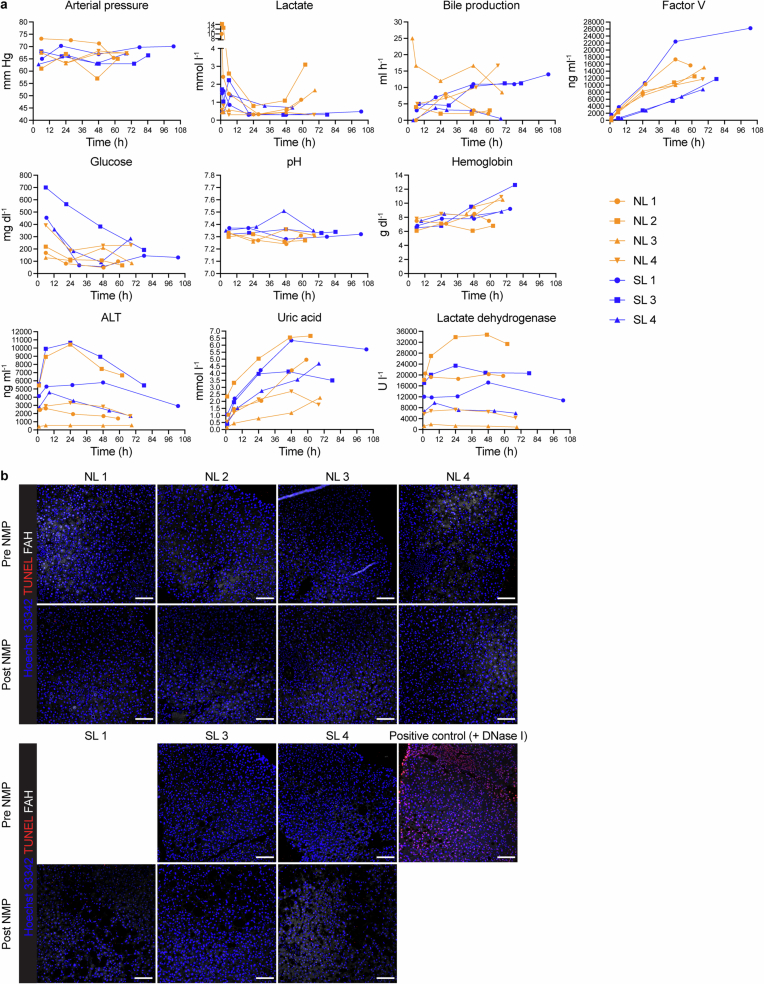
Long-term NMP of normal and steatotic human livers. **a**, Parameters of viability and function measured during NMP of normal and steatotic livers. **b**, TUNEL assay on liver tissue samples before and after NMP. Of note, pre-NMP biopsy was not performed for SL 1; scale bars, 100 µm.

**Extended Data Fig. 5:**
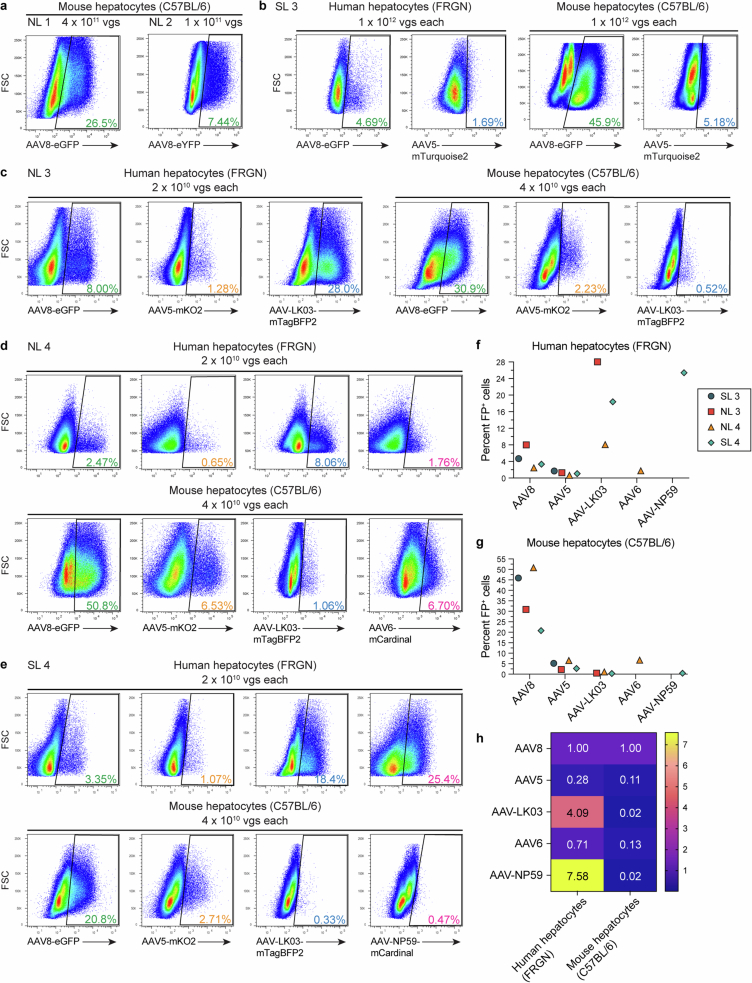
AAV testing in wild-type and humanized mouse livers. **a**, Flow cytometry of hepatocytes released from livers of wild-type mice 2 days after intravenous injection of AAV vector preparations that were used in NL 1 (left) and NL 2 (right). **b**–**e**, Flow cytometry of human or mouse hepatocytes released from livers of FRGN or wild-type mice 2 days after intravenous injection of AAV vector preparations that were used in SL 3 (**b**), NL 3 (**c**), NL 4 (**d**) or SL 4 (**e**). **f**,**g**, Quantification of transduced human (**f**) and mouse (**g**) hepatocytes by flow cytometry. **h**, Heatmap showing relative levels of AAV fluorescent protein expression in human (left) and mouse (right) hepatocytes normalized to the levels of AAV8. For each AAV capsid, the levels were averaged across different mice.

Using our optimized approach, we investigated the efficiency and specificity of hepatocyte transduction of a vector produced with the AAV8 capsid, which is most commonly used in clinical trials of liver gene therapy^1^, in two human livers that were histologically normal according to biopsies taken before NMP (Supplementary Fig. 3 and Supplementary Table 1). Perfusate obtained before vector infusion contained a negligible amount of NAbs to the AAV8 capsid, confirming the efficacy of PRBC washing (Extended Data Fig. 3b). After achieving an optimal hemodynamic and metabolic profile at 12 h of perfusion, we infused 5.7 × 10^12^ vector genomes (vgs) of AAV8–enhanced green fluorescent protein (AAV8–eGFP) into normal liver 1 (NL 1) and 1.6 × 10^13^ vgs of AAV8–enhanced yellow fluorescent protein (AAV8–eYFP) into NL 2 through the portal vein (Fig. 1a–c, Extended Data Fig. 4a and Supplementary Table 2). These doses reflect the range given in clinical trials of liver gene therapy with AAV8 vectors^3^ but are calculated based on liver weight instead of body weight because biodistribution is not a factor in our system (Supplementary Table 1). We ascertained the functionality of these AAV vectors after intravenous injection into mice (Extended Data Fig. 5a). Declining vector DNA levels in the perfusate indicated uptake into the liver (Supplementary Fig. 4a–c). We ended NMP when the lactate level began increasing to ascertain a physiological state (48 h after AAV infusion for NL 1 and 50 h for NL 2; Fig. 1c and Extended Data Fig. 4a).

**Extended Data Fig. 6:**
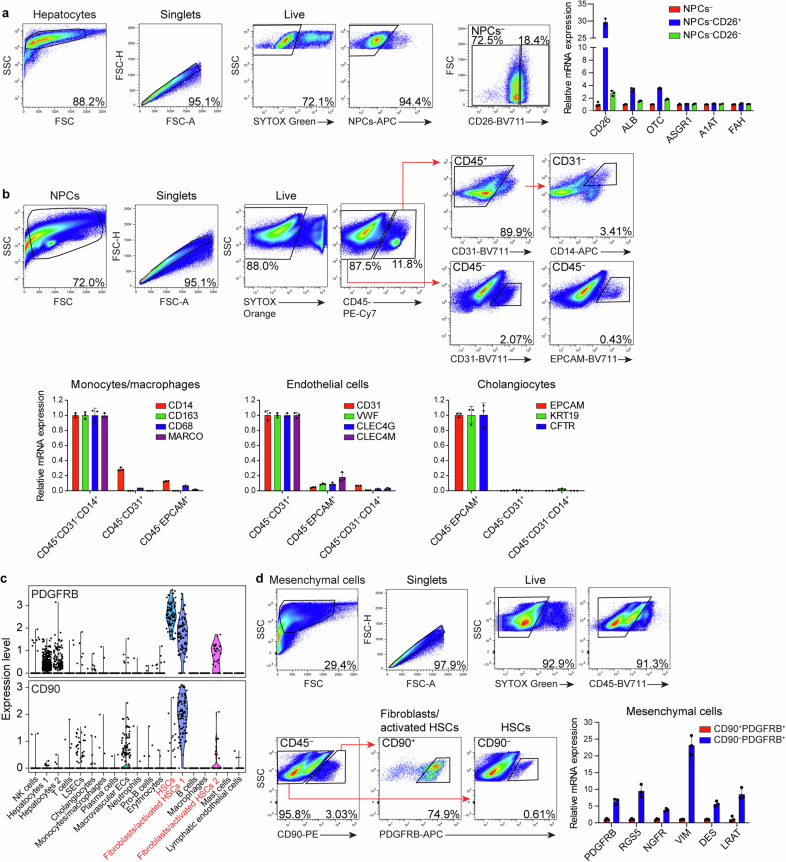
Flow cytometry/FACS of the principal liver cell populations in human liver. **a**, FACS of CD26^+^ hepatocytes (left) released from human liver tissue followed by analysis of cell type-specific markers by qRT-PCR (right). Nonparenchymal cells (NPCs) were excluded by gating on the CD45^–^CD31^–^EPCAM^–^PDGFRB^–^ population. **b**, FACS of NPCs (monocytes/macrophages, endothelial cells and cholangiocytes) from human liver tissue (top) followed by analysis of cell-type-specific markers by qRT-PCR (bottom). **c**, Differential PDGFRB and CD90 expression in hepatic stellate cells (HSCs) and fibroblasts/activated HSCs released from SL 1 and analyzed by scRNA-seq. **d**, FACS of mesenchymal cells (left) followed by analysis of cell-type-specific markers by qRT-PCR (right). Bar graphs represent mean ± s.d. (*n* = 3, technical replicates). Source data

After NMP ended, we enzymatically released hepatocytes and nonparenchymal cells (NPCs), including endothelial cells, monocytes/macrophages, cholangiocytes and mesenchymal cells (hepatic stellate cells and fibroblasts), and analyzed them by single-cell RNA sequencing (scRNA-seq). Analysis of 6,959 cells from NL 2 showed 6 principal and 14 specific cell types (Fig. 1d and Supplementary Fig. 5a–e), with hepatocytes being most efficiently transduced by the AAV8 vector, closely followed by endothelial cells and monocytes/macrophages (Fig. 1e). Immunostaining of fluorescent protein-expressing cells in tissue sections and flow cytometry with optimized antibody panels (Extended Data Fig. 6a–d) confirmed the AAV8 capsid tropism identified by scRNA-seq independent of vector dose (Fig. 1f,g). Quantitative digital droplet PCR (ddPCR) showed dose-dependent differences in vector DNA and transgene mRNA in isolated hepatocytes and tissue samples of the eight liver segments from the two livers, with tight correlation between the two parameters (Fig. 1h,i). In situ hybridization (ISH) with sense or antisense probes targeting vector DNA or both vector DNA and transgene mRNA confirmed this result (Fig. 1j).

These results show that the tropism of the AAV8 capsid in the normal human liver includes endothelial cells and monocytes/macrophages in addition to hepatocytes. These results also establish that the AAV8 capsid affords efficient functional transduction in the human liver as evidenced by corresponding levels of physical vector cell entry and transgene expression, with scRNA-seq being equally as informative as flow cytometry.

### Comparison of AAV capsids in normal and steatotic human livers

We compared the transduction profile of the AAV8 capsid with that of the AAV5, AAV-LK03 and AAV6 capsids, which are most commonly used in clinical trials of liver gene therapy^1^, and the AAV-NP59 capsid, which, based on studies in immune-deficient mice, has the strongest tropism for human hepatocytes^34^. To facilitate side-by-side comparison, for each capsid, we introduced a unique fluorescent protein-encoding transgene and expressed barcode into the AAV vector, allowing for distinction by flow cytometry, microscopy and ddPCR (Supplementary Fig. 6a–d and Supplementary Table 2). We ascertained that scRNA-seq reliably detects transgenes and barcodes (see Methods) and excluded that co-injection of AAV vectors produced with the different capsids alters the transduction efficiency of any individual capsid in mice (Supplementary Fig. 6e,f).

Before testing in human liver, we intravenously co-injected the AAV vectors at the same dose into wild-type mice and FRGN mice repopulated with human hepatocytes. Two days later, we enzymatically released hepatocytes and analyzed fluorescent protein expression by flow cytometry. We found a capsid-specific transduction efficiency of AAV8 > AAV6 > AAV5 > AAV-LK03 > AAV-NP59 in mouse hepatocytes and AAV-NP59 > AAV-LK03 > AAV8 > AAV6 > AAV5 in human hepatocytes (Extended Data Fig. 5b–h), which is in agreement with reports of species-specific hepatocyte tropism of these capsids^10–13,34^.

Because the anticoagulant heparin used for NMP in the clinical setting can reduce the transduction efficiency of the AAV-LK03, AAV6 and AAV-NP59 capsids^35,36^, we substituted low-molecular-weight heparin after screening alternative anticoagulants in mice (Supplementary Fig. 7a–c).

**Fig. 2:**
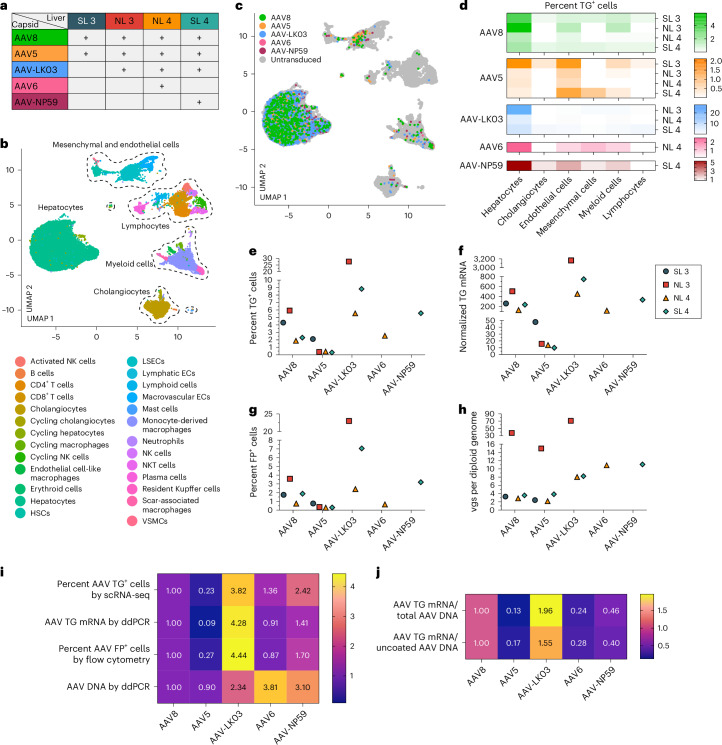
Comparison of efficiency and specificity of hepatocyte transduction by the AAV8, AAV5, AAV-LK03, AAV6 and AAV-NP59 capsids in normal and steatotic human livers. **a**, Assignments of AAV capsids to human livers for side-by-side comparison. SL 3 received 3.4 × 10^13^ vgs, NL 3 received 5.5 × 10^12^ vgs, NL 4 received 6.7 × 10^11^ vgs and SL 4 received 3.0 × 10^12^ vgs of each vector. Of note, the low-producing AAV6 capsid dictated the lower dose in NL 4. **b**, UMAP of 35,807 cells from four livers analyzed by scRNA-seq and clustered according to cell identity; ECs, endothelial cells; LSECs, liver sinusoidal endothelial cells; HSCs, hepatic stellate cells; VSMCs, vascular smooth muscle cells. **c**, UMAP showing cells transduced by AAV capsids. **d**, Heat map showing percent transduction by AAV capsids across cell populations. **e**,**f**, Quantification of AAV transgene mRNA-expressing hepatocytes by scRNA-seq (**e**) and ddPCR (**f**). **g**, Quantification of AAV fluorescent protein-expressing hepatocytes by flow cytometry. **h**, Quantification of AAV vector DNA (vgs) per diploid genome in hepatocytes by ddPCR. **i**, Heat map showing the relative levels of AAV vector mRNA, protein and DNA among five AAV capsids normalized to the levels of AAV8. The levels from four livers were averaged for each capsid. **j**, Heat map showing the relative levels of the ratio between AAV transgene mRNA and total AAV vector DNA from hepatocytes (top row) and AAV transgene mRNA and uncoated nuclear AAV vector DNA from tissue samples (bottom row). Levels were normalized to the levels of AAV8 and averaged from four livers for each capsid.

We co-infused AAV vectors produced with the five capsids into four human livers, two histologically normal (NL 3 and NL 4) and two steatotic (SL 3 and SL 4; Supplementary Fig. 3 and Supplementary Table 1). To contextualize our results, we sequentially introduced additional AAV vectors in combination with AAV8, which served as an internal control, with the AAV8, AAV5, AAV-LK03, AAV6 and AAV-NP59 capsids being tested in four, four, three, one and one livers, respectively (Fig. 2a). We co-infused AAV vectors at the same dose, ranging from 6.7 × 10^11^ vgs to 3.4 × 10^13^ vgs per vector between livers (Supplementary Table 2). Both normal and steatotic livers were viable, as assessed by analysis of liver injury and function in perfusate and cell death in tissue sections, and hemodynamically stable throughout NMP, up to and including the termination time of 60–73 h after AAV vector infusion (Extended Data Fig. 4a,b). We retrieved hepatocytes and NPCs at high yield from all livers, allowing for integrated clustering of 6 principal and 26 specific cell types by scRNA-seq (Fig. 2b and Supplementary Fig. 8a). All five AAV vectors primarily transduced hepatocytes. Among NPCs, endothelial cells and myeloid cells were most frequently transduced, whereas cholangiocytes, mesenchymal cells and lymphocytes showed low levels of transduction (Fig. 2c,d). Notably, AAV-LK03 was most specific for hepatocytes, with minimal transduction occurring in NPCs.

We next quantified capsid-specific transduction at the mRNA, protein and DNA levels in hepatocytes by scRNA-seq, flow cytometry and ddPCR. In hepatocytes, we found a functional transduction (mRNA and protein) efficiency of AAV-LK03 > AAV-NP59 > AAV8 ≥ AAV6 > AAV5 (mRNA range: 27.7% to 0.3%, protein range: 23% to 0.3%) and a physical transduction (DNA) efficiency of AAV6 > AAV-NP59 > AAV-LK03 > AAV8 > AAV5 (range: 70.9 to 2.2 vgs per diploid genome; Fig. 2e–i and Supplementary Fig. 9a–d). The relative transduction efficiency in the eight segments of each liver paralleled the levels in hepatocytes, which we confirmed in tissue sections by ISH of vector DNA and mRNA (Supplementary Figs. 10a–c and 11a–d). Detection of unique capsid barcode sequences alone by scRNA-seq further confirmed the capsid transduction hierarchy (Supplementary Fig. 10d). Notably, the functional transduction efficiency of AAV-NP59 was 55% less than that of AAV-LK03, the opposite of what has been reported in immune-deficient mice engrafted with human hepatocytes, which we independently confirmed^34^ (Extended Data Fig. 5e,f,h).

We also investigated capsid-specific differences in transcription efficiency by quantifying the ratio of AAV transgene mRNA to uncoated AAV vgs within nuclei, that is, DNA available for transcription (Supplementary Fig. 10e–g). Transcription efficiency was highest for AAV-LK03 and lowest for AAV5 (Fig. 2j).

These results show that the AAV-LK03 capsid transduces hepatocytes in human liver much more efficiently and specifically than the AAV-NP59, AAV8, AAV6 and AAV5 capsids. By establishing the superiority of AAV-LK03 for hepatocyte-targeted human liver gene therapy, our results highlight limitations of immune-deficient mice engrafted with human hepatocytes^34^. In addition, these results uncover capsid-specific effects on AAV vector transcription in the human liver, which aligns with reports of an epigenetic role of the AAV capsid^37,38^.

### AAV capsid-specific effects of hepatocyte zonation and steatosis

**Fig. 3:**
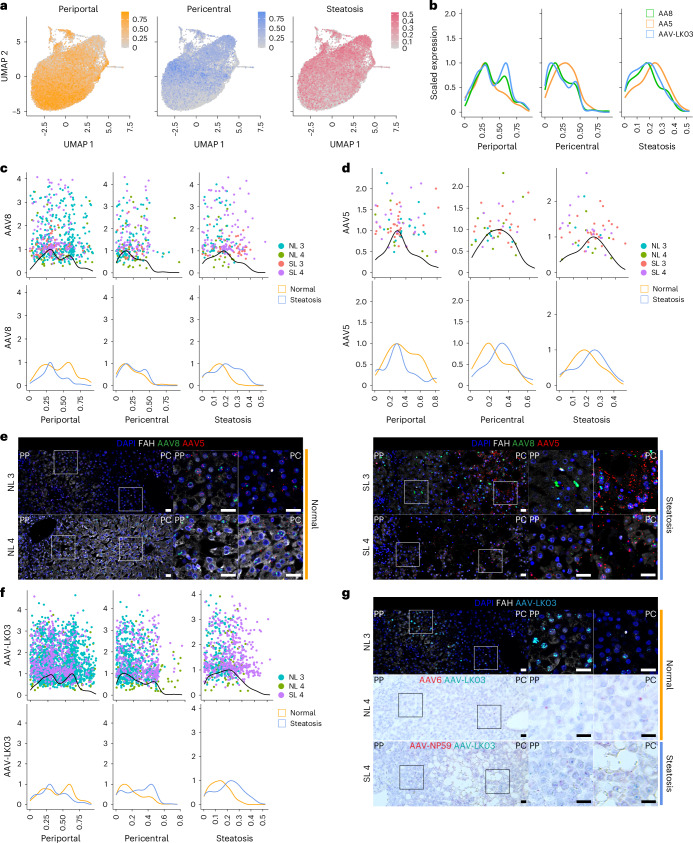
Comparison of zonation of hepatocyte transduction by the AAV8, AAV5, AAV-LK03, AAV6 and AAV-NP59 capsids in normal and steatotic human livers. **a**, Gene module scores for periportal and pericentral zonation and steatosis applied to UMAPs of 22,146 hepatocytes from four livers analyzed by scRNA-seq. **b**, Kernel density estimation histograms of transgene-positive hepatocyte distribution across gene module scores. **c**, Kernel density estimation histograms of hepatocytes transduced by the AAV8 capsid across gene module scores (top) and separated by disease state (bottom). **d**, Kernel density estimation histograms of hepatocytes transduced by the AAV5 capsid across gene module scores (top) and separated by disease state (bottom). **e**, ISH of AAV8 and AAV5 vector DNA with sense probes in tissue samples; scale bars, 25 µm; PP, periportal; PC, pericentral. **f**, Kernel density estimation histograms of hepatocytes transduced by the AAV-LK03 capsid across gene module scores (top) and separated by disease state (bottom). **g**, ISH of AAV-LK03 vector DNA with sense probes in tissue samples. Kernel density estimation histograms for AAV6 and AAV-NP59 capsids are shown in Extended Data Fig. 7f; scale bars, 25 µm.

**Extended Data Fig. 7:**
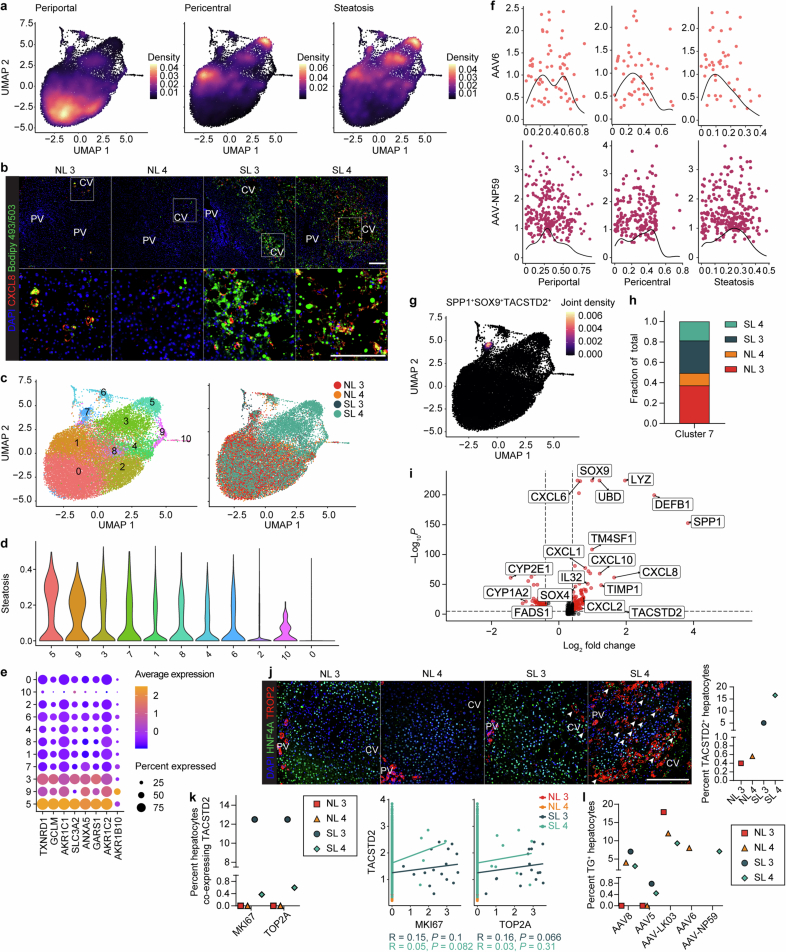
Analysis of disease state and unique hepatocyte subpopulation. **a**, Expression density plots for periportal zonation, pericentral zonation and steatosis gene module scores. **b**, Immunofluorescence for CXCL8 and Bodipy 493/503 labeling in liver tissue samples; scale bars, 200 µm; PV, portal vein; CV, central vein. **c**, UMAPs of 22,146 hepatocytes from four livers analyzed by scRNA-seq and clustered according to gene expression (left) or sample identity (right). **d**, Normalized and log-transformed expression levels of steatosis gene module score in cell clusters from (**c**). **e**, Gene expression profile of differentially expressed genes from clusters 5, 3 and 9. **f**, Kernel density estimation histograms of hepatocytes transduced by the AAV6 capsid or AAV-NP59 capsid across gene module scores. **g**, Expression density plot showing cluster of nonzonated hepatocytes expressing *SPP1*, *SOX9* and *TACSTD2*. **h**, Population distribution of cluster of nonzonated hepatocytes expressing *SPP1*, *SOX9* and *TACSTD2* across four livers. **i**, Volcano plot showing top differentially expressed genes in the cluster of nonzonated hepatocytes expressing *SPP1*, *SOX9* and *TACSTD2*. Inflammatory (*CXCL6*, *CXCL10*, *CXCL8*, *CXCL2*), fibrogenic (*TIMP1*), steatosis (*UBD*, *DEFB*, *IL32*) and cholangiocyte (*SOX4*, *SOX9*, *SPP1*, *TM4SF1*, *TACSTD2*) marker genes are upregulated, while hepatocyte identity genes (*CYP2E1*, *CYP1A2*) and *FADS1* are downregulated. *P* values were calculated using Wilcoxon rank-sum test and adjusted with Bonferroni’s correction. **j**, Immunofluorescence for TROP2 in liver tissue samples (left) and quantification of *TACSTD2*-expressing hepatocytes in scRNA-seq (right); scale bar, 200 µm. White arrowheads indicate hepatocytes positive for both TROP2 and HNF4A. **k**, Co-expression of *TACSTD2* and *MKI67* or *TOP2A* visualized by percent overlap (left) or expression relationship (right) in *TACSTD2*-expressing cells. **l**, Quantification of AAV transgene mRNA-expressing *TACSTD2*-expressing hepatocytes.

To determine whether any of the five AAV capsids preferentially transduces hepatocytes in a specific zone of the human liver and how this tropism may be altered in the setting of steatosis, we analyzed scRNA-seq data from 22,146 hepatocytes from NL 3, NL 4, SL 3 and SL 4. We generated a periportal and pericentral score derived from the expression of zonation markers conserved across all four livers to quantify the zonation enrichment on a per cell basis (Extended Data Table 1), which revealed a consistent pattern of functional zonation irrespective of disease state (Fig. 3a and Extended Data Fig. 7a). Similarly, in assessing the effect of steatosis on these parameters, we adopted a nonbinary approach to cell classification by scoring each cell individually based on expression of steatotic marker genes (Fig. 3a and Extended Data Table 1). This approach revealed a spectrum of steatosis, with normal livers containing some steatotic cells and steatotic livers containing some normal cells, and enabled us to identify CXCL8 as a highly specific and conserved marker of steatotic hepatocytes across normal and steatotic livers (Extended Data Table 1 and Extended Data Fig. 7b).

We also investigated whether the most steatotic hepatocytes exhibit altered transcriptomic signatures. We focused on hepatocytes from SL 4, the most steatotic liver by histologic score, because 42% of these hepatocytes clustered separately from the other livers despite integration (Extended Data Fig. 7c). These clusters were uniquely enriched for expression of genes in the NADPH reduction pathway (*TXNRD1*, *GCLM*, *AKR1C1* and *AKR1C2*), which plays an important role in fatty acid biosynthesis^39^ (Extended Data Fig. 7d,e). Zonation was retained in these cells, consistent with it being altered only in end-stage liver disease^40^ (Fig. 3a and Extended Data Fig. 7a).

These results showed that our steatosis scoring method accurately reflects tissue histology and that our zonation scoring method is applicable to all livers regardless of steatosis severity. We applied these methods to determine the effects of steatosis on AAV capsid transduction at single-cell resolution. We first compared AAV capsid behavior by plotting hepatocytes transduced with the three capsids most commonly used in clinical trials against their respective zonation and steatosis scores (Methods and Fig. 3b). This analysis revealed higher periportal scores for AAV8 and AAV-LK03 than for AAV5, which conversely showed unique pericentral score enrichment. AAV5 transduction was associated with a higher steatosis score, which reflected its high pericentral score^41^ (Fig. 3b).

Next, we analyzed normal and steatotic livers separately to determine the contribution of disease state to zonation of AAV capsid transduction. Our scoring method showed that the weak periportal zonation of AAV8 was specific to normal livers (Fig. 3c), whereas pericentral zonation of AAV5 was specific to steatotic livers (Fig. 3d), which we confirmed in tissue sections by ISH of vector DNA (Fig. 3e) and/or mRNA (Supplementary Fig. 11a–d). We found differential expression of genes associated with steatosis and steatotic liver disease in hepatocytes exclusively transduced by AAV5. Pericentrally zonated *LPCAT2* (ref. ^42^) and *LPCAT1*, genes involved in phosphatidylcholine biosynthesis and lipid droplet formation and remodeling^43^, were enriched, whereas *FADS1*, which protects hepatocytes from lipid accumulation^44^, and *FADS2* were depleted in SL 3; *C3AR1* and *DUSP9*, which regulate lipid accumulation^45,46^, were enriched in SL 4, and pericentrally zonated *SLCO1B3* (ref. ^47^) was enriched in both steatotic livers (Supplementary Data 1). ISH confirmed that AAV5 favors steatotic pericentral hepatocytes by showing vector DNA (Fig. 3e) and/or mRNA (Supplementary Fig. 11a,d) accumulating in lipid-laden cells. There were also steatosis-dependent changes in AAV-LK03 zonation. The strong periportal zonation of AAV-LK03 observed in normal liver was lost in the setting of steatosis, where its pericentral zonation was increased, which we confirmed by ISH of tissue sections (Fig. 3f,g and Supplementary Fig. 11b–d). For AAV6, our zonation model and ISH showed weak periportal zonation in normal liver, whereas AAV-NP59 showed pericentral zonation in steatotic liver (Extended Data Fig. 7f and Supplementary Fig. 11c,d). These tropisms could not be explained by the presence or absence of known AAV entry factors^48^ (Supplementary Fig. 8b,c). A high steatosis score did not negatively impact the transduction efficiency of any of the AAV capsids we tested (Fig. 3c,d,f).

Notably, we identified a population of nonzonated hepatocytes both in normal and steatotic livers marked by the expression of *SOX9*, *SPP1* and *TACSTD2* (Extended Data Fig. 7g–i). The number of *TACSTD2* (TROP2)-expressing hepatocytes increased considerably with steatosis severity, which was not paralleled by an increase in markers of proliferation (Extended Data Fig. 7j,k), suggesting that TROP2 expression in hepatocytes reflects reaction to disease, not expansion of a progenitor cell population as previously suggested^19^. The cells can be transduced by all capsids we tested, with AAV-LK03 being the most efficient, which highlights this hepatocyte population as a potential therapeutic target in steatotic liver disease (Extended Data Fig. 7l).

These results show that the AAV-LK03 capsid preferentially transduces periportal hepatocytes in normal human liver but lacks this zone-specific tropism in the steatotic human liver. AAV8 shows similar trends, but its periportal hepatocyte tropism is much less pronounced in human liver than in NHP liver^16^. Meanwhile, the AAV5 capsid has a strong tropism for pericentral hepatocytes in the steatotic human liver but transduces hepatocytes evenly in the normal human liver^49^.

### AAV capsid-specific and steatosis effects on vector episome formation

**Fig. 4:**
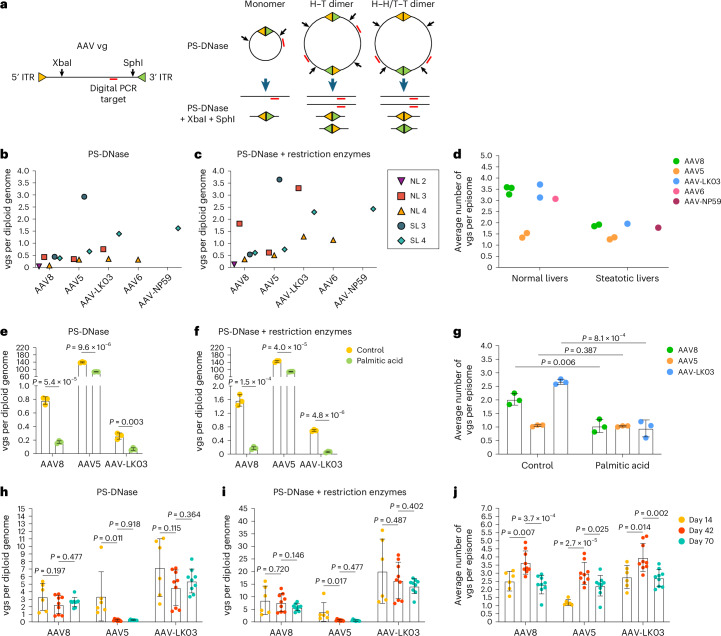
Efficiency of AAV vector episome formation in normal and steatotic human hepatocytes. **a**, Schematics showing predicted AAV vg structures following treatment with Plasmid-Safe DNase (PS-DNase) alone or in combination with restriction enzymes (XbaI and SphI). PS-DNase treatment allows for quantification of circular episomes by digital PCR; additional restriction enzyme treatment allows for quantification of total vector DNA (vgs) in circular episomes; H–T, head to tail; H–H, head to head; T–T, tail to tail. **b**,**c**, Quantification of circular episomes (**b**) and total vgs in circular episomes (**c**) in nuclei from segment three of human liver tissues. BmtI and SphI were used to cut vector DNA from SL 3. **d**, Quantification of the average number of vgs per episome in nuclei from segment three of normal livers (NL 2, NL 3 and NL 4) and steatotic livers (SL 3 and SL 4). The average number was calculated by dividing the number in **c** by the number in **b**. **e**–**g**, Quantification of circular episomes (**e**), total vgs in circular episomes (**f**) and the average number of vgs per episome (**g**) in total DNA from iPS cell-Heps 7 days after AAV vector transduction. Three AAV vectors were cotransduced at multiplicities of infection of 20,000 for the AAV8 capsid, 1,000 for the AAV5 capsid and 30 for the AAV-LK03 capsid. Palmitic acid was added at 200 µM every 2 days for 9 days. Values are presented as mean ± s.d. (*n* = 3, technical replicates). **h**–**j**, Quantification of circular episomes (**h**), total vgs in circular episomes (**i**) and the average number of vgs per episome (**j**) in tissue samples from FRGN mouse livers repopulated with human hepatocytes after co-injection of AAV vectors at the same dose of 4 × 10^10^ vgs. Values are presented as mean ± s.d. (six lobes from two mice at day 14, nine lobes from three mice at day 42 and nine lobes from three mice at day 70; biological replicates). Means were compared using two-tailed unpaired *t*-tests.

We next investigated whether steatosis impacts episome formation of AAV vectors, which is critical for long-term transgene expression^50^. We isolated nuclei from liver tissue from NL 2, NL 3, NL 4, SL 3 and SL 4 to quantify vector episomes using digital PCR (Fig. 4a and Methods)^49^ and confirmed that circular monomers and concatemers form in human livers within 73 h (Fig. 4b,c). We found fewer vgs in episomes isolated from steatotic livers than in episomes isolated from normal livers, independent of which of the five AAV capsids was used to produce the vector, suggesting that circular concatemerization of AAV vectors was compromised in steatotic livers (Fig. 4d).

**Extended Data Fig. 8:**
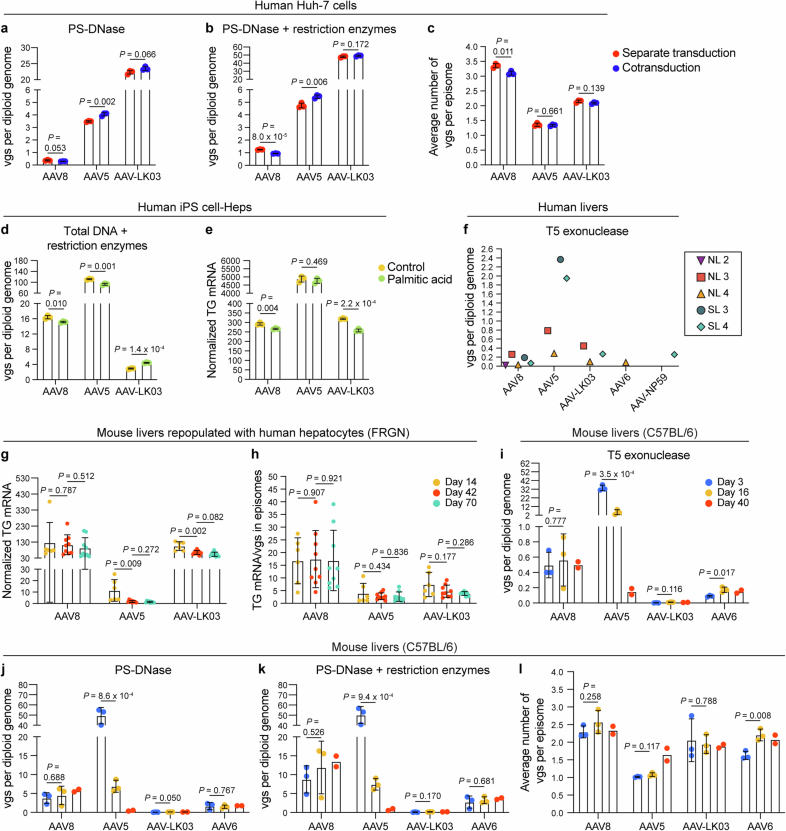
AAV vector DNA analysis in human and mouse livers. **a**–**c**, Quantification of circular episomes (**a**), total AAV vector DNA (vgs) in circular episomes (**b**) and average number of vgs per episome (**c**) in total DNA from Huh-7 cells 3 days after AAV vector transduction. Three AAV vectors were separately or simultaneously transduced at multiplicities of infection of 20,000 for the AAV8 capsid, 1,000 for the AAV5 capsid and 100 for the AAV-LK03 capsid. Values are presented as mean ± s.d. (*n* = 3, technical replicates). Means were compared using two-tailed unpaired *t*-tests. **d**,**e**, Quantification of total vgs (**d**) and transgene mRNA (**e**) in iPS cell-Heps 7 days after AAV vector transduction. Cells were treated with 200 µM palmitic acid every 2 days for 9 days. Values are presented as mean ± s.d. (*n* = 3, technical replicates). Means were compared using two-tailed unpaired *t*-tests. **f**, Quantification of circular episomes in nuclei from segment three of human livers by treatment with T5 exonuclease. **g**,**h**, Quantification of AAV transgene mRNA (**g**) and ratio between AAV transgene mRNA and total vgs in circular episomes (**h**) in tissue samples from mouse livers repopulated with human hepatocytes after co-injection of AAV vectors at the same dose of 4.0 x 10^10^ vgs. Values are presented as mean ± s.d. (six lobes from two mice at day 14, nine lobes from three mice at day 42, nine lobes from three mice at day 70; biological replicates). Means were compared using two-tailed unpaired *t*-tests. **i**–**l**, Quantification of vgs with treatment of T5 exonuclease alone (**i**), PS-DNase alone (**j**), PS-DNase combined with restriction enzymes (**k**) and the average number of vgs per episome (**l**) in total DNA from wild-type mouse livers. Four AAV vectors were co-injected at a dose of 2.0 x 10^10^ vgs each. Values are presented as mean ± s.d. (*n* = 3 mice at day 3, *n* = 3 mice at day 16, *n* = 2 mice at day 40; biological replicates). Means were compared using two-tailed unpaired *t*-tests.

To confirm this result, we analyzed episome formation by vectors produced with the AAV8, AAV5 and AAV-LK03 capsids cotransduced into human induced pluripotent stem cell-derived hepatocytes (iPS cell-Heps) treated with palmitic acid, a saturated fatty acid that causes steatosis^51^. As a prerequisite, we ascertained that cotransduction does not impact quantification of episome formation (Extended Data Fig. 8a–c). We found that palmitic acid treatment for 7 days caused the number of episomes to decline (Fig. 4e,f). Palmitic acid had little effect on physical transduction and transgene expression, indicating that not only episomes but also linear vectors are available for transcription soon after transduction (Extended Data Fig. 8d,e). Confirming our findings in human livers, AAV8 and AAV-LK03 vectors failed to form concatemers in steatotic iPS cell-Heps, whereas the AAV5 vector was unaffected because it predominantly formed circular monomers despite a high number of transduced vgs (Fig. 4g and Extended Data Fig. 8d).

Next, we investigated long-term episome formation by AAV vectors, focusing on the distinctive effect of the AAV5 capsid, leading to predominant formation of circular monomers in human livers (Fig. 4b–d and Extended Data Fig. 8f). We analyzed AAV8, AAV5 and AAV-LK03 episomes in FRGN mice repopulated with human hepatocytes to more than 90% (ref. ^52^). The number of vgs in AAV8 and AAV-LK03 episomes was stable for 70 days, and they rapidly formed large concatemers (Fig. 4h–j). This finding confirms findings in livers of NHPs where concatemers formed as early as 3 days and lasted for 90 days after intravenous injection of an AAV8 vector^53^. By contrast, the number of vgs in AAV5 episomes declined over time, probably because of degradation, with concatemers being detectable only by day 42. The number of vgs in episomes dictated the transcriptional output at all time points for all capsids, with linear vgs probably contributing to transcription initially (Extended Data Fig. 8g,h). AAV5 episomes had the lowest transcription efficiency across all time points, contradicting previous findings in mouse liver that monomers are more efficiently transcribed than concatemers^54^. Episome kinetics of these capsids were similar in wild-type mice, with AAV5 showing rapid circular monomer formation followed by a steep decline in the number of vgs in episomes and concatemerization occurring only by day 40 (Extended Data Fig. 8i–l).

These results reveal that the AAV capsid influences the kinetics of vector episome formation, probably by affecting the recombination of inverted terminal repeats (ITRs)^55^. The AAV5 capsid causes formation of relatively unstable episomes that concatemerize more slowly than AAV8 and AAV-LK03 episomes, which translates into lower long-term transgene expression. In addition, these results identify steatosis as a capsid-independent factor impairing episome concatemerization and thereby the durability of transgene expression from AAV vectors.

### AAV capsid-specific nonhepatocyte tropism and co-regulated genes

**Fig. 5:**
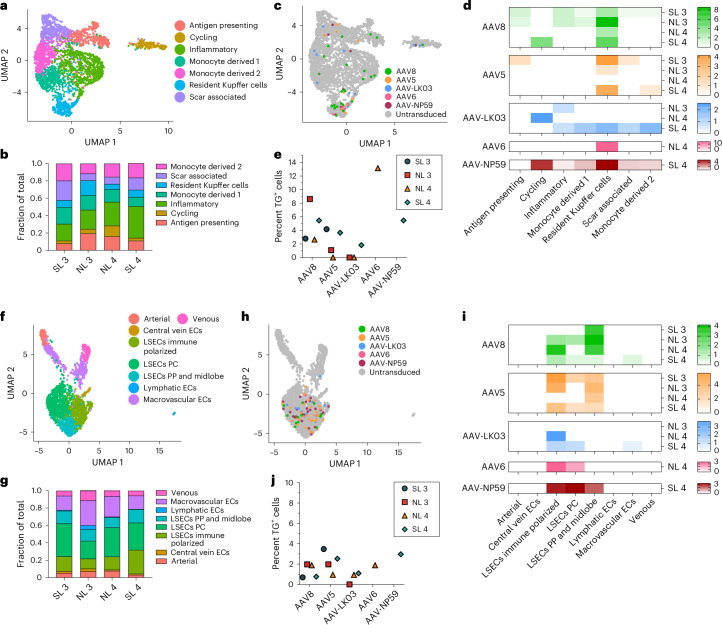
Efficiency of transduction of monocyte/macrophage and endothelial cell subtypes by the AAV8, AAV5, AAV-LK03, AAV6 and AAV-NP59 capsids in normal and steatotic human livers. **a**, UMAP of 2,759 monocytes/macrophages from four livers clustered according to cell subtype. **b**, Population distribution of monocyte/macrophage subtypes. **c**, AAV-transduced cells visualized by UMAP. **d**, Heat map showing percent transduction by AAV capsids of monocyte/macrophage subtypes. **e**, Quantification of AAV transgene mRNA-expressing Kupffer cells by scRNA-seq. **f**, UMAP of 2,091 endothelial cells from four livers clustered according to cell subtype; ECs, endothelial cells; LSECs, liver sinusoidal endothelial cells; PP, periportal; PC, pericentral. **g**, Population distribution of endothelial cell subtypes. **h**, AAV-transduced cells visualized by UMAP. **i**, Heat map showing percent transduction by AAV capsids of endothelial cell subtypes. **j**, Quantification of AAV transgene mRNA-expressing LSECs by scRNA-seq.

**Extended Data Fig. 9:**
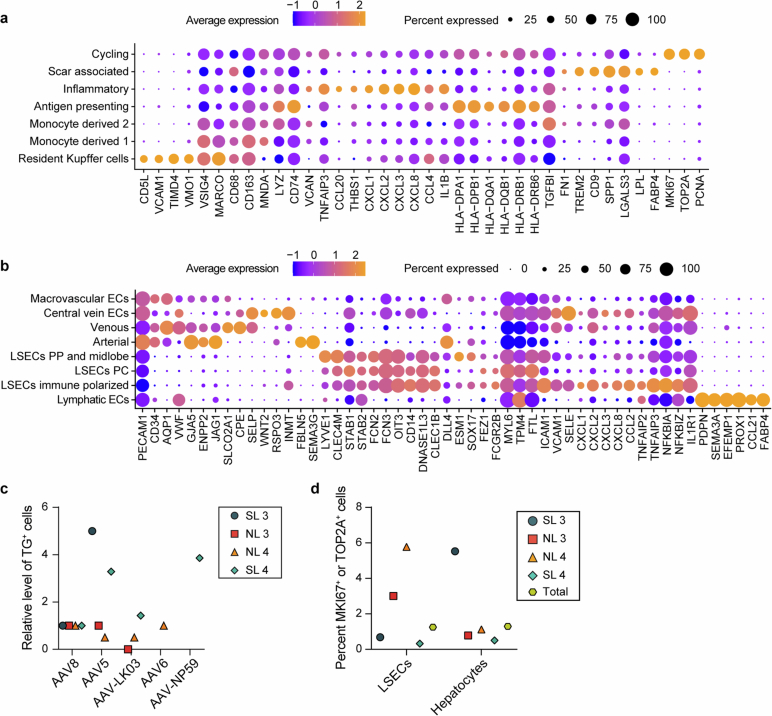
AAV vector transduction in non-hepatocytes. **a**, Cell-type-specific markers used to identify monocyte/macrophage subtypes. **b**, Cell-type-specific markers used to identify endothelial cell subtypes. ECs, endothelial cells; LSECs, liver sinusoidal endothelial cells; PC, pericentral; PP, periportal. **c**, Quantification of AAV transgene mRNA-expressing LSECs normalized to the levels of AAV8. **d**, Percent of LSECs or hepatocytes expressing either *MKI67* or *TOP2A*.

The tropism of AAV capsids for human liver cell types beyond hepatocytes is unknown, which may have contributed to complications or obscured opportunities in the clinical setting. Our integrated dataset from NL 3, NL 4, SL 3 and SL 4, including over 35,800 cells and 26 specific cell types, revealed that AAV vectors consistently transduce not only hepatocytes but also endothelial cells and monocytes/macrophages (Fig. 2c,d). We separately analyzed all endothelial cells and monocytes/macrophages to identify potential heterogeneity in transduction within these populations. From 2,759 monocytes/macrophages, we discerned seven distinct types, including scar-associated macrophages reported in fibrotic livers^56^ that were most prevalent in SL 3 and SL 4 (Fig. 5a,b and Extended Data Fig. 9a). We found a clear tropism for resident Kupffer cells of all capsids except AAV-LK03, with AAV6 and AAV8 being most efficient, transducing up to 13.2% and 8.6% of Kupffer cells, respectively (Fig. 5b–e).

Subclustering of 2,091 endothelial cells distinguished eight subpopulations, of which the five capsids almost exclusively transduced liver sinusoidal endothelial cells (LSECs), with AAV5 and AAV8 being most efficient (Fig. 5f–j and Extended Data Fig. 9b). In steatotic livers, AAV5 transduced LSECs 3.3–5 times better than AAV8 to a maximum of 3.5%, whereas in normal livers, AAV8 was equivalent or better than AAV5 at 2% (Fig. 5j and Extended Data Fig. 9c). This unique tropism probably reflects the increase in immune-polarized LSECs in steatotic livers, which were transduced more readily by AAV5 (Fig. 5g,i). These results indicated that, as with hepatocytes, the steatotic environment increases the transduction of LSECs by AAV5. In addition, these results highlighted LSECs (the physiological source of factor VIII and von Willebrand factor) as an alternative and more physiologically relevant target than hepatocytes for hemophilia A^57^ or von Willebrand disease gene therapy^58,59^. In support of this notion, the transduction efficiency of LSEC subtypes by the AAV8 and AAV5 capsids approached or surpassed levels found in hepatocytes transduced with the same capsids (Figs. 2d and 5i). Moreover, 1.25% of total LSECs expressed *MKI67* or *TOP2A*, which is comparable to the 1.30% of hepatocytes found to express these markers and suggests a similar turnover rate and thus AAV vector persistence between the two cell types^60^ (Extended Data Fig. 9d).

Finally, we sought to define potential host factors involved in or affected by transduction of the five AAV capsids in hepatocytes from NL 3, NL 4, SL 3 and SL 4 (Supplementary Data 1). We found no evidence for activation of inflammatory signaling by AAV vectors in hepatocytes (Supplementary Fig. 8d). Differential gene expression analysis of 3,749 hepatocytes uniquely transduced with one of the five AAV vectors further showed that most upregulated genes exhibit a modest increase in expression (log_2_ (fold change) < 1), indicating that AAV vectors do not perturb the host cell transcriptome (Supplementary Data 1). Focusing on significantly coenriched genes common to at least three capsids highlighted *PIGR*, which regulates the transcytosis of immune complexes for defending against viral infection^61^, and *APOC1*, a cofactor mediating hepatitis C virus infection^62^. *CSH2* was the most significantly coenriched gene in hepatocytes transduced with the AAV8, AAV-LK03 or AAV-NP59 capsid in NL 2, NL 3 and SL 4 (Supplementary Data 1 and Supplementary Fig. 12a,b). Despite evidence that *CSH2* plays a role in regulating hepatitis B virus transcription^63^ or in the virus defense response^64^, its role in AAV vector transduction is unknown.

These results define the human NPC tropism of the four AAV capsids most commonly used in clinical trials of liver gene therapy^1^ and a promising new capsid engineered to specifically transduce human hepatocytes^34^. Illustrating the value of these results, they suggest LSECs as a physiological target for gene therapy of hemophilia A or von Willebrand disease using the AAV5 or AAV8 capsid. These results also highlight capsid-specific genes potentially involved in AAV trafficking or transcription and show transcriptomic stability of hepatocytes transduced with AAV vectors.

## Discussion

Here, we combined long-term NMP under physiological conditions with quantitative analysis of functional transduction at the single-cell level to predict the performance of AAV capsids in the normal and steatotic human liver. We validated our approach by side-by-side comparison of different AAV capsids, using several AAV production services (Supplementary Table 2 and Extended Data Fig. 5a–h) to exclude potential technical bias^65^, with the AAV8 capsid serving as an internal control connecting the different experiments. Our approach bypasses critical limitations to predicting AAV capsid performance based on results of prior clinical trials^3,8^ or preclinical studies in rodents and NHPs^9–13^. Although functional transduction could be maximized by extending NMP duration and increasing AAV vector dosing, the demonstrated robustness of our approach allows for informing and derisking human liver gene therapy.

Notably, we found that, of the AAV capsids most commonly used in clinical trials^1^, AAV-LK03 transduces hepatocytes much more efficiently than AAV8, AAV5 and AAV6 in both normal and steatotic human livers. This result holds in a side-by-side comparison with AAV-NP59, which is better than AAV-LK03 at transducing human hepatocytes engrafted in immune-deficient mice^34^. Moreover, by combining a ubiquitous promoter with single-cell analysis, we found that AAV-LK03 is more specific for hepatocytes, showing little to no transduction of NPCs. Thus, our findings argue for using the AAV-LK03 capsid in clinical trials of hepatocyte-targeted liver gene therapy to maximize therapeutic gene expression and minimize off-target effects such as a heightened immune response.

Our results further show that the AAV-LK03 capsid preferentially transduces periportal hepatocytes in normal livers and is therefore best suited for gene therapy of diseases manifesting specifically in these cells, such as ornithine transcarbamylase deficiency^18^ and phenylketonuria^19^. The AAV5 capsid transduces hepatocytes broadly in normal livers and may therefore, despite lower overall transduction efficiency, be an option for diseases affecting functions outside of the periportal zone^15^. Such pericentrally zonated diseases include acute intermittent porphyria and Crigler–Najjar syndrome, the latter being the subject of an ongoing clinical trial that uses a vector produced with the AAV8 capsid, which we found to have a slight preference for periportal hepatocytes^17^.

A recent study also reported efficient transduction by the AAV-LK03 capsid in human liver maintained by NMP^23^. However, this AAV capsid screen lacks the robustness needed to inform clinical practice because it was performed in a single liver from a donor with biliary sepsis that was surgically split and showed active inflammation. Moreover, whether the human plasma used for perfusion contained NAbs was determined using an in-house assay that did not define AAV capsid-specific reactivity and lacked functional validation, both prerequisites for predicting AAV capsid performance in humans^66,67^. Illustrating the impact of these biases, functional transduction by the FDA-approved AAV5 capsid and most commonly clinically used AAV8 capsid could not be detected in that study. Moreover, altered hemodynamics in the liver halves and redundant vector design prevented detection of zonation of transduction. That study also left open the question of which cell types are transduced. Because only 52% of the cells in the liver are hepatocytes^68^, analyzing transduction by sequencing of bulk liver tissue as performed in that study lacks the specificity needed to identify hepatocyte tropism. By contrast, our approach based on NMP of multiple healthy intact livers under physiological conditions, specifically eliminating NAbs and analyzing functional transduction at single-cell resolution, provided the robustness needed to establish which AAV capsid facilitates the most efficient (and most specific) hepatocyte-targeted gene therapy in the human liver.

Combining a ubiquitous promoter with single-cell analysis further revealed that in normal livers, Kupffer cells are highly transduced by the AAV6 and AAV8 capsids, which points to vector loss and immune response as factors undermining the therapeutic efficacy of vectors produced with these capsids^69^. Conversely, this tropism could be harnessed to weaponize Kupffer cells against liver fibrosis^70^ or cancer^71^. In addition, we found that LSECs are efficiently transduced by AAV5 and AAV8 capsids, which creates an opportunity for improving liver gene therapy of hemophilia A. Because the NPC tropism of AAV capsids in the human liver was previously unknown, clinical trials have invariably been targeting hepatocytes despite LSECs being the physiological source of factor VIII^57^. Unphysiological factor VIII expression in hepatocytes induces an unfolded protein response, which is thought to contribute to progressive loss of therapeutic efficacy in clinical trials^72^. Moreover, LSEC targeting overcomes a roadblock to gene therapy of von Willebrand disease caused by the inability of hepatocytes to multimerize von Willebrand factor protein, which is required for long-term functionality^58,59^. Our findings suggest AAV5 or AAV8 capsid-mediated LSEC-targeted gene therapy as a potential solution to these problems, given that LSECs show transduction and proliferation rates comparable to hepatocytes in human liver^60^.

We found that steatosis alters the hepatocyte tropism of AAV capsids, with the AAV-LK03 and AAV8 capsids losing periportal zonation and the AAV5 capsid gaining pericentral zonation. Even highly steatotic hepatocytes were susceptible to AAV transduction, particularly by the AAV5 capsid, suggesting an opportunity for therapy of steatotic liver disease^41,73^. Moreover, AAV5 outperforms the other capsids in transducing LSECs in steatotic livers, as opposed to normal livers, where its transduction efficiency matches that of AAV8. These results inform the design of a potential LSEC-targeted AAV gene therapy in the overweight hemophilic population^74^.

In attempting to better understand what causes AAV capsid-specific differences in functional transduction, we unexpectedly found that the capsid protein contributes to vector episome formation. Episomes are primarily formed by recombination of ITRs, a process thought to be regulated by ITR binding to cellular proteins related to DNA damage repair pathways, such as nonhomologous end joining (NHEJ) and homologous recombination (HR)^38,55^. Our analyses in human livers and immune-deficient mice engrafted with human hepatocytes show that the AAV5 capsid, which is phylogenetically different from other wild-type capsids^8^, participates in the process of ITR recombination differently than the AAV8 and AAV-LK03 capsids, promoting the formation of circular monomers and delaying their concatemerization. This finding is consistent with results from a previous study that excluded that the ITR sequence determines episome conformation^75^.

Although recent studies have speculated that degradation of AAV8 and AAVrh10 episomes is caused by host immune responses in NHP liver^76^ or that failed chromatinization of AAV-LK03 episomes leads to their degradation in mouse liver^37^, we found that only AAV5 episomes rapidly degrade in human hepatocytes engrafted in immune-deficient mice. Therefore, it appears worthwhile to investigate alternative mechanisms such as epigenetic modulation by which different AAV capsids impact episome circularization and persistence in human hepatocytes, also to facilitate the development of more durable AAV vectors.

By comparing normal and steatotic hepatocytes, we found that excessive fatty acids interfere with the circularization of AAV vectors. Steatosis induces oxidative stress, which triggers the accumulation of DNA damage that can overwhelm or inhibit repair mechanisms including NHEJ and HR^77,78^. Lack or inactivity of components of the NHEJ and HR pathways needed for ITR recombination explains impaired episome formation of AAV vectors in steatotic hepatocytes. Therefore, the severity of an individual’s hepatic steatosis should be taken into account to achieve long-term therapeutic efficacy of AAV gene therapy. In addition, DNA recombination-dependent strategies, such as delivery of large genes with dual AAV vectors^79^ and Cas9-mediated gene editing using templates delivered with AAV vectors^80^, will probably be less effective in these individuals.

Our scRNA-seq-based approach to side-by-side comparison of functional transduction by AAV capsids could be used to screen large capsid libraries in normal or diseased human livers. In addition to optimizing hepatocyte transduction, such a screening could facilitate gene delivery to other liver cell types, for example, cholangiocytes to correct inherited biliary diseases^81^ or activated hepatic stellate cells to reverse liver fibrosis^82^.

On the basis of our findings, we can suggest the following four ways to advance human liver gene therapy: (1) prioritize the AAV-LK03 capsid for efficient and specific transduction of hepatocytes, which may facilitate vector dose reduction, thereby minimizing the risk of hepatotoxicity^8^ and genomic integration of the vector^83^; (2) increase therapeutic efficacy by meeting disease-specific demands such as targeting of periportal hepatocytes with AAV-LK03 in normal liver and pericentral hepatocytes or LSECs with AAV5 in steatotic liver; (3) consider the durability of transgene expression in clinical trial design, for example, taking into account that episome formation of AAV vectors is impaired in steatotic liver; and (4) use the near-clinical setting provided by human liver NMP to develop new AAV capsids that transduce hepatocytes or other therapeutically relevant cell types in all individuals with maximum efficiency and specificity.

## Methods

### Long-term NMP of human liver

Human livers declined for transplantation were obtained through regional Organ Procurement Organizations from donation after brain death and donation after circulatory death. Informed consent from decedents was obtained by the Organ Procurement Organizations. The University of California, San Francisco (UCSF) Institutional Review Board Committee for Human Research Protection Program determined that this study was exempt from Institutional Review Board review as it does not meet the FDA’s definition of a clinical investigation involving human subjects (21 CFR 50.3 and 21 CFR 812.3). Discarded livers from donors following circulatory death were included if they met the following criteria: warm ischemia time of ≤60 min (measured from the time systolic blood pressure dropped below 80 mm Hg or oxygen saturation dropped below 80%), liver extraction time (cross-clamp to liver recovery) of ≤50 min and projected cold ischemia time of ≤12 h. Liver extraction time and cold ischemia time for discarded livers from donors following brain death were limited to ≤65 min and ≤16 h, respectively. Livers were procured and packaged by the procurement surgeon as if they were being used for transplant.

For long-term NMP, livers were connected to the OrganOx metra according to the manufacturer’s instructions^84^ with modifications. On the back table, portal vein, hepatic artery, inferior vena cava and bile duct were cannulated. One to 2 l of lactated Ringer’s solution (B. Braun) and 500 ml of 5% human albumin (Grifols) were then used to flush out cold University of Wisconsin (UW) solution (Bridge to Life) from the liver via portal vein and hepatic artery cannulas before connection to the device. The device circuit was primed with 500 ml of 5% human albumin and 3 U of type-specific PRBCs obtained from the UCSF Blood Bank. Once the temperature of the perfusate reached 37 °C, boluses of 10,000 U of heparin (Hospira) or 50 mg of enoxaparin (Amphastar Pharmaceuticals), 750 mg of cefuroxime (Hikma Pharmaceuticals) and 10 ml of 10% calcium gluconate (Fresenius Kabi) were administered. When the pH of the perfusate reached 7.3 after addition of 20–30 ml of 8.4% sodium bicarbonate (Hospira), the liver was connected to the circuit. Four medications (0.18 g ml^–1^ sodium taurocholate (OrganOx), 3.3 U ml^–1^ insulin (Lilly), 833 U ml^–1^ heparin or 4.2 mg ml^–1^ enoxaparin and 8.3 µg ml^–1^ epoprostenol sodium (Flolan, GlaxoSmithKline)) were constantly infused by infusion pumps at a speed of 1 ml h^–1^ throughout the NMP. When the glucose level first reached 100 mg dl^–1^, parenteral nutrition solution (Clinimix E 5/20, Baxter) was infused to maintain its level at 100–200 mg dl^–1^, and the amount of insulin being continuously infused into the perfusate was adjusted according to glucose levels.

A hemoconcentrator (Hemocor HPH Junior, Minntech) was added between the centrifugal pump and the perfusate reservoir and kept excluded from the circuit with a tubing clamp. The molecular weight cutoff of the hemoconcentrator was sufficiently small to minimize the loss of AAV particles from the circuit^85^ (65 kDa versus 3,740 kDa). To avoid potential interference with AAV transduction, the first filtration was performed at least 12 h after the AAV vectors were infused. Every 6–12 h, perfusate was filtered through the hemoconcentrator to remove metabolic waste for 60–120 min. After each filtration, dialysate (Duosol 4553, B. Braun) was added to the reservoir to replace the volume lost during filtration and improve electrolyte composition. Hemofiltrate was collected in a urinary drain bag (Medline) to analyze the presence of AAV vgs.

In response to the expected gradual hemolysis of PRBCs in the circuit, perfusate was partially exchanged by adding 1–2 U of PRBCs into the reservoir when hemoglobin fell below 6 g dl^–1^ or every 24 h. When PRBCs were added, 5% human albumin, heparin or enoxaparin, cefuroxime, calcium gluconate and 8.4% sodium bicarbonate were supplemented proportionally.

### Monitoring of NMP

A liver tissue biopsy was taken before NMP using a 16-gauge biopsy needle (Temno, Merit Medical) for histological analysis. The tissue sample was stored in 10% neutral buffered formalin (Sigma-Aldrich) overnight and sent to Peninsula Histopathology Laboratory for paraffin embedding and sectioning.

The perfusate was collected from the hepatic artery line for analysis. Arterial blood gas analysis and measurement of lactate, electrolytes, glucose, blood urea nitrogen, creatinine and hemoglobin were performed using CG4+ and Chem8+ cartridges (Abbott) with an i-STAT1 analyzer (Abbott). Total bilirubin, total protein, albumin, alkaline phosphatase, aspartate transaminase, alanine transaminase and osmolality were analyzed at the UCSF Clinical Laboratories. For frequent glucose measurements, 1 µl of perfusate was analyzed using a glucometer (Contour Next Blood Glucose Monitoring System, Ascensia Diabetes Care)^86^. Arterial pressure and portal, arterial and inferior vena cava flow were continuously recorded by the OrganOx metra device. Bile was serially collected every 4–6 h from the biliary cannula to measure the production rate, and samples were stored at −80 °C. Three milliliters of perfusate was centrifuged at 2,000*g* for 10 min at 4 °C, and a fraction of supernatant plasma was stored at −80 °C. Thawed plasma was used to analyze the levels of factor V (Human Factor V ELISA Kit, Abcam), alanine transaminase (Human ALT SimpleStep ELISA Kit, Abcam), lactate dehydrogenase (Lactate Dehydrogenase Assay Kit, Abcam) and uric acid (Uric Acid Assay Kit, Abcam). AAV vgs were analyzed by ddPCR after DNA isolation from the plasma and bile.

### Isolation of human liver cells

After termination of NMP, the liver was cleared of perfusate by flushing with 10–15 l of ice-cold 0.9% NaCl solution (Baxter) through the portal vein and hepatic artery until the efflux was clear without any traces of blood. One liter of UW solution was then infused to maintain cell viability during sample processing. Fifty- to 70-g wedges of the right and left liver lobes were cut and immediately immersed in ice-cold UW solution. Sections were further flushed by manual perfusion of UW solution through open vessels before cell release. To release hepatocytes, sections were perfused with calcium- and magnesium-free HBSS containing 20 mM HEPES, penicillin–streptomycin (100 U ml^–1^ and 100 µg ml^–1^, respectively) and 1 mM EGTA (all Gibco) for 15 min at 37 °C. Sections were then perfused with 1 mg ml^–1^ collagenase IV (Worthington) in HBSS containing 20 mM HEPES, penicillin–streptomycin and 5 mM CaCl_2_·H_2_O (Thermo Fisher Scientific) for 22 min at 37 °C. The crude cell preparation was suspended in DMEM (Thermo Fisher Scientific) containing penicillin–streptomycin and 5% fetal bovine serum (Corning), filtered through sterile gauze (Covidien), pelleted at 70*g* and washed twice with the same suspension medium. Hepatocytes were further purified by centrifugation through 45% Percoll (Cytiva) at 200*g* for 15 min. Hepatocytes isolated in this fashion were used for flow cytometry/fluorescence-activated cell sorting (FACS) and scRNA-seq. NPCs were retrieved from the hepatocyte supernatant fraction from the first centrifugation at 70*g* and reserved for flow cytometry. For dedicated isolation of NPCs, a section was perfused for 15 min with HBSS containing 1 mM EGTA, followed by perfusion with 300 ml of 1 mg ml^–1^ pronase (Roche) in DMEM and subsequently with 1.5 mg ml^–1^ collagenase IV in HBSS containing 5 mM CaCl_2_·H_2_O for 22 min. The crude cell preparation was filtered through sterile gauze and digested further in 200 ml of 0.5 mg ml^–1^ pronase for 30 min at 37 °C with constant shaking. The cell suspension was pelleted at 600*g*, washed twice and subjected to density gradient centrifugation with 9% Accudenz (Accurate Chemical) at 1,400*g* for 17 min. Hepatic stellate cells were retrieved from the top of the 9% Accudenz gradient, and other NPC populations were retrieved from the midportion of the gradient. Both populations were washed and processed further for flow cytometry/FACS and scRNA-seq.

For scRNA-seq of NL 2 (one array), NL 3 (two arrays), NL 4 (one array) and SL 3 (two arrays), some cells were collected via ‘chunk’ digestion, which enriches for immune cell populations such as T cells, as previously described^87^. Briefly, a ~250-mg tissue sample was minced and incubated in Liver Perfusion Medium (Gibco) at 37 °C for 15 min with rotation at 20 rpm. After washing with PBS, the tissue sample was incubated with at least 2.4 mg ml^–1^ collagenase IV in HBSS supplemented with 1% HEPES for 30 min at 37 °C with rotation at 100 rpm. The tissue sample was then triturated using a 10- or 25-ml pipette 10–15 times and passed through a 70-µm filter to acquire single cells. RBCs were lysed with ACK Lysis Buffer (Gibco), viability was assessed using trypan blue (Thermo Fisher Scientific), and cells were counted with a hemocytometer (Hausser Scientific).

### Nuclease protection assay

Nuclei were isolated from frozen human liver tissue samples using Nuclei EZ Prep (Sigma-Aldrich), according to the manufacturer’s instructions, and washed once with resuspension buffer consisting of 0.01% bovine serum albumin in PBS (both Thermo Fisher Scientific). To analyze the amount of encapsidated AAV vgs, nuclei were resuspended in 200 µl of benzonase buffer (2 mM MgCl_2_ and 20 mM Tris-HCl (pH 8.0); both Thermo Fisher Scientific) and divided equally into two tubes. One tube was treated with 250 U of benzonase (Sigma-Aldrich) for 1 h at 37 °C, and one tube was mock treated. After digestion, 10 µl of 50 mM EDTA (Thermo Fisher Scientific) was added to inactivate the benzonase. The samples were then digested with 10 U of proteinase K (Thermo Fisher Scientific) for 1 h at 56 °C, followed by DNA extraction using a QIAamp DNA Mini Kit. Benzonase- and mock-treated samples were subjected to ddPCR to measure the copy number of encapsidated AAV vgs and total AAV vgs, respectively. The copy number of uncoated AAV vgs was obtained by subtracting the copy number of encapsidated AAV vgs from the copy number of total AAV vgs.

To accurately analyze the amount of circular episomes, DNA was extracted using a noncolumn method (MasterPure Complete DNA and RNA Purification Kit; LGC Biosearch Technologies) according to the manufacturer’s instructions^88^. DNA was lysed and extracted from cells, tissues or tissue-derived nuclei without vortexing to avoid DNA shearing. Linear double-stranded DNA was hydrolyzed by mixing 1,000 ng of DNA with 0.5 U µl^–1^ of PS-DNase, 1 mM ATP and buffer (33 mM Tris-acetate, 66 mM potassium acetate, 10 mM magnesium acetate and 0.5 mM DTT) in a final volume of 100 µl (all LGC Biosearch Technologies) and incubating for 17 h at 37 °C. The sample was then incubated for 20 min at 80 °C to deactivate PS-DNase and used to measure the amount of episomes. The same amount of DNA, mock treated without PS-DNase, was used to quantify genomic DNA. To cut the episomes and separate out linear AAV vgs, half of the episome sample was cotreated with 2.5 U of XbaI and 2.5 U of SphI or 2.5 U of SphI and 2.5 U of BmtI (all New England Biolabs) for 30 min at 37 °C, whereas the other half was mock treated.

To analyze the amount of circular episomes using T5 exonuclease, DNA was extracted from tissues or tissue-derived nuclei and divided into two tubes. Next, 20 U of T5 exonuclease and buffer (20 mM Tris-acetate, 50 mM potassium acetate, 10 mM magnesium acetate and 1 mM DTT) were added to one tube in a final volume of 50 µl (both New England Biolabs) for 1 h at 37 °C, whereas the other tube was mock treated. After digestion, the exonuclease was inactivated by adding EDTA to a final concentration of 14 mM and incubating for 15 min at 70 °C.

To measure the amount of DNA using nanoplate-based digital PCR, the reaction mixture was made by combining QIAcuity Probe PCR Kit mastermix (Qiagen), 800 nM forward and reverse primer, 400 nM probe and DNA template in a final volume of 12 µl. The mixture was transferred to QIAcuity Nanoplate 8.5k 96-well reaction plates (Qiagen) for partitioning. PCR amplification was performed in a QIAcuity One 5plex instrument (Qiagen) using the following program: enzyme activation at 95 °C for 2 min and amplification at 95 °C for 15 s and 56 °C for 30 s, repeated for 40 cycles. Partitions were imaged with a 500-ms exposure time for FAM and HEX channels, and copy concentration was measured using QIAcuity Software Suite (Qiagen, v2.1.7).

### scRNA-seq

The S^3^ SeqWell platform was used to perform scRNA-seq^87,89,90^. Isolated hepatocytes and NPCs were separated by digestion fraction, and viability and cell density were assessed using trypan blue and a hemocytometer. Cell suspensions were diluted to a concentration of 20,000–30,000 cells per 300 µl, as recommended for this platform, and loaded onto individual arrays (NL 2 = six arrays, NL 3 = eight arrays, NL 4 = eight arrays, SL 3 = four arrays, SL 4 = eight arrays) containing approximately 110,000 barcoded mRNA capture beads (ChemGenes). Arrays were sealed with functionalized polycarbonate membranes (Sterlitech) in a clamp with a glass-sealing slide (Corning) at 37 °C for 40 min. Arrays were freed from clamps and bathed in lysis buffer (5 M guanidine thiocyanate, 1 mM EDTA, 0.5% Sarkosyl and 1% 2-mercaptoethanol; Thermo Fisher Scientific and Sigma-Aldrich) for 5–10 min until glass-sealing slides detached. Arrays were left submerged in lysis buffer for 20 min, washed with hybridization buffer (2 M NaCl and 4% PEG8000; both Sigma-Aldrich) once and incubated in hybridization buffer for 40 min. All lysis and hybridization buffer steps were performed at room temperature with gentle *xy* plane rotation. Arrays were washed five to ten times with wash buffer (2 M NaCl, 3 mM MgCl_2_, 20 mM Tris-HCl (pH 8.0) and 4% PEG8000) and scraped ten or more times with a glass slide to collect beads in 50-ml conical tubes. Beads from different arrays were collected and processed separately.

Beads were processed through reverse transcription, exonuclease treatment, second-strand synthesis, whole-transcriptome amplification, solid-phase reversible immobilization bead purification and library preparation as previously described^87^. For NL 2, NL 3, NL 4, SL 3 and SL 4 sample libraries, 6 nM dilutions were prepared. For SL 1 sample libraries, 4 nM dilutions were prepared. Dilutions were pooled for sequencing on a NovaSeq 6000 SP 100 flow cell (Illumina) at the UCSF Center for Advanced Technology Core (NL 2, NL 3, NL 4, SL 3 and SL 4) or on a NovaSeq 6000 S4 flow cell (Illumina) at the Chan Zuckerberg BioHub (SL 1). The sequenced data were preprocessed and aligned using the dropseq workflow on Terra (app.terra.bio).

### Custom genome preparation

Custom genomes were prepared following the 10x Genomics ‘Build a Custom Reference’ tutorial (https://support.10xgenomics.com/single-cell-gene-expression/software/pipelines/latest/using/tutorial_mr). Unique fasta and gtf files were generated for transgenes (both fluorescent protein and barcode sequences) and barcodes (barcode sequence only). Fasta files were appended to the hg38 reference genome (*Homo sapiens* GRCh38.p13) fasta as unique ‘pseudochromosomes’, and gtf files were appended to the hg38 reference gtf as unique ‘protein coding’ entries. The resulting customized hg38 fasta and gtf files were used to generate a custom human reference genome used for STAR alignment.

### Sequencing, alignment to custom genome and barcode detection

Sequencing results were returned as paired FASTQ reads and aligned using STAR aligner against the appropriate custom hg38 reference genomes following the dropseq workflow (https://cumulus.readthedocs.io/en/latest/drop_seq.html). NL 4 and SL 4 sequencing results were aligned with the following considerations. To account for mCardinal and mTagBFP2 sharing approximately 95% sequence homology, a second STAR alignment was performed using –outFilterMultimapNmax = 1 to prevent read mismapping. From this alignment, reads for mCardinal and mTagBFP2 were extracted from the digital gene expression counts matrix and merged with the primary alignment. To improve detection of the shorter barcode sequences, a third STAR alignment was performed using –outFilterMatchNmin = 20. Cell IDs containing reads for barcodes 1–4 were extracted from the digital gene expression counts matrix resulting from this alignment and annotated in the object metadata or, in the case of NL 4, merged with the primary alignment. Calculations involving transduction efficiency of the AAV vectors used in this study included barcode-expressing cells.

### Hepatocyte subclustering analysis and gene module scoring

For each human liver, clusters identified as ‘hepatocytes’ were subset based on liver of origin and processed as described in ‘Single-cell clustering analysis’ in the Supplementary Methods. Briefly, variable features were calculated for each subset before integration, followed by scaling and principal component analysis. Fluorescent protein and barcode genes were removed from both integration features and the list of variably expressed genes used to perform the principal component analysis. Gene module scoring was performed using the UCell (v2.5.1) package^91^, which relies only on the relative expression of genes within individual cells, meaning that variation in gene expression levels between individual livers does not affect the score given to each cell in an integrated dataset. Periportal and pericentral gene module scores were determined based on zonation marker genes conserved across NL 3, NL 4, SL 3 and SL 4 after integration. The steatosis gene module score was determined based on hepatocyte gene expression in NL 3, NL 4, SL 3 and SL 4, taking into consideration histologic scoring of steatosis, and cross-referenced with existing literature^92–95^. To generate kernel density estimation histograms of transgene-expressing cells versus gene module score, transgene-expressing cells that had a score for a given module of greater than 0 were used. Periportal and pericentral gene module scores corresponding to the tallest peak were used to determine zonation enrichment. In the case of bimodal distributions in Fig. 3c,f and Extended Data Fig. 7f, the peak height and gene module scores of the major and minor modes were considered. If the score of the major mode peak was greater than that of the minor mode and the major mode peak height surpassed that of the minor mode by greater than 20%, zonation was considered enriched. If the major and minor mode peaks differed by less than 20%, zonation was considered weakly enriched. Multimodal or ‘plateau’ distributions were considered weakly enriched if the score of the major mode peak was greater than those of the minor modes. The ggplot2 (v3.4.4) package was used to generate scaled density plots and calculate distribution amplitudes. Enriched expression of hepatocyte zonation marker genes and the SOX9^+^SPP1^+^TACSTD2^+^ subset were visualized using the Nebulosa (v1.4.0) package^96^.

### Statistics and reproducibility

In all validation experiments involving mice and all ddPCR and digital PCR analyses, the significance of differences between groups was determined by two-tailed unpaired *t*-tests or two-way analysis of variance with Tukey’s post hoc test, and data were normally distributed. For scRNA-seq analysis, the significance of cluster-specific markers was determined using Wilcoxon rank-sum test, and *P* values were adjusted using Bonferroni’s correction. For determination of differentially expressed genes in AAV-transduced cells, the Wilcoxon rank-sum test was performed, and *P* values were adjusted using the Benjamini–Hochberg procedure. Pearson correlation coefficients with two-sided *P* values were calculated for scatter plots using the stat_cor function in the ggpubr (v0.6.0) package. Technical and biological replicates are indicated in the figure legends and text. At least two independent experiments were performed for all micrograph images. Plots were generated using GraphPad Prism (v9.4.1).

### Reporting summary

Further information on research design is available in the Nature Portfolio Reporting Summary linked to this article.

## Online content

Any methods, additional references, Nature Portfolio reporting summaries, source data, extended data, supplementary information, acknowledgements, peer review information; details of author contributions and competing interests; and statements of data and code availability are available at 10.1038/s41587-024-02523-6.

## Supplementary information

Supplementary Information Supplementary Methods, Figs. 1–12, Tables 1–5, Data 1 legend and References.

Reporting Summary

Supplementary Data 1 Co-regulated genes in AAV-transduced hepatocytes. Differentially expressed genes in hepatocytes transduced with AAV vectors (tabs 1–5) and similarities in differentially expressed genes between AAV vectors (tab 6, gray boxes indicate enrichment); BC, barcode. *P* values less than 0.05 calculated using Wilcoxon rank-sum test and adjusted with Benjamini–Hochberg procedure are shown.

## Source data

Source Data Extended Data Fig. 6 Violin plot source data for Extended Data Fig. 6c.

## Figures and Tables

**Extended Data Table 1:**
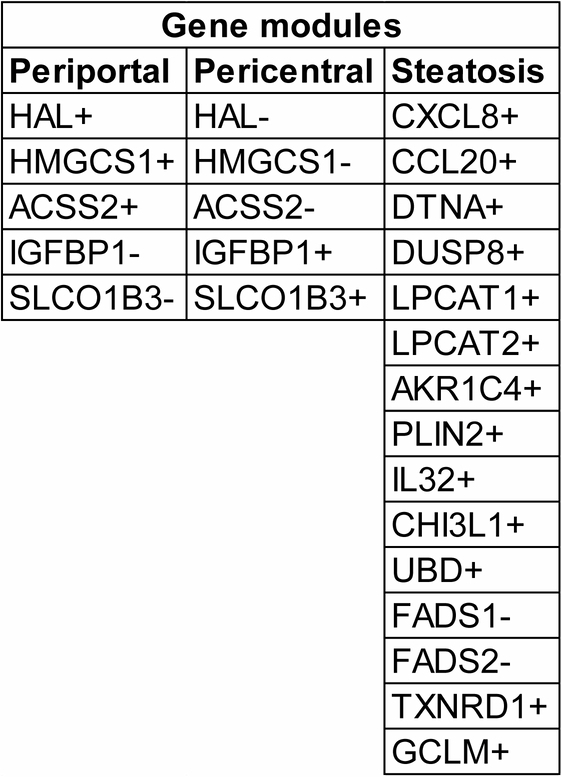
Gene modules

Gene modules used to score periportal, pericentral and steatotic hepatocytes. A “+” or “−” sign indicates the gene is upregulated or downregulated in the module, respectively.

## Data Availability

Raw scRNA-seq FASTQ files and processed digital gene expression matrices generated in this study are deposited in the Gene Expression Omnibus under the accession number GSE228000. Source data are provided with this paper. Additional data are available from the corresponding author upon reasonable request.

## Code availability

All software algorithms used for analysis are specified where applicable and were accessed through public repositories. No new software was developed for this study.
