# Supplementary material for: AAV capsid prioritization in normal and steatotic human livers maintained by machine perfusion
Source: Nat Biotechnol. 2025 Jan 29;43(12):1966–78. doi: 10.1038/s41587-024-02523-6 (PMC12304247; doi:10.1038/s41587-024-02523-6)
Supplement: Supplementary file 1 — Supplementary Methods, Figs. 1–12, Tables 1–5, Data 1 legend and References. [file 41587_2024_2523_MOESM1_ESM.pdf]

nature biotechnology

Supplementary information https://doi.org/10.1038/s41587-024-02523-6

AAV capsid prioritization in normal and steatotic human livers maintained by machine perfusion

In the format provided by the authors and unedited

Table of Contents

Supplementary Methods

Supplementary Figs. 1-12

Supplementary Tables 1-5

Supplementary Data 1 legend

Supplementary References

Supplementary Methods

Washing of PRBCs

To establish removal of NAbs to AAV capsids contained in plasma, PRBCs were washed manually using two centrifugation-mediated washing protocols. In the first protocol[1], half a unit of PRBCs was mixed with 350 ml of 0.9% NaCl solution and centrifuged at 4,950*g* for 15 min at 4 °C, which was repeated once. In the second protocol[2], half a unit of PRBCs was mixed with 5% human albumin at a 4:1 ratio and centrifuged at 2,500*g* for 5 min at 4 °C. After washing, supernatant was collected for analysis of titers of NAbs to AAV capsids. Automated washing of PRBCs with 1-2 l of 0.9% NaCl solution per unit was performed at the UCSF Blood Bank using a COBE 2991 Cell Processor (Terumo BCT) immediately before NMP.

Measurement of NAbs to AAV capsids

The quantity of NAbs to individual AAV capsids was measured in plasma from PRBCs or NMP perfusate. A luciferase-based in vitro neutralization assay was performed according to a previously reported protocol[3] with these modifications: (1) different cell lines were used to account for capsid-specific in vitro transduction efficiency: HeLaRC32 cells (ATCC) for AAV2, AAV5 and AAV-DJ at multiplicities of infection of 5,000, 100,000 and 10,000, respectively; HeLaRC32 cells stably expressing human AAVR[4] from an integrated plasmid for AAV6 and AAV8 at multiplicities of infection of 100,000 and 50,000, respectively; (2) in every assay, human intravenous immunoglobulin (Gamunex, Grifols) was used as a standard; and (3) wild-type human adenovirus type 5 (ATCC) was added during the last 24 h of the assay at multiplicity of infection of 50 to induce AAV replication.

AAV vector production and titration

Recombinant AAV vectors containing fluorescent genes were produced by several vendors (Supplementary Table 2). Titers were determined according to a previously reported protocol[5] modified as follows: AAV vectors were digested with DNase I (Qiagen) at a concentration of 300 U ml^{-1} for 30 min at 37 °C. Then, samples were serially diluted with dilution buffer consisting of 0.001% Pluronic F-68 (Gibco) in water. The reaction mixture was made by adding ddPCR Supermix for Probes (Bio-Rad) and CMV primer-probe sets in a final volume of 20 µl. Droplets generated by a QX200 droplet generator (Bio-Rad) were PCR amplified using the following program: denaturation at 95 °C for 10 min and amplification at 94 °C for 30 s, 57 °C for 1 min and 72 °C for 15 s, which

was repeated for 40 cycles, followed by heat inactivation at 98 °C for 10 min. The concentration of copies was measured using a QX100 droplet reader (Bio-Rad) and analyzed using QuantaSoft software (Bio-Rad, v1.7).

AAV vector infusion into human liver

Thawed AAV vectors were reconstituted in injection buffer (20 mM Tris pH 8.0, 1 mM $MgCl_2$, 200 mM NaCl and 0.001% Pluronic F-68 in water) in a final volume of 5-10 ml. AAV vectors were infused once the hemodynamics were stabilized and liver functionality was confirmed by perfusate parameters, 6-12 h after NMP began. A syringe with a 27-gauge needle was used to inject AAV vectors over the course of 1 min directly into the portal vein.

Flow cytometry of human liver cells

Human liver cells were incubated with Fc receptor blocker (Human TruStain FcX, Biolegend) for 10 min at 4 °C. For antibody surface staining, cells were incubated with antibody cocktails (Supplementary Table 3) in DMEM buffer (2% fetal bovine serum in DMEM) for 30 min at 4 °C. Then, cells were washed and resuspended in DMEM buffer and kept at 4 °C until analysis. Before analysis, SYTOX Dead Cell Stain (Thermo Fisher Scientific) was added at a 1:1,000 dilution to exclude dead cells. To prepare single-color compensation controls, frozen human liver cells were thawed and stained with appropriate antibodies. As compensation controls for fluorescent proteins, HEK293 cells (ATCC) were transfected with plasmids encoding fluorescent proteins. Stained cells were analyzed and sorted on a FACSAria II (BD) using FACSDiva software (BD, v9.4) and plots were acquired using FlowJo software (BD, v10.9.0).

Immunofluorescence and histological stainings of human liver

Liver tissue was cut in small pieces (<0.5 cm) and placed in 4% paraformaldehyde (Electron Microscopy Sciences) or 10% neutral buffered formalin for fixation overnight. Some tissue samples were directly frozen in liquid nitrogen for cryopreservation.

Tissue samples fixed in 4% paraformaldehyde were kept in 30% sucrose (Sigma-Aldrich) solution overnight, embedded in O.C.T. medium (Fisher Healthcare) and frozen at -80 °C. Frozen tissue samples were cut at 4 µm thickness using a Leica CM1950 cryostat. Frozen sections were permeabilized in 0.1% Triton X-100 (Sigma-Aldrich) and blocked in 5% normal donkey serum (Jackson ImmunoResearch Laboratories). Sections were then submerged in bleaching solution consisting of 4.5% hydrogen peroxide and 20 mM sodium hydroxide (Sigma-Aldrich) in PBS, and kept under a bright LED lamp (Fitfirst) overnight at 4 °C to reduce autofluorescence. After

washing with 0.1% Tween-20 (Sigma-Aldrich) in PBS, sections were incubated with primary antibodies and fluorophore-labeled secondary antibodies (Supplementary Table 3). When sections were costained with Bodipy 493/503 (Thermo Fisher Scientific), antibodies were first applied in blocking buffer consisting of 0.1% saponin (Sigma-Aldrich) and 5% donkey serum in PBS, and then 2.5 μM Bodipy was applied for 30 min. Subsequently, sections were stained with DAPI (BD) and mounted with FluorSave reagent (Millipore). Images were taken with a Leica TCS SP5 confocal microscope using Leica Application Suite X (v4.6.1.27508) and analyzed with Fiji (ImageJ 1.53q).

Tissue samples fixed in 10% neutral buffered formalin were processed, paraffin embedded, cut and stained with Hematoxylin and Eosin (H&E) or Sirius Red by Peninsula Histopathology Laboratory. Oil Red O staining was performed on cryosections using the Oil Red O Stain Kit (Abcam) according to the manufacturer's instructions. Steatosis and fibrosis scores were determined by a UCSF clinical liver pathologist. In situ TUNEL assay was performed on formalin-fixed paraffin-embedded tissues using Click-iT Plus TUNEL Assay Kits for In Situ Apoptosis Detection (Thermo Fisher Scientific) according to the manufacturer's instructions.

Intravenous injection of AAV vectors into mice

AAV vectors were diluted in 100 μl injection buffer (20 mM Tris pH 8.0, 1 mM $MgCl_2$, 200 mM NaCl and 0.001% Pluronic F-68 in water) for single-vector injection or in 200 μl for co-injection of vectors. AAV vectors were injected through the tail vein or retro-orbital sinus while the mouse was anesthetized with isoflurane. 9-12-week-old male wild-type mice (C57BL/6J, Jackson Laboratory) and 24-30-week-old male and female FRGN ($Fah^{-/-}$;$Rag2^{-/-}$;$Il2rg^{-/-}$;$Sirpa^{NOD/NOD}$) mice[6] transplanted with human hepatocytes were used to analyze AAV vector transduction. AAV vector injection and maintenance of these mice were performed at UCSF. All procedures were approved by the Institutional Animal Care and Use Committee at UCSF.

For the study of AAV vector episomes in FRGN mouse livers repopulated to more than 90% (serum human albumin levels 7.8-10.8 mg ml^{-1}) with human hepatocytes, the mice continuously received 8 mg l^{-1} 2-(2-nitro-4-trifluoromethylbenzoyl)-1,3-cyclohexanedione (NTBC) in the drinking water, starting 2 days prior to AAV vector injection until the end of the analysis period. AAV vector injection and maintenance of these mice were performed at OHSU. All procedures were approved by the Institutional Animal Care and Use Committee at OHSU.

Mice at both institutions were housed in a barrier animal facility under standard conditions (12-h light/12-h dark cycle, 30-70% humidity, 20-26 °C temperature).

Isolation and flow cytometry of mouse liver cells

The right lobe of the mouse liver was resected for immunofluorescence by ligating the vessels with a 5-0 suture (Ethilon, Ethicon) and cutting the lobe above the ligation. The remaining liver tissue was dissociated by perfusing collagenase II (Worthington) in HBSS through the inferior vena cava following a previously reported protocol[7]. Cells were collected by centrifugation at 50*g* for 2 min and pelleted cells were subjected to Percoll density gradient centrifugation to isolate viable hepatocytes. Pelleted hepatocytes were stained with antibodies for NPC markers to exclude contaminating NPCs or human-specific antibody for β2-microglobulin to separate human from mouse cells (Supplementary Table 3). Stained cells were analyzed on a FACSAria II and plots were acquired using FlowJo software.

Immunofluorescence of mouse liver

Liver tissue samples were fixed in 4% paraformaldehyde overnight at 4 °C. After washing 3 times with PBS, tissue samples were transferred to 30% sucrose solution and incubated overnight at 4 °C. Then, tissue samples were embedded in O.C.T. medium at -80 °C for cryopreservation and cut at 4 μm thickness using a Leica CM1950 cryostat. Immunofluorescence was performed as described above using antibodies listed in Supplementary Table 3.

Testing of anticoagulants in mice

The anticoagulants heparin, enoxaparin and bivalirudin (Dr. Reddy's) were reconstituted in 0.9% NaCl solution and injected into mice through the retro-orbital sinus at a dose of 5,000 U kg^{-1}, 2 mg kg^{-1} or 2.5 mg kg^{-1}, respectively. After 30 min, 100 μl of AAV vector were injected through the tail vein. Liver tissue samples were resected for immunofluorescence and perfusion digested for flow cytometry as described above.

Testing of AAV vector attachment to tubing

AAV vectors were mixed in 40 ml of DMEM reconstituted with 5% human albumin and 0.03 mg ml^{-1} enoxaparin. The mixture was evenly divided into two 50 ml polypropylene tubes and placed in a 37 °C water bath. The medium from one tube was continuously perfused in a closed circuit through silicone tubing (Masterflex), powered by a perfusion machine (Watson Marlow). At specific time intervals, 200 μl samples were taken from

both the nonperfused and perfused media and stored at -80 °C. DNA isolated from media was analyzed using ddPCR.

In situ hybridization of AAV vector DNA and mRNA

The RNAscope Multiplex Fluorescent Reagent Kit v2 (ACDBio) and BaseScope Duplex Reagent Kit (ACDBio) were used to detect AAV vector DNA and mRNA in formalin-fixed paraffin-embedded human liver tissue. For RNAscope Multiplex Fluorescent Reagent Kit v2, sections cut at 5 μm thickness were incubated in target retrieval solution (ACDBio) for 30 min at 95 °C and blocked with 5% donkey serum for 1 h. Sections were then submerged in bleaching solution and kept under a bright LED light lamp overnight at 4 °C to reduce autofluorescence. For codetection of protein targets, sections were incubated with primary antibodies (Supplementary Table 3) overnight and fixed in 10% neutral buffered formalin before treatment with protease plus (ACDBio), which was followed by hybridization, amplification and fluorescent labeling of probes (Supplementary Table 4). Finally, fluorophore-conjugated secondary antibodies (Supplementary Table 3) were incubated for 1 h followed by DAPI staining. The BaseScope Duplex Reagent Kit was used according to the manufacturer's instructions. Images were taken with a Leica TCS SP5 confocal microscope using Leica Application Suite X and analyzed with Fiji (ImageJ 1.53q).

Cell culture

Huh-7 cells were purchased from the Japanese Collection of Research Bioresources (JCRB) Cell Bank and maintained in RPMI 1640 medium (Thermo Fisher Scientific) containing 5% fetal bovine serum and 1% Antibiotic-Antimycotic Solution (Corning) at 37 °C in 5% CO_2. Human iPS cell-Heps were generated following a previously reported protocol[8]. From day 22 of differentiation, cells were treated every 2 days with palmitic acid conjugated with bovine serum albumin at a molar ratio of 5:1.

DNA isolation and vg copy number analysis

DNA was isolated from frozen liver tissue samples, isolated cells and fluids using the QIAamp DNA Mini Kit (QIAGEN) according to the manufacturer's instructions. Eluted DNA was analyzed by ddPCR to quantify copy number of AAV vectors with vector-specific primer-probe sets (Supplementary Table 5). Primer-probe sets targeting the intron of human or mouse *GAPDH* were included to measure the amount of genomic DNA in the

sample. The vg to diploid genome ratio was calculated by normalizing the copy number of vgs to the copy number of genomic DNA.

qRT-PCR and ddPCR

Total RNA was isolated from frozen liver tissue samples by homogenization in Trizol reagent (Invitrogen) and column purification with the PureLink RNA Mini Kit (Invitrogen). Before elution, DNase I was used to remove contaminating DNA. Total RNA from frozen cell pellets was isolated using the PureLink RNA Mini Kit with addition of DNase I. cDNA was synthesized with SuperScript IV VILO Master Mix (Invitrogen) according to the manufacturer's instructions. The expression of cell-type-specific markers was analyzed with primers (Supplementary Table 5) and SYBR green reagent (Applied Biosystems) using a ViiA7 real-time PCR system (Applied Biosystems) with QuantStudio Real-Time PCR Software (v1.3). The expression of AAV mRNA was analyzed by ddPCR after mixing cDNA with primer-probe sets (Supplementary Table 5) and ddPCR Supermix for Probes. AAV mRNA expression in each sample was normalized to the expression level of human *GAPDH* mRNA.

For validation of primer-probe sets, the copy number of AAV vector plasmids was measured by ddPCR. An estimated copy number of each AAV vector plasmid was calculated by spectrophotometry and then 1 x 10^4 copies μl^{-1} of plasmid were mixed with 5 ng μl^{-1} of genomic DNA isolated from human hepatocytes at 1:1 ratio.

Read mapping confirmation

All reads were confirmed to be unique and correct for the transgene or barcode of interest using the nucleotide BLAST command line application (NCBI, blastn v2.13.0). IGV[9] (v2.8.13) was used to visualize reads mapped to the custom genomes. Due to the sequence homology of mCardinal and mTagBFP2, individual mapped reads were manually confirmed using the nucleotide BLAST command line application. Briefly, reads for each gene were extracted from output bam files and aligned to either the mCardinal or mTagBFP2 reference sequence, generating an E-value and Bitscore for every read. These scores, read lengths and base mismatches were compared side by side. In all cases, reads mapped to mCardinal were confirmed to have lower E-values, higher Bitscores, longer read lengths and fewer base mismatches than the same reads mapped to mTagBFP2. Similarly, reads mapped to mTagBFP2 were confirmed to have lower E-values, higher Bitscores, longer read lengths and fewer base mismatches than the same reads mapped to mCardinal.

Single-cell clustering analysis

Cells captured by scRNA-seq (NL 2 = 6,959 cells, NL 3 = 10,701 cells, NL 4 = 8,024 cells, SL 3 = 5,471 cells, SL 4 = 11,611 cells) were clustered and analyzed using Seurat[10] (v4.1.1) in R (v4.1.2). First, digital gene expression matrices from each sample array were merged to create a single Seurat object for each human liver. Preliminary quality control involved filtering cells with fewer than 200 genes or 200 transcripts and a mitochondrial percentage greater than 30. Then, distribution histograms were examined for number of genes, number of transcripts and mitochondrial percentage. Based on average distribution, upper and lower thresholds were adjusted to eliminate outliers (potential doublets, empty droplets, dying cells, etc.) in each sample.

Preliminary Seurat objects were processed using a standard pipeline. The "LogNormalize" method was used to normalize gene expression for each cell, that is, read counts for each cell were divided by the total counts for that cell, multiplied by a scale factor of 10,000 and natural-log transformed using log1p. For NL 3, NL 4, SL 3 and SL 4, the top 1,000 most variably expressed genes were calculated for each object before identifying integration features and anchors using SelectIntegrationFeatures and FindIntegrationAnchors, respectively. After integration using the IntegrateData function, linear scaling was applied to all genes and principal component analysis was performed using the integrated data. For NL 2, linear scaling was applied directly after calculation of the top 1,000 variably expressed genes, which were then used for principal component analysis. Fluorescent protein and barcode genes associated with AAV vectors were removed from variable features and integration features prior to downstream processing. Principal components used for UMAP dimension reduction were determined using elbow plots of principal component versus standard deviation. The variation due to batch effect and cell cycle was assessed and its influence on principal components was minimal. For UMAP-based clustering, the clustree (v0.5.0) package[11] was used to inform choice of biologically relevant clusters. Statistically significant cluster-defining genes were identified using a combination of FindMarkers and FindAllMarkers functions. *P* values were calculated using Wilcoxon rank-sum test and adjusted with Bonferroni's correction.

Cell type annotation

To annotate each cell type, cell identity was first broadly defined using the SingleR (v1.8.1) package[12]. From there, the most highly expressed and differentially regulated genes for each cluster were analyzed and cross-referenced with existing literature[13-19]. Differentially expressed genes used to define each cell type for each human liver are detailed in Supplementary Figs. 5 and 8.

NPC subclustering analysis

Clusters defined as macrophages or endothelial cells in NL 3, NL 4, SL 3 and SL 4 were subset and integrated as described above in “Single-cell clustering analysis”. Macrophages with high expression of plasma cell, biliary, endothelial or hepatocyte markers were removed prior to final clustering. Endothelial cells with high expression of T cell, hepatocyte or hepatic stellate cell markers were removed before final clustering. Fluorescent protein and barcode genes were removed from integration features and the list of differentially expressed genes used for principal component analysis. Macrophage[13,15,18] and endothelial cell[20-23] subtypes were defined using differential marker gene expression cross-referenced with existing literature. Differentially expressed genes used to define each cell subtype are detailed in Extended Data Fig. 9.

AAV vector co-regulated gene analysis

Cell IDs corresponding to cells transduced with a single vector (AAV8 = 577 cells, AAV5 = 101 cells, AAV6 = 98 cells, AAV-LK03 = 2,619 cells, AAV-NP59 = 354 cells) were extracted and annotated in the object metadata (“Only”). Then, cell IDs corresponding to cells which received no vectors were extracted and annotated in the object metadata (“None”). FindMarkers was performed comparing “Only” cells versus “None” cells for each vector group per human liver. *P* values were calculated using Wilcoxon rank-sum test and adjusted with Benjamini-Hochberg procedure. Genes with a log fold-change greater than 0.1 were considered. Genes were categorized into biologic and functional groups using GeneCards[24], g:Profiler[25] and cross-referencing with existing literature.

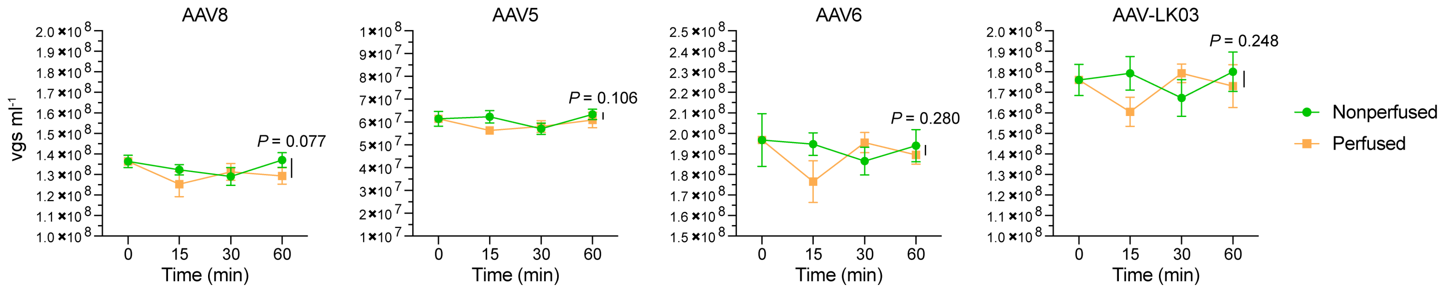

Supplementary Fig. 1 | Analysis of attachment of AAV vectors to silicone tubing. Quantification of AAV vgs in medium that was either nonperfused or perfused through silicone tubing. Values are presented as mean ± s.d. (n = 3, technical replicates). Statistical significance was determined using two-way ANOVA with Tukey's post hoc test.

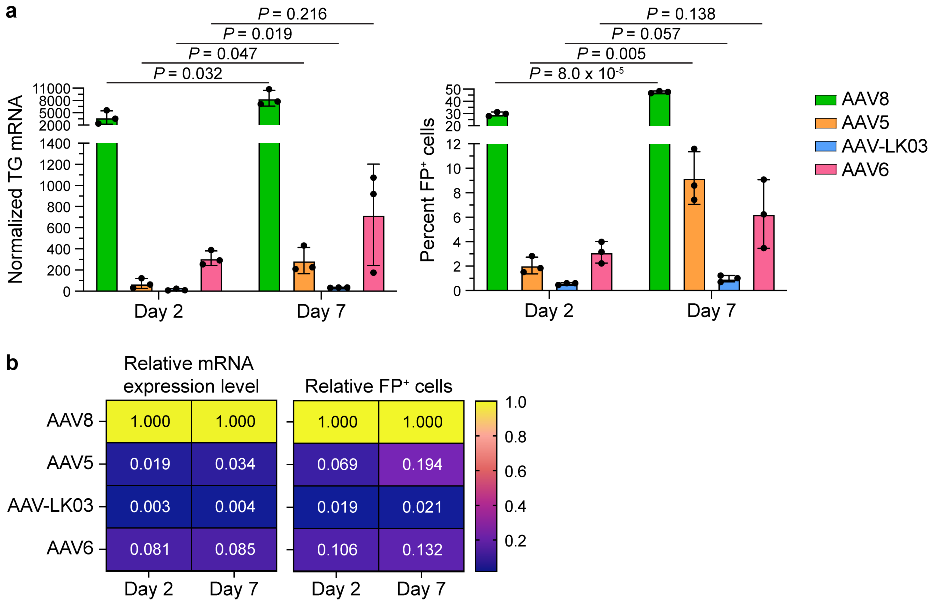

Supplementary Fig. 2 | Rapid functional transduction by an scAAV-CMV vector. a, Quantification of AAV transgene (TG) mRNA expression by ddPCR (left) and AAV fluorescent protein (FP) expression by flow cytometry (right) in hepatocytes isolated from mice 48 h and 7 days after co-injection of four scAAV vectors at a dose of 2 x 10^{11} vgs each. Values are presented as mean ± s.d. (*n* = 3 mice per group). **b**, Heat map showing relative levels of AAV transgene mRNA expression (left) and AAV fluorescent protein-expressing hepatocytes (right) normalized to the levels of AAV8 on day 2 and day 7. The levels from three mice were averaged for each AAV capsid.

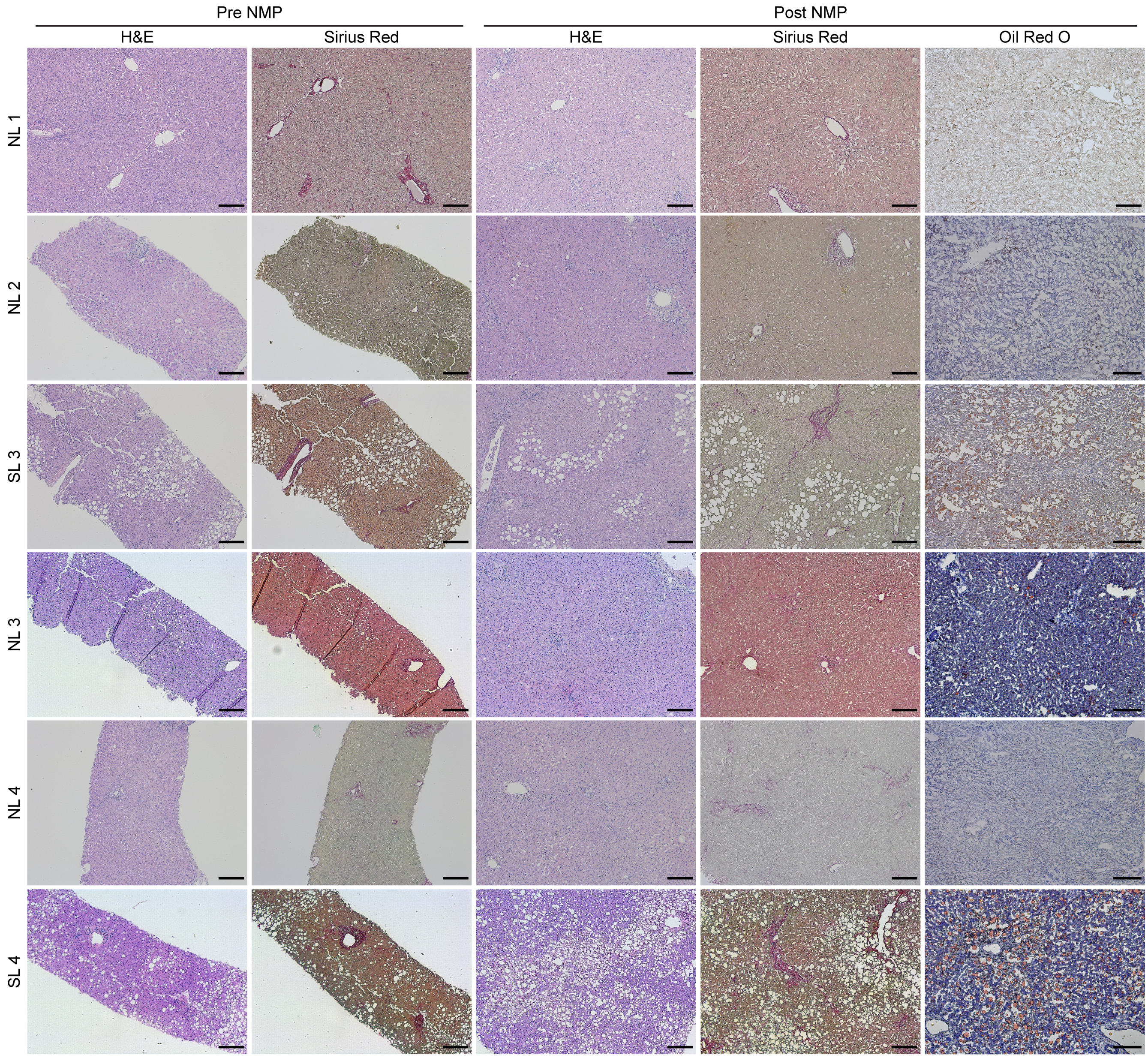

Supplementary Fig. 3 | Identification of normal and steatotic human livers. Stainings of liver tissue samples before and after NMP; scale bars, 200 μm.

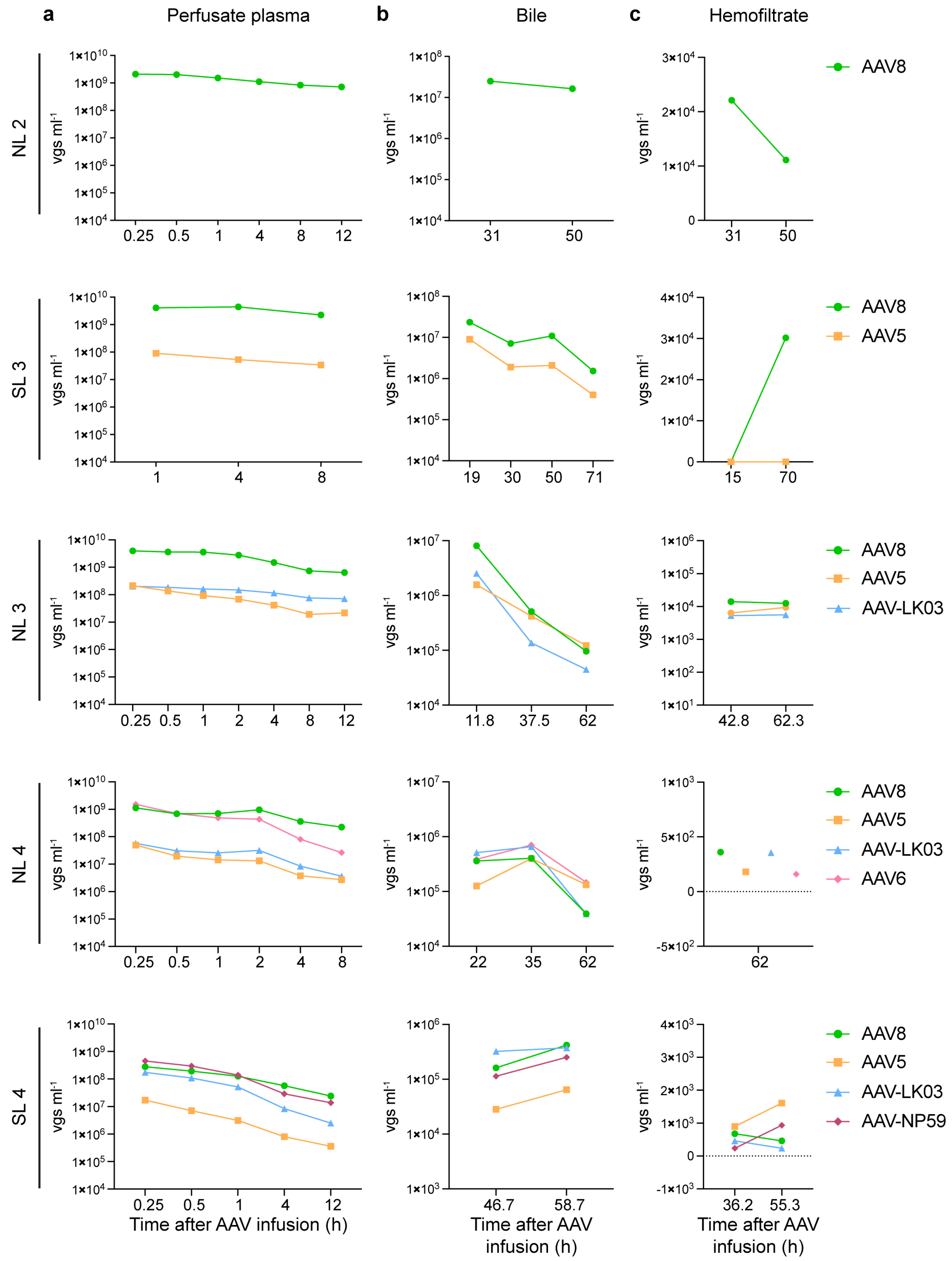

Supplementary Fig. 4 | AAV vgs in NMP fluids. a-c, AAV vgs measured in perfusate plasma (**a**), bile (**b**) and hemofiltrate (**c**) from NL 2, SL 3, NL 3, NL 4 and SL 4. Values are presented as mean (*n* = 2 except *n* = 4 for SL 4 in (**c**), technical replicates).

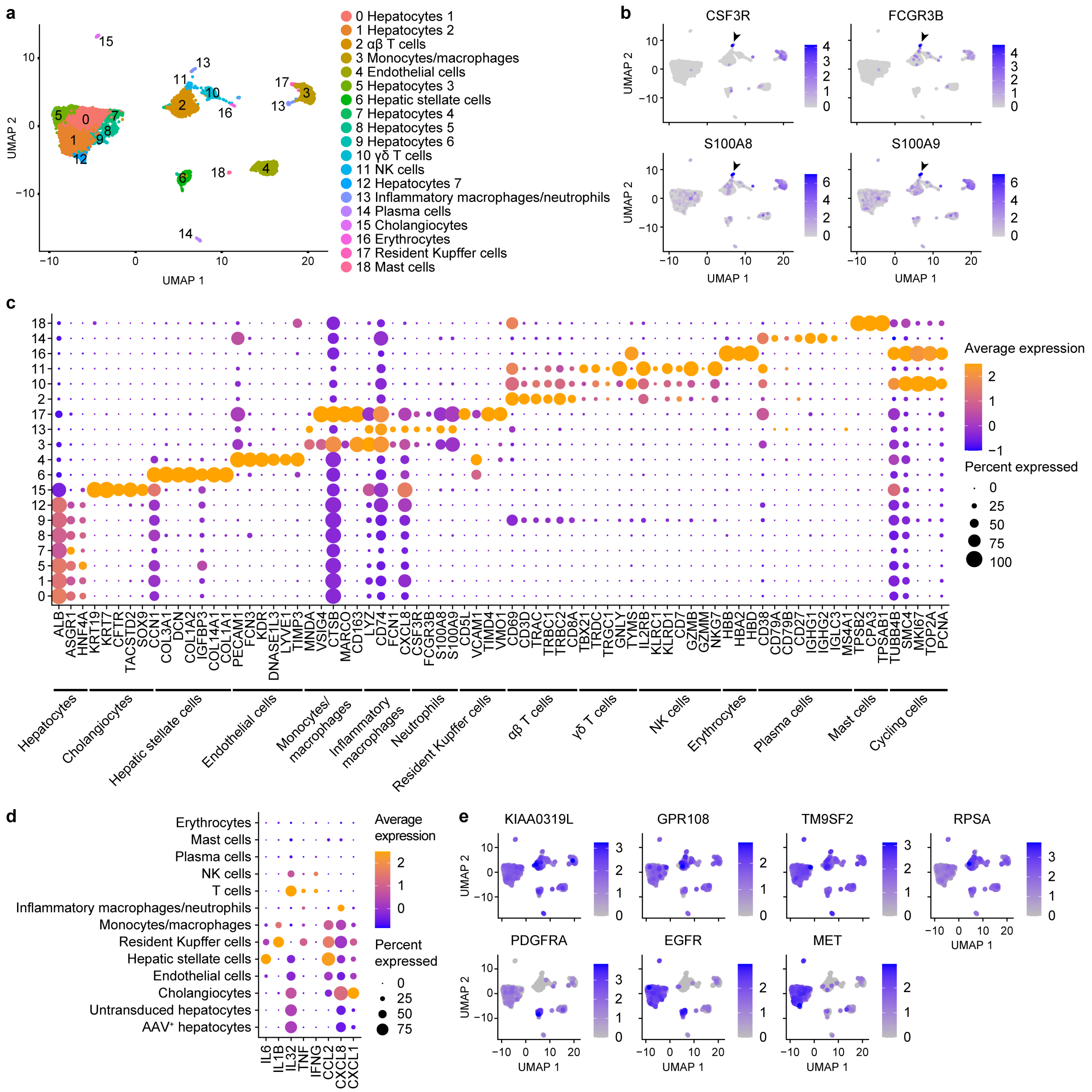

Supplementary Fig. 5 | Cell type identification in NL 2. a, UMAP of 6,959 liver cells identified by cell type. **b**, Neutrophil-specific markers indicating a subset of high-expressing cells in cluster 13 (black arrowheads). **c**, Cell-type-specific markers used to identify diverse cell populations. **d**, Inflammatory cytokine and chemokine expression in all cell populations. **e**, AAV entry factor expression in all cell types.

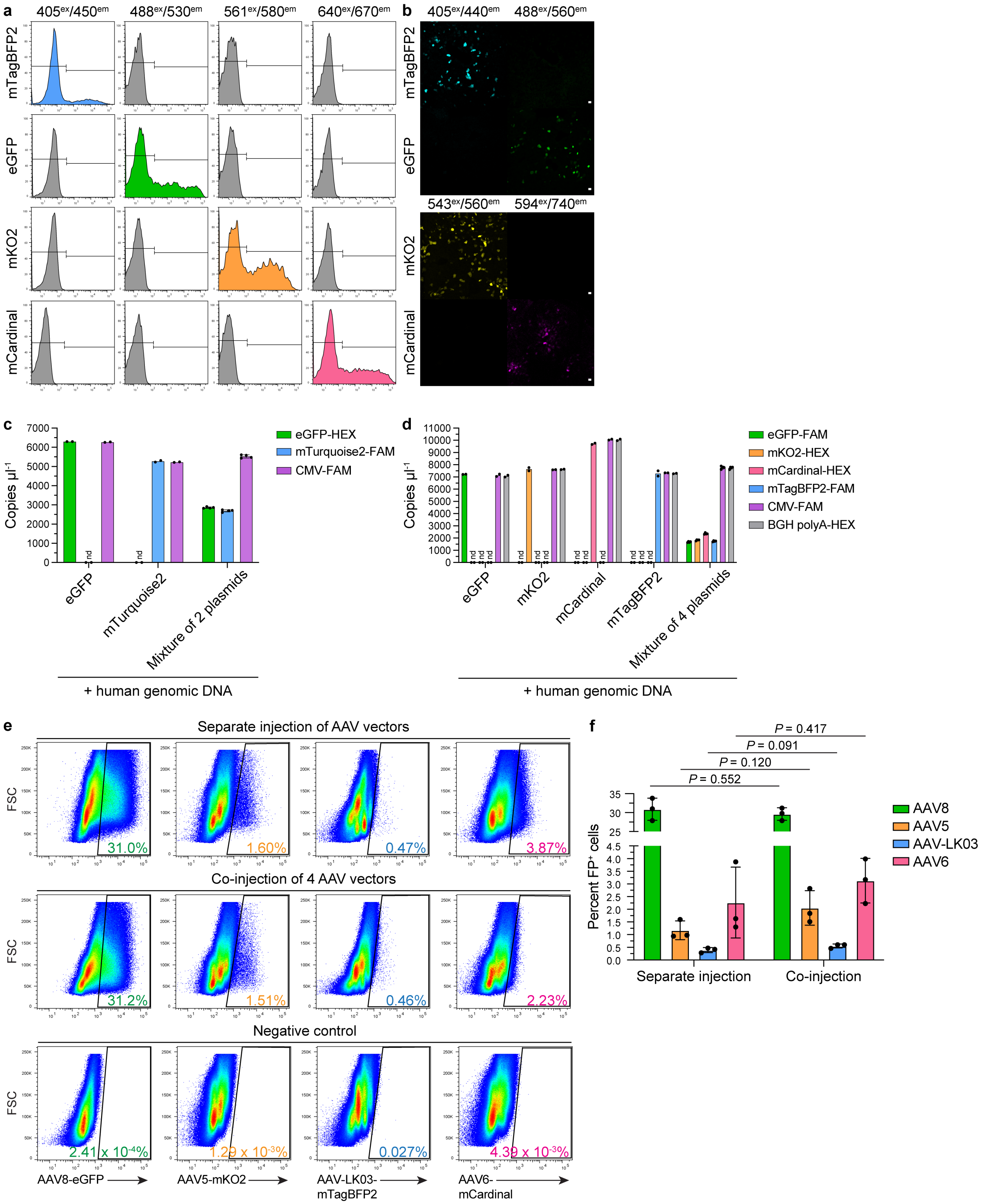

Supplementary Fig. 6 | Distinction of four vectors containing fluorescent protein-encoding transgenes by flow cytometry, fluorescence microscopy and ddPCR. a, Flow cytometry and spectral overlap analysis of HEK293 cells 2 days after transfection with AAV vector plasmids encoding four fluorescent proteins. **b**, Confocal microscopy of mouse livers 2 days after hydrodynamic tail vein injection of AAV vector plasmids encoding four

fluorescent proteins; ex, excitation; em, emission; scale bars, 25 μm. **c**,**d**, Copy number of AAV vector plasmids determined by ddPCR using primer-probe sets targeting transgenes, CMV promoter or BGH polyA sequences. Samples were spiked with human genomic DNA. AAV vector plasmids were used to produce AAV vectors infused into SL 3 (**c**) and NL 3, NL 4 and SL 4 (**d**); nd, not detected. Bar graphs represent mean ± s.d. (*n* = 2 for single plasmids, *n* = 4 for mixture of plasmids; technical replicates). **e**,**f**, Flow cytometry (**e**) with quantification (**f**) of hepatocytes isolated from mice 48 h after separate injection or co-injection of four scAAV vectors expressing fluorescent protein-encoding transgenes from the CMV promoter at a dose of 2 x 10^{11} vgs each. Values are presented as mean ± s.d. (*n* = 3 mice each for separate-injection group and co-injection group, biological replicates). Means were compared using two-tailed unpaired *t*-tests.

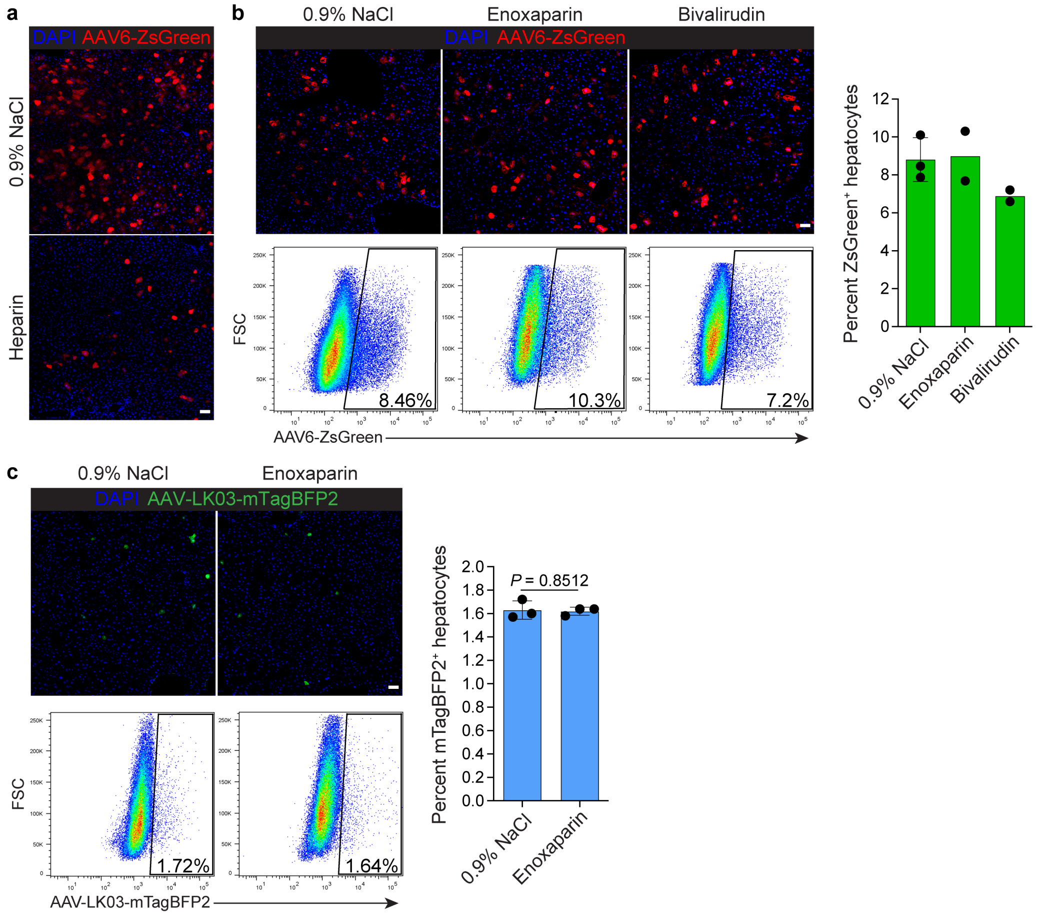

Supplementary Fig. 7 | Unaltered hepatocyte transduction by AAV6 and AAV-LK03 capsids in mice treated with low-molecular-weight heparin. a, Immunofluorescence for ZsGreen in liver tissue samples from mice 7 days after intravenous injection of 0.9% NaCl solution or 5,000 U kg^{-1} of heparin followed by 4 x 10^{11} vgs of AAV6-ZsGreen 30 min later; scale bar, 25 µm. **b**, Immunofluorescence for ZsGreen in liver tissue samples and flow cytometry with quantification of hepatocytes isolated from mice 2 days after intravenous injection of 0.9% NaCl solution, 2 mg kg^{-1} of enoxaparin (low-molecular-weight heparin) or 1 mg kg^{-1} of bivalirudin (synthetic polypeptide) followed by 4 x 10^{11} vgs of AAV6-ZsGreen 30 min later; scale bar, 25 µm. Values are presented as mean ± s.d. (*n* = 3 mice for 0.9% NaCl solution, *n* = 2 mice for enoxaparin and bivalirudin; biological replicates). **c**, Immunofluorescence for mTagBFP2 in liver tissue samples and flow cytometry with quantification of hepatocytes isolated from mice 7 days after intravenous injection of 0.9% NaCl solution or 2 mg kg^{-1} of enoxaparin followed by 2 x 10^{11} vgs of AAV-LK03-mTagBFP2 30 min later; scale bar, 25 µm. Values are presented as mean ± s.d. (*n* = 3 mice per group, biological replicates). Means were compared using two-tailed unpaired *t*-tests.

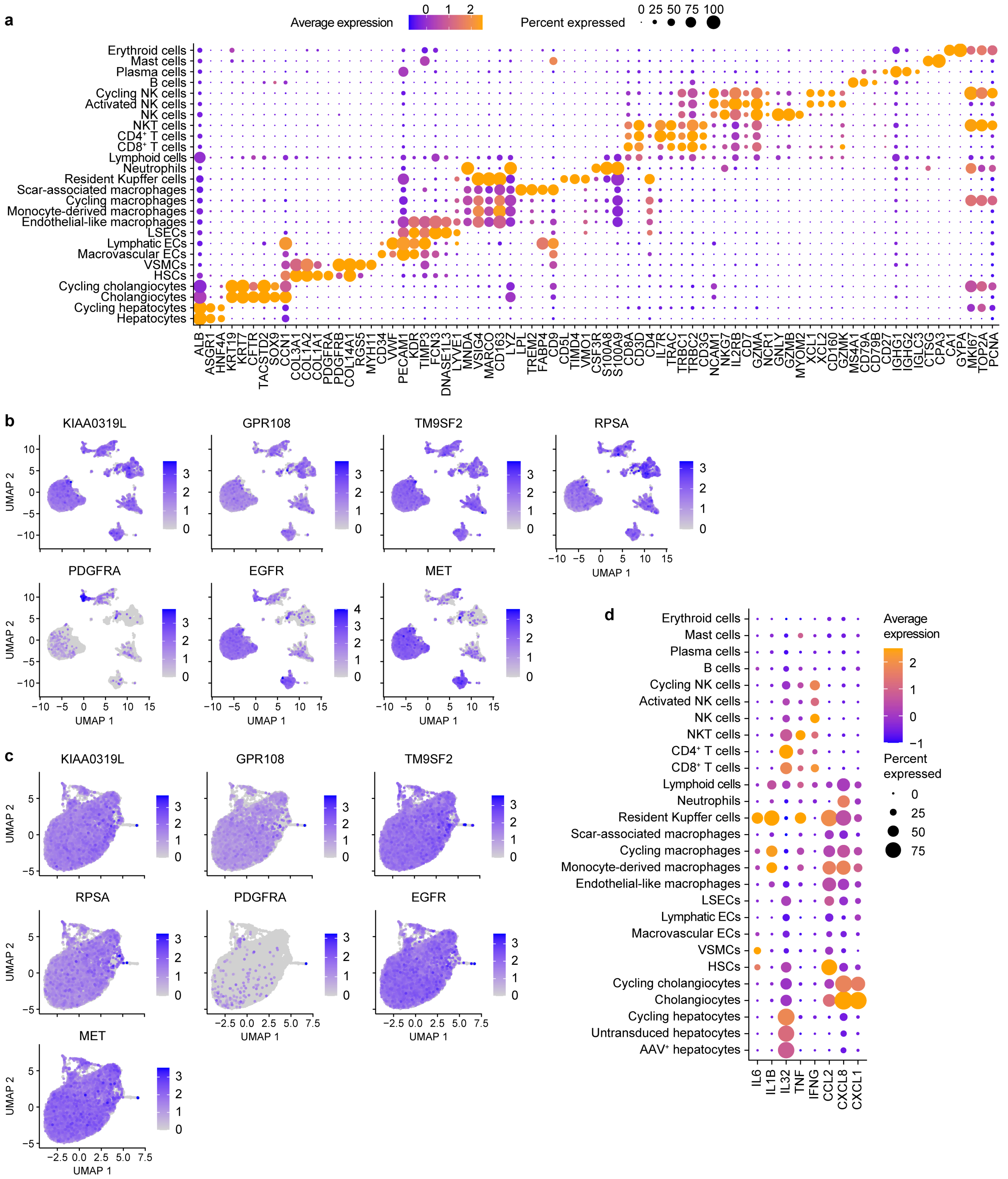

Supplementary Fig. 8 | Cell type identification across four human livers. a, Cell-type-specific markers used to identify diverse cell populations. **b**, AAV entry factor expression in all cell types. **c**, AAV entry factor expression in hepatocytes. **d**, Inflammatory cytokine and chemokine expression in all cell types.

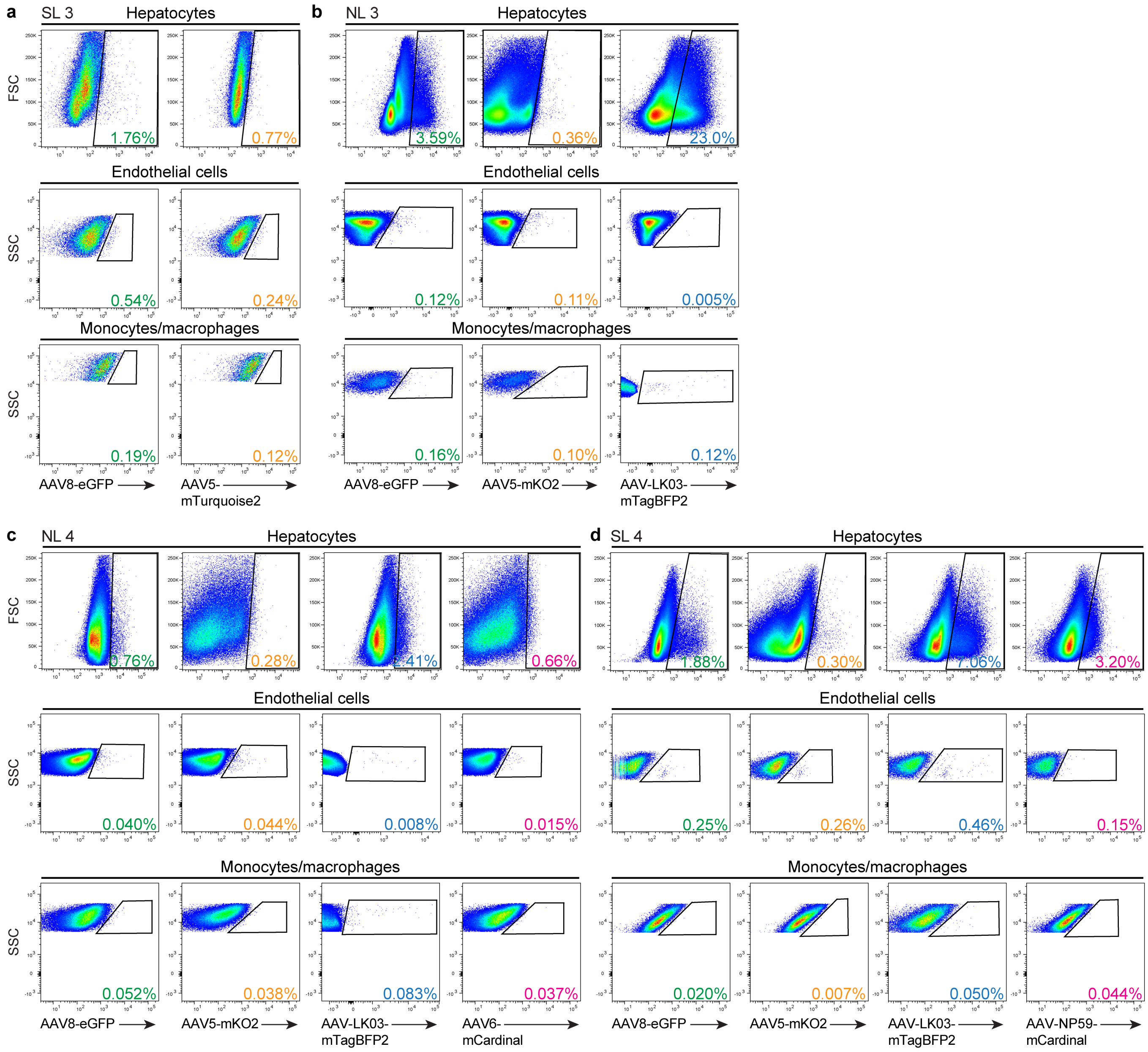

Supplementary Fig. 9 | Functional transduction of various cell types by AAV vectors. a-d, Flow cytometry of AAV-transduced cells from SL 3 (**a**), NL 3 (**b**), NL 4 (**c**) and SL 4 (**d**).

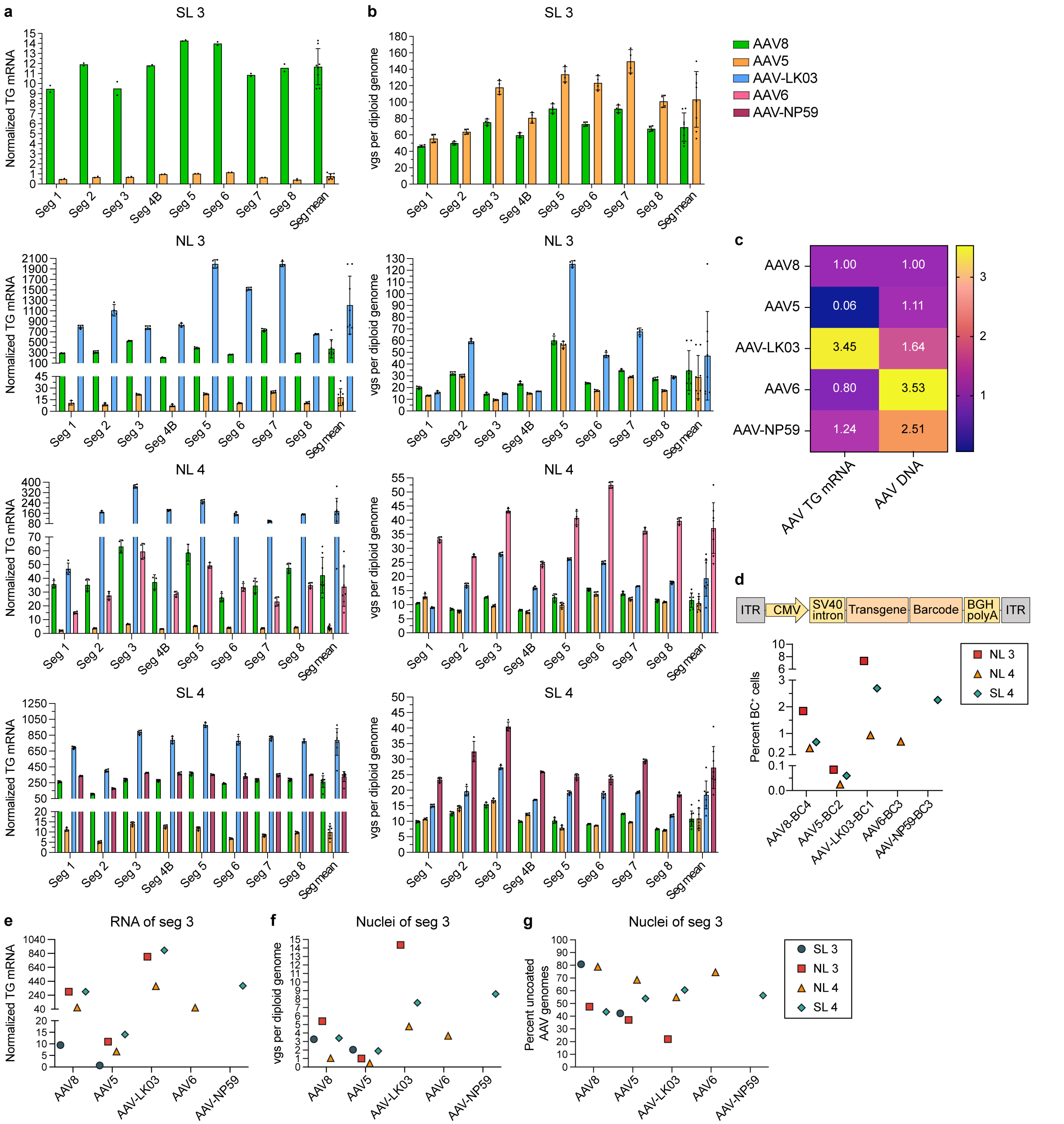

Supplementary Fig. 10 | AAV mRNA and DNA analysis in human livers. a, **b**, Quantification of AAV transgene mRNA expression (**a**) and AAV vgs per diploid genome (**b**) by ddPCR in tissue samples; Seg, segment; Seg mean, mean expression values of all eight segments. Values are presented as mean ± s.d. (n = 4 except n = 2 for SL 3 in (**a**), technical replicates). **c**, Heat map showing the relative levels of AAV vector mRNA and DNA in tissue samples for five different AAV capsids normalized to the levels of AAV8. The levels from four livers were averaged for each capsid. **d**, Schematic of AAV vector and quantification of hepatocytes expressing

barcode (BC) mRNA by scRNA-seq. **e-g**, Quantification of AAV transgene mRNA expression (**e**) in tissue samples and quantification of AAV vgs per diploid genome (**f**) and percentage of uncoated vgs among whole nuclear vgs per diploid genome (**g**) in nuclei from segment 3.

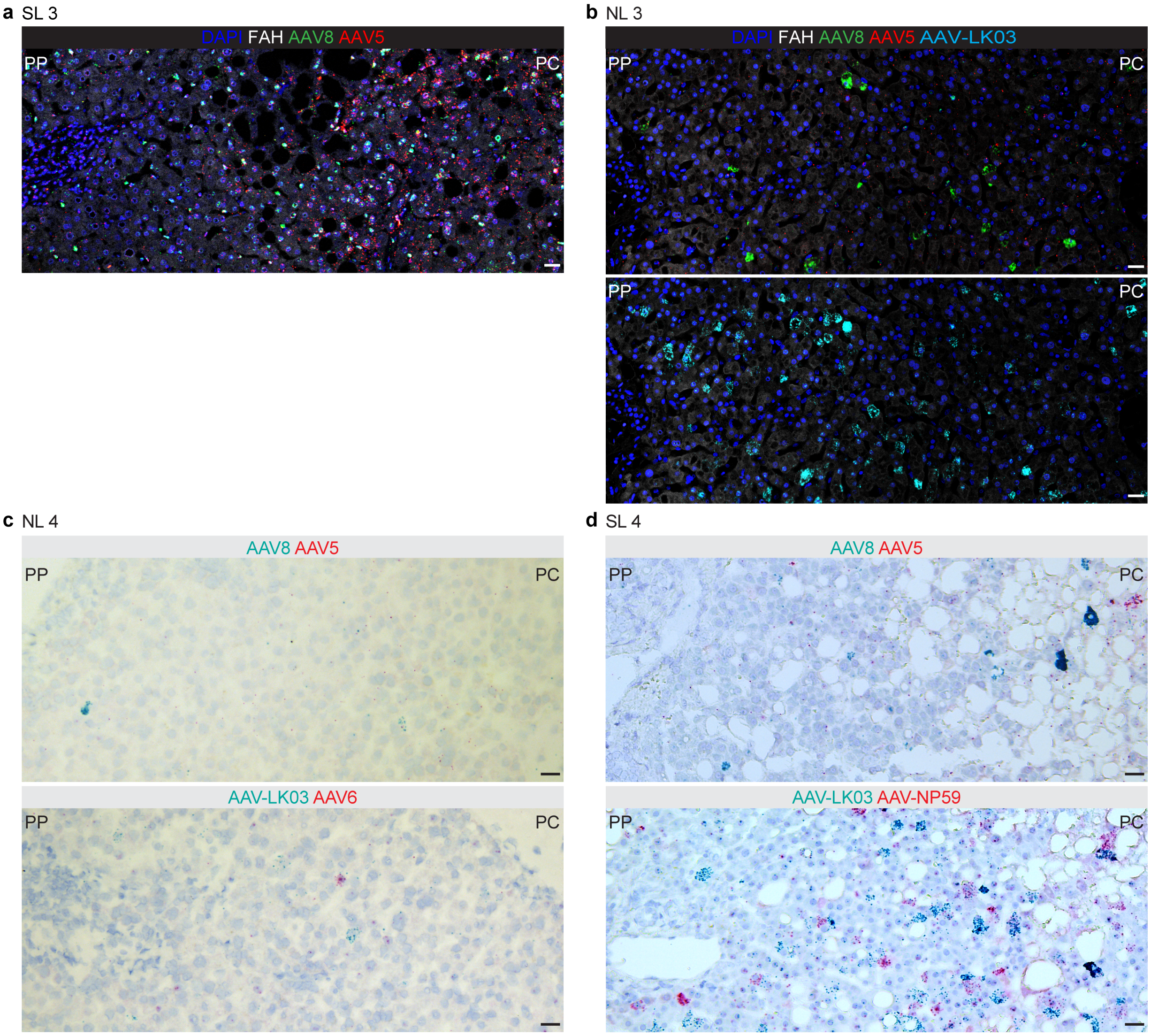

Supplementary Fig. 11 | ISH of AAV mRNA and DNA in human livers. a-d, ISH of AAV vector mRNA and DNA using antisense probes in tissue samples from SL 3 (**a**), NL 3 (**b**), NL 4 (**c**) and SL 4 (**d**); scale bars, 25 μm.

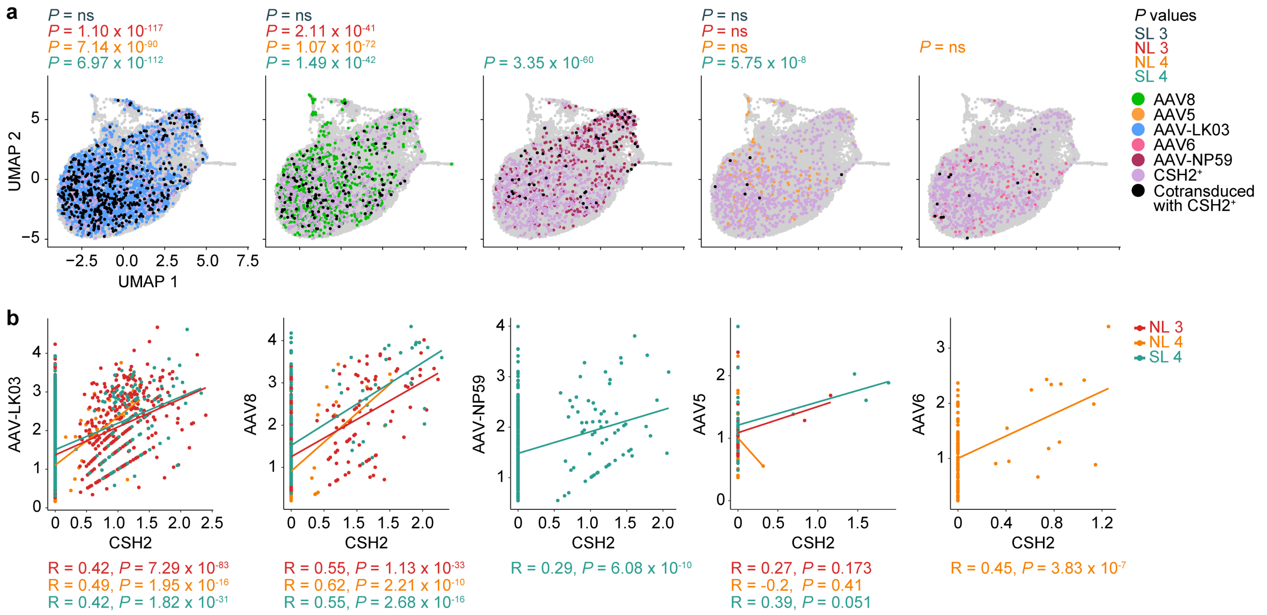

Supplementary Fig. 12 | Co-regulated genes in AAV-transduced hepatocytes. a, UMAPs of 22,146 hepatocytes showing co-expression of AAV vectors with *CSH2*. **b**, Gene expression scatterplot showing significant positive correlation between AAV-LK03, AAV8, AAV-NP59 and AAV6 and *CSH2* expression. No significant positive correlation was observed between AAV5 and *CSH2* expression. SL 3 is omitted from scatterplots as no significant relationship with *CSH2* was identified in (**a**). *P* values calculated using Wilcoxon rank-sum test and adjusted with Benjamini-Hochberg procedure relating to Supplementary Data 1 (**a**) or Pearson correlation coefficients with two-sided *P* values (**b**) are shown; ns, not significant.

Supplementary Table 1 | Donor and liver characteristics.

	**Steatotic liver 1**	**Steatotic liver 2**	**Normal liver 1**	**Normal liver 2**
Age (years)	57	50	37	63
Sex	Male	Female	Female	Female
Body/liver* weight (kg)	118/2.2	71.9/1.5	119/2.2	85.6/1.7
Body mass index	39.0	28.4	43.7	28.9
History of heavy alcohol use	No	Yes	No	No
History of diabetes mellitus	Yes (>10 years)	No	No	No
Peak serum alanine transaminase ($U\ l^{-1}$)	79	54	91	55
Peak serum total bilirubin ($mg\ dl^{-1}$)	1.6	0.9	0.6	1.9
Donor type	DCD	DCD	DCD	DCD
Histological steatosis/fibrosis assessment** (pre NMP)	Not performed	Steatosis: 1 (~20%)<br>Fibrosis: 1c<br>Ballooning: 0<br>Lobular inflammation: 1	Steatosis: 0<br>Fibrosis: 0<br>Ballooning: 0<br>Lobular inflammation: 0	Steatosis: 0<br>Fibrosis: 0<br>Ballooning: 0<br>Lobular inflammation: 1
Histological steatosis/fibrosis assessment** (post NMP)	Steatosis: 2<br>Fibrosis: 2<br>Ballooning:1<br>Lobular inflammation: 1	Steatosis: 1<br>Fibrosis: 1c<br>Ballooning: 0<br>Lobular inflammation: 1-2	Steatosis: 0<br>Fibrosis: 0<br>Ballooning: 0<br>Lobular inflammation: 0	Steatosis: 0<br>Fibrosis: 1c<br>Ballooning: 1<br>Lobular inflammation: 1

	**Steatotic liver 3**	**Normal liver 3**	**Normal liver 4**	**Steatotic liver 4**
Age (years)	31	29	39	33
Sex	Male	Male	Male	Female
Body/liver* weight (kg)	133.1/2.4	61.7/1.3	67.6/1.4	53.5/1.2
Body mass index	42.7	22.7	23.9	20
History of heavy alcohol use	Yes	Yes	No	Yes
History of diabetes mellitus	No	No	No	Yes
Peak serum alanine transaminase ($U\ l^{-1}$)	641	37	49	58
Peak serum total bilirubin ($mg\ dl^{-1}$)	1.5	0.7	2.7	1.3
Donor type	DCD	DCD	DBD	DBD
Histological steatosis/fibrosis assessment** (pre NMP)	Steatosis: 2<br>Fibrosis: 1c<br>Ballooning: 2<br>Lobular inflammation: 1<br>Cholestasis	Steatosis: 1 (10%)<br>Fibrosis: 0<br>Ballooning: 0<br>Lobular inflammation:1-2	Steatosis: 1 (~12%, macrovesicular)<br>Fibrosis: 0<br>Ballooning: 0<br>Lobular inflammation: 1	Steatosis: 3 (75%, macrovesicular)<br>Fibrosis: 1b/1c<br>Ballooning: 0<br>Lobular inflammation: 1<br>Cholestasis
Histological steatosis/fibrosis assessment** (post NMP)	Steatosis: 2<br>Fibrosis: 1c<br>Ballooning: 0<br>Lobular inflammation: 2<br>Cholestasis	Steatosis: 0 (<3%)<br>Fibrosis: 0<br>Ballooning: 0<br>Lobular inflammation: 2	Steatosis: 0 (~3%)<br>Fibrosis: 1c<br>Ballooning: 0<br>Lobular inflammation: 1	Steatosis: 3 (75%)<br>Fibrosis: 1b/1c<br>Ballooning: 1<br>Lobular inflammation: 1<br>Cholestasis

Abbreviations: DCD, donation after circulatory death; DBD, donation after brain death.
*Estimated liver weight[26].
**Scale of histology scoring: steatosis 0-3; fibrosis 0-4; ballooning 0-2; lobular inflammation 0-3[27].

Supplementary Table 2 | AAV vector characteristics.

	**Normal liver 1**	**Normal liver 2**	**Steatotic liver 3**	**Normal liver 3**	**Normal liver 4**	**Steatotic liver 4**
AAV capsids	AAV8	AAV8	AAV8; AAV5	AAV8; AAV5; AAV-LK03	AAV8; AAV6; AAV5; AAV-LK03	AAV8; AAV5; AAV-LK03; AAV-NP59
Infused dose (vgs)	5.7 x 10^{12}	1.6 x 10^{13}	3.4 x 10^{13} each	5.5 x 10^{12} each	6.7 x 10^{11} each	3.0 x 10^{12} each
Expression cassette*	scAAV; CMV-SV40 intron-TG-SV40 polyA	scAAV; CMV-SV40 intron-TG-BGH polyA	scAAV; CMV-TG-BC-SV40 polyA	scAAV; CMV-SV40 intron-TG-BC-BGH polyA	scAAV; CMV-SV40 intron-TG-BC-BGH polyA	scAAV; CMV-SV40 intron-TG-BC-BGH polyA
TG, size (base pairs) of expression cassette*	eGFP, 1685	eYFP, 1764	AAV8: eGFP, 1891<br>AAV5: mTurquoise2, 1888	AAV8: eGFP, 1800<br>AAV5: mKO2, 1737<br>AAV-LK03: mTagBFP2, 1785	AAV8: eGFP, 1800<br>AAV5: mKO2, 1737<br>AAV-LK03: mTagBFP2, 1785<br>AAV6: mCardinal-Flag, 1971	AAV8: eGFP, 1800<br>AAV5: mKO2, 1737<br>AAV-LK03: mTagBFP2, 1785<br>AAV-NP59: mCardinal-Flag, 1971
Virus production	University of North Carolina Vector Core	Andelyn Biosciences	Nationwide Children's Hospital Viral Vector Core	Signagen	Signagen	Signagen
Total NMP time (h)	59	62	84	69	68	68
NMP time (h) after vector infusion	48	50	73	62	62	60
Vector GC per diploid genome in hepatocytes	0.24	2.85	AAV8: 3.28<br>AAV5: 2.43	AAV8: 37.46<br>AAV5: 14.95<br>AAV-LK03: 70.89	AAV8: 2.85<br>AAV5: 2.18<br>AAV-LK03: 8.04<br>AAV6: 10.87	AAV8: 3.58<br>AAV5: 3.86<br>AAV-LK03: 8.24<br>AAV-NP59: 11.09
Percent of TG-expressing hepatocytes in scRNA-seq	Not performed	1.37	AAV8: 4.31<br>AAV5: 2.12	AAV8: 5.92<br>AAV5: 0.39<br>AAV-LK03: 27.65	AAV8: 1.88<br>AAV5: 0.45<br>AAV-LK03: 5.55<br>AAV6: 2.55	AAV8: 2.3<br>AAV5: 0.31<br>AAV-LK03: 8.82<br>AAV-NP59: 5.57
Percent of FP-expressing hepatocytes in flow cytometry	0.11	0.82	AAV8: 1.76<br>AAV5: 0.77	AAV8: 3.59<br>AAV5: 0.36<br>AAV-LK03: 23	AAV8: 0.76<br>AAV5: 0.28<br>AAV-LK03: 2.41<br>AAV6: 0.66	AAV8: 1.88<br>AAV5: 0.3<br>AAV-LK03: 7.06<br>AAV-NP59: 3.2

Abbreviations: GC, genome copy; TG, transgene; BC, barcode; FP, fluorescent protein.
*The expression cassette consists of regulatory elements and transgene sequences flanked by 5' and 3' ITRs.

Supplementary Table 3 | Antibodies used for flow cytometry and immunofluorescence.

Anti-human antibody for flow cytometry	Clone	Source	Catalog #	Dilution
CD45	HI30	BD	557748	1:100
CD14	MφP9	BD	562691	1:100
CD31	WM59	Biolegend	303116	1:100
EPCAM	EBA-1	BD	743544	1:100
PDGFRB	28D4	BD	743039	1:100
CD90	5E10	BD	561970	1:200
CD26	BA5b	Biolegend	302718	1:100
β2-microglobulin	2M2	Biolegend	316317	1:100

Anti-mouse antibody for flow cytometry	Clone	Source	Catalog #	Dilution
CD45	30-F11	Biolegend	103113	1:100
CD31	390	Biolegend	102417	1:100
EPCAM	G8.8	Biolegend	118215	1:100
PDGFRB	APB5	Invitrogen	25-1402-82	1:100

Primary antibody for immunofluorescence	Host	Source	Catalog #	Dilution
Vimentin	Rabbit	Abcam	ab92547	1:100
PDGFRA	Goat	R&D Systems	AF-307-SP	1:100
ACTA2	Rabbit	Abcam	ab5694	1:200
FAH	Rabbit	Markus Grompe, OHSU[6]		1:250
CD31	Rabbit	Proteintech	11265-1-AP	1:100
CD68	Rabbit	Invitrogen	PA5-83940	1:100
GFP	Goat	Abcam	ab6673	1:250
CXCL8	Mouse	ThermoFisher Scientific	M801	1:50
HNF4A	Goat	Santa Cruz Biotechnology	sc-6556	1:250
TROP2	Rabbit	Abcam	ab214488	1:100
TagFP	Alpaca	NanoTag Biotechnologies	N0501-At488-S	1:50
ZsGreen	Rabbit	Takara	632474	1:100

Secondary antibody for immunofluorescence	Source	Catalog #	Dilution
Donkey anti-Rabbit IgG, Alexa Fluor 488	Invitrogen	A-21206	1:500
Donkey anti-Rabbit IgG, Alexa Fluor 647	Invitrogen	A-31573	1:500
Donkey anti-Goat IgG, Alexa Fluor 488	Invitrogen	A-11055	1:500
Donkey anti-Goat IgG, Alexa Fluor 555	Invitrogen	A-21432	1:500
Donkey anti-Mouse IgG, Alexa Fluor 555	Invitrogen	A-31570	1:500

Supplementary Table 4 | Probes used for ISH of AAV vector DNA and mRNA.

Probe for in situ hybridization	Tissue	Source	Catalog #
eGFP-O4-antisense	NL 1 and NL 2	ACDBio	538851 RNAscope probe
eGFP-sense		ACDBio	409971 RNAscope probe
eGFP-BC4-sense/antisense	NL 3, NL 4 and SL 4	ACDBio	Custom RNAscope probe
mKO2-BC2-sense/antisense		ACDBio	Custom RNAscope probe
mTagBFP2-BC1-sense/antisense		ACDBio	Custom RNAscope probe
eGFP-BC4-sense/antisense		ACDBio	Custom BaseScope probe
mKO2-BC2-sense/antisense		ACDBio	Custom BaseScope probe
mTagBFP2-BC1-sense/antisense		ACDBio	Custom BaseScope probe
mCardinal-Flag-BC3-sense/antisense		ACDBio	Custom BaseScope probe
eGFP-HA-BC2-sense/antisense	SL 3	ACDBio	Custom RNAscope probe
mTurquoise2-Flag-BC1-sense/antisense		ACDBio	Custom RNAscope probe
BaseScope Duplex Control Probe Pack-Hs	NL 4 and SL 4	ACDBio	322981
RNAscope 4-plex Positive Control Probe-Hs	NL 1, NL 2, NL 3, NL 4, SL 3 and SL 4	ACDBio	321801
RNAscope 4-plex Negative Control Probe	NL 1, NL 2, NL 3, NL 4, SL 3 and SL 4	ACDBio	321831

Opal dye	Source	Catalog #	Dilution
Opal 570 Reagent Pack	Akoya Biosciences	FP1488001KT	1:1,000
Opal 620 Reagent Pack	Akoya Biosciences	FP1495001KT	1:1,000

Supplementary Table 5 | Sequences for qPCR primers and ddPCR/digital PCR primer-probe sets.

qPCR primers	Direction	Sequence
GAPDH	Forward	GCTCATTTCCTGGTATGACAACG
	Reverse	AGGGGTCTACATGGCAACTG
CD26	Forward	AGTGGCACGGCAACACATT
	Reverse	AGAGCTTCTATCCCGATGACTT
ALB	Forward	TTTATGCCCCGGAACTCCTTT
	Reverse	AGTCTCTGTTTGGCAGACGAA
OTC	Forward	CTCACCCTCAGCTGGATAGG
	Reverse	CCCTTTGGAGTAGCTGCTTG
ASGR1	Forward	ATGAAGTCGCTAGAGTCCCAG
	Reverse	CAGGTCAGACACGAACTGCTT
A1AT	Forward	GATCAACGATTACGTGGAGAAGG
	Reverse	CCTAAACGCTTCATCATAGGCA
FAH	Forward	GGAGAATGCGTTGATGCCAAA
	Reverse	CATATACGGGAGGCTTAGAGTCA
CD14	Forward	GACCTAAAGATAACCGGCACC
	Reverse	GCAATGCTCAGTACCTTGAGG
CD163	Forward	GACGCATTTGGATGGATCATGT
	Reverse	CCCACCGTCCTTGGAATTTGA
CD68	Forward	CTTCTCTCATTCCCCTATGGACA
	Reverse	GAAGGACACATTGTACTCCACC
MARCO	Forward	CAGCGGGTAGACAACTTCACT
	Reverse	TTGCTCCATCTCGTCCCATAG
CD31	Forward	AGCAGCATCGTGGTCAACATAAC
	Reverse	GCAGGACAGGTTCAGTCTTTCA
VWF	Forward	CGTGGTCCTGAAGCAGACATA
	Reverse	TTGCTGCTGGTGAGGTCATT
CLEC4G	Forward	CCCGTGATATGCCTCCACTT
	Reverse	ACAGCTTCCAGTTTGGTGGA
CELC4M	Forward	ACTTCATGTCTAACTCCCAGCG
	Reverse	ATTCCGCACAGTCTTCATTCC
EPCAM	Forward	ATAACCTGCTCTGAGCGAGTG
	Reverse	TGCAGTCCGCAAACTTTTACTA
KRT19	Forward	TGAGTGACATGCGAAGCCAAT
	Reverse	CTCCCGGTTCAATTCTTCAGTC
CFTR	Forward	CCTATGACCCGGATAACAAGGA
	Reverse	GAACACGGCTTGACAGCTTTA
PDGFRB	Forward	AGACACGGGAGAATACTTTTGC
	Reverse	AGTTCCTCGGCATCATTAGGG
RGS5	Forward	GACATGGCCCAGAAAAGAATCC
	Reverse	CACAAAGCGAGGCAGAGAATC
NGFR	Forward	GAGTCAGGTTTGGGGTTCGT
	Reverse	GCTGATTCTAGGGGCCAGTG

VIM	Forward	GACGCCATCAACACCGAGTT
	Reverse	CTTTGTCGTTGGTTAGCTGGT
DES	Forward	GAGACCATCGCGGCTAAGAAC
	Reverse	GTGTAGGACTGGATCTGGTGT
LRAT	Forward	CAACTTCACGCTCTTTAGTTCGG
	Reverse	GGCAACACGGTTGTCTCCT

**ddPCR/digital PCR primer-probe sets**	**Direction**	**Sequence**	**Experiment**
eGFP	Forward	CTGGACGGCGACGTAAAC	NL 1, NL 2
	Reverse	CGGTGGTGCAGATGAACTT	
	Probe	ATGCCACCTACGGCAAGCTGA	
eGFP-BC4	Forward	CCTGCTGGAGTTCGTGACC	NL 3, NL 4, SL 4
	Reverse	GTGCTTCTCTCACATAGTGGTACAT	
	Probe	AGCTGTATAAGATCGCACGTGGCA	
mKO2-BC2	Forward	CCATCGCCTCGTCAGGAAA	
	Reverse	GAGGTACATAGGACATGAACGAGAG	
	Probe	CCGAAGGCAACATTACTGAGCAGGT	
mCardinal-BC3	Forward	AGCCGACTACAAAGACCATGAC	
	Reverse	CTGTGTGAAGTAGCGTCGATGAA	
	Probe	ACAAGGATGACGATGACAAGGGCG	
mTagBFP2-BC1	Forward	CCAGATACTGCGACCTCCCTA	
	Reverse	GGTGAGTTCTTATCCGTTGCAGT	
	Probe	TCTGTATAGTGGTAGGTGACGGCGA	
eGFP-BC2	Forward	TTATGCAGGAAGCGGATGAG	SL 3
	Reverse	ACGGTCTAATAAAGCGGAGTAAA	
	Probe	TGTGGTACTACGTTTGGCGGCTAA	
mTurquoise2-BC1	Forward	CTACAAGGACGACGATGACAAG	
	Reverse	AACTTTCGCACTCCGTACTATC	
	Probe	TTTCACCAAACGTCTCGCATGCAC	
Human GAPDH_gDNA	Forward	CCACACACATGCACTTACCT	Quantification of human genome
	Reverse	CTAGTCCCAGGGCTTTGATTT	
	Probe	AGCTAGGAAGGACAGGCAACTTGG	
Human GAPDH_mRNA	Forward	CCACTCCTCCACCTTTGAC	Normalization of mRNA level
	Reverse	ACCCTGTTGCTGTAGCCA	
	Probe	TTGCCCTCAACGACCACTTTGTC	
Mouse GAPDH_gDNA	Forward	ACTCCTCATGGGTCTGTAGT	Quantification of mouse genome
	Reverse	CTTTGTTGTGGTACGTGCATAG	
	Probe	TTGCACCATAGGTGTGGAGAACCT	
CMV promoter	Forward	TGCCCAGTACATGACCTTATG	Vector titration and primer-probe validation
	Reverse	GAAATCCCCGTGAGTCAAACC	
	Probe	AGTCATCGCTATTACCATGG	
BGH polyA	Forward	GCCAGCCATCTGTTGT	Primer-probe validation
	Reverse	GGAGTGGCACCTTCCA	
	Probe	TCCCCCGTGCCTTCCTTGACC	

Supplementary Data 1 | Co-regulated genes in AAV-transduced hepatocytes. Differentially expressed genes in hepatocytes transduced with AAV vectors (tabs 1-5) and similarities in differentially expressed genes between AAV vectors (tab 6, gray boxes indicate enrichment); BC, barcode. *P* values less than 0.05 calculated using Wilcoxon rank-sum test and adjusted with Benjamini-Hochberg procedure are shown.

Supplementary References

1 Proffitt, S., Curnow, E., Brown, C., Bashir, S. & Cardigan, R. Comparison of automated and manual methods for washing red blood cells. *Transfusion* **58**, 2208-2216 (2018).

2 Reinhart, W. H. *et al.* Washing stored red blood cells in an albumin solution improves their morphologic and hemorheologic properties. *Transfusion* **55**, 1872-1881 (2015).

3 Meliani, A. *et al.* Determination of anti-adeno-associated virus vector neutralizing antibody titer with an in vitro reporter system. *Hum Gene Ther Methods* **26**, 45-53 (2015).

4 Pillay, S. *et al.* An essential receptor for adeno-associated virus infection. *Nature* **530**, 108-112 (2016).

5 Lock, M., Alvira, M. R., Chen, S.-J. & Wilson, J. M. Absolute determination of single-stranded and self-complementary adeno-associated viral vector genome titers by droplet digital PCR. *Hum Gene Ther Methods* **25**, 115-125 (2014).

6 Wilson, E. M. *et al.* Extensive double humanization of both liver and hematopoiesis in FRGN mice. *Stem Cell Res* **13**, 404-412 (2014).

7 Li, B., Dorrell, C., Canady, P. S. & Wakefield, L. Identification and Isolation of Clonogenic Cholangiocyte in Mouse. *Methods Mol. Biol.* **1905**, 19-27 (2019).

8 Groeger, M. *et al.* Modeling and therapeutic targeting of inflammation-induced hepatic insulin resistance using human iPSC-derived hepatocytes and macrophages. *Nat. Commun.* **14**, 3902 (2023).

9 Robinson, J. T. *et al.* Integrative genomics viewer. *Nat. Biotechnol.* **29**, 24-26 (2011).

10 Satija, R., Farrell, J. A., Gennert, D., Schier, A. F. & Regev, A. Spatial reconstruction of single-cell gene expression data. *Nat. Biotechnol.* **33**, 495-502 (2015).

11 Zappia, L. & Oshlack, A. Clustering trees: a visualization for evaluating clusterings at multiple resolutions. *GigaScience* **7** (2018).

12 Aran, D. *et al.* Reference-based analysis of lung single-cell sequencing reveals a transitional profibrotic macrophage. *Nat. Immunol.* **20**, 163-172 (2019).

13 Ramachandran, P. *et al.* Resolving the fibrotic niche of human liver cirrhosis at single-cell level. *Nature* **575**, 512-518 (2019).

14 Aizarani, N. *et al.* A human liver cell atlas reveals heterogeneity and epithelial progenitors. *Nature* **572**, 199-204 (2019).

15 MacParland, S. A. *et al.* Single cell RNA sequencing of human liver reveals distinct intrahepatic macrophage populations. *Nat. Commun.* **9**, 4383 (2018).

16 Payen, V. L. *et al.* Single-cell RNA sequencing of human liver reveals hepatic stellate cell heterogeneity. *JHEP Rep* **3**, 100278 (2021).

17 Brancale, J. & Vilarinho, S. A Single Cell Gene Expression Atlas of 28 Human Livers. *J. Hepatol.* **75** (2021).

18 Wang, Z.-Y. *et al.* Single-cell and bulk transcriptomics of the liver reveals potential targets of NASH with fibrosis. *Sci. Rep.* **11**, 19396 (2021).

19 Doherty, D. G. & O'Farrelly, C. Innate and adaptive lymphoid cells in the human liver. *Immunol. Rev.* **174**, 5-20 (2000).

20 den Braanker, H., van Stigt, A. C., Kok, M. R., Lubberts, E. & Bisoendial, R. J. Single-Cell RNA Sequencing Reveals Heterogeneity and Functional Diversity of Lymphatic Endothelial Cells. *Int. J. Mol. Sci.* **22** (2021).

21 Lutter, S., Xie, S., Tatin, F. & Makinen, T. Smooth muscle–endothelial cell communication activates Reelin signaling and regulates lymphatic vessel formation. *J. Cell Biol.* **197**, 837-849 (2012).

22 Fujimoto, N. *et al.* Single-cell mapping reveals new markers and functions of lymphatic endothelial cells in lymph nodes. *PLoS Biol.* **18**, e3000704 (2020).

23 Schupp, J. C. *et al.* Integrated Single-Cell Atlas of Endothelial Cells of the Human Lung. *Circulation* **144**, 286-302 (2021).

24 Stelzer, G. *et al.* The GeneCards Suite: From Gene Data Mining to Disease Genome Sequence Analyses. *Curr Protoc Bioinformatics* **54**, 1 30 31-31 30 33 (2016).

25 Raudvere, U. *et al.* g:Profiler: a web server for functional enrichment analysis and conversions of gene lists (2019 update). *Nucleic Acids Res.* **47**, W191-W198 (2019).

26 Ichihara, N. *et al.* Achieving clinically optimal balance between accuracy and simplicity of a formula for manual use: Development of a simple formula for estimating liver graft weight with donor anthropometrics. *PLoS One* **18**, e0280569 (2023).

27 Kleiner, D. E. *et al.* Design and validation of a histological scoring system for nonalcoholic fatty liver disease. *Hepatology* **41**, 1313-1321 (2005).
