# Supplementary material for: AAV capsid prioritization in normal and steatotic human livers maintained by machine perfusion
Source: Nat Biotechnol. 2025 Jan 29;43(12):1966–78. doi: 10.1038/s41587-024-02523-6 (PMC12304247; doi:10.1038/s41587-024-02523-6)
Supplement: Supplementary file 2 — Reporting Summary [file 41587_2024_2523_MOESM2_ESM.pdf]

# nature portfolio

Corresponding author(s): Holger Willenbring

Last updated by author(s): Nov 19, 2024

# Reporting Summary

Nature Portfolio wishes to improve the reproducibility of the work that we publish. This form provides structure for consistency and transparency in reporting. For further information on Nature Portfolio policies, see our Editorial Policies and the Editorial Policy Checklist.

## Statistics

For all statistical analyses, confirm that the following items are present in the figure legend, table legend, main text, or Methods section.

| n/a | Confirmed | |
|---|---|---|
| ☐ | ☒ | The exact sample size (*n*) for each experimental group/condition, given as a discrete number and unit of measurement |
| ☐ | ☒ | A statement on whether measurements were taken from distinct samples or whether the same sample was measured repeatedly |
| ☐ | ☒ | The statistical test(s) used AND whether they are one- or two-sided<br>*Only common tests should be described solely by name; describe more complex techniques in the Methods section.* |
| ☐ | ☒ | A description of all covariates tested |
| ☐ | ☒ | A description of any assumptions or corrections, such as tests of normality and adjustment for multiple comparisons |
| ☐ | ☒ | A full description of the statistical parameters including central tendency (e.g. means) or other basic estimates (e.g. regression coefficient) AND variation (e.g. standard deviation) or associated estimates of uncertainty (e.g. confidence intervals) |
| ☐ | ☒ | For null hypothesis testing, the test statistic (e.g. *F*, *t*, *r*) with confidence intervals, effect sizes, degrees of freedom and *P* value noted<br>*Give P values as exact values whenever suitable.* |
| ☒ | ☐ | For Bayesian analysis, information on the choice of priors and Markov chain Monte Carlo settings |
| ☒ | ☐ | For hierarchical and complex designs, identification of the appropriate level for tests and full reporting of outcomes |
| ☐ | ☒ | Estimates of effect sizes (e.g. Cohen's *d*, Pearson's *r*), indicating how they were calculated |

*Our web collection on statistics for biologists contains articles on many of the points above.*

## Software and code

Policy information about availability of computer code

| | |
|---|---|
| Data collection | FACSDiva (v9.4) for collecting flow cytometry data.<br>Leica Application Suite X (v4.6.1.27508) for collecting microscopy images.<br>QuantStudio Real-Time PCR Software (v1.3) for collecting qRT-PCR data. |
| Data analysis | All software and software algorithms are specified where applicable in the Methods section:<br>blastn (v2.13.0), clustree (v0.5.0), Fiji (ImageJ 1.53q), FlowJo (v10.9.0), ggplot2 (v3.4.4), ggpubr (v0.6.0), GraphPad Prism (v9.4.1), IGV (v2.8.13), Nebulosa (v1.4.0), QIAcuity Software Suite (v2.1.7), QuantaSoft (v1.7), R (v4.1.2), Seurat (v4.1.1), SingleR (v1.8.1), UCell (v2.5.1). |

For manuscripts utilizing custom algorithms or software that are central to the research but not yet described in published literature, software must be made available to editors and reviewers. We strongly encourage code deposition in a community repository (e.g. GitHub). See the Nature Portfolio guidelines for submitting code & software for further information.

## Data

Policy information about availability of data

All manuscripts must include a data availability statement. This statement should provide the following information, where applicable:

- Accession codes, unique identifiers, or web links for publicly available datasets
- A description of any restrictions on data availability
- For clinical datasets or third party data, please ensure that the statement adheres to our policy

Raw scRNAseq FASTQ files and processed DGE matrices generated in this study are deposited in the Gene Expression Omnibus (GEO) under the accession number GSE228000. hg38 reference genome (Homo sapiens GRCh38.p13) was used for alignment. Source data are provided for Extended Data Fig. 6c. Additional data are available from the corresponding author upon reasonable request.

## Human research participants

Policy information about studies involving human research participants and Sex and Gender in Research.

| | |
|---|---|
| Reporting on sex and gender | The human liver donors included 4 females and 4 males. Sex was not considered in this study because it was not a relevant factor and the number of subgroups was insufficient to analyze differences between sexes. |
| Population characteristics | Human livers declined for transplantation were used in this study. The characteristics of donors and livers are provided in Supplementary Table 1. |
| Recruitment | Human livers declined for transplantation were obtained through regional Organ Procurement Organizations (OPOs) from donation after brain death and donation after circulatory death. Informed consent was obtained by the OPOs. |
| Ethics oversight | The UCSF Institutional Review Board Committee for Human Research Protection Program determined that this study was exempt from Institutional Review Board review as it does not meet the FDA's definition of a clinical investigation involving human subjects (21 CFR 50.3 and 21 CFR 812.3). |

Note that full information on the approval of the study protocol must also be provided in the manuscript.

# Field-specific reporting

Please select the one below that is the best fit for your research. If you are not sure, read the appropriate sections before making your selection.

☒ Life sciences ☐ Behavioural & social sciences ☐ Ecological, evolutionary & environmental sciences

For a reference copy of the document with all sections, see nature.com/documents/nr-reporting-summary-flat.pdf

# Life sciences study design

All studies must disclose on these points even when the disclosure is negative.

| | |
|---|---|
| Sample size | For human liver NMP experiments, sample size was determined based on previously published studies utilizing the NMP system (PMIDs 33602855, 30821045, 27027254) and organ availability. For mouse validation experiments, 2-3 mice were included in each group. |
| Data exclusions | No data were excluded. |
| Replication | All attempts at replication were successful. Technical and biological replicates were used in all experiments as noted in text, figure legends and methods. Representative microscope images were taken from at least two independent experiments. |
| Randomization | In human liver NMP experiments, normal and steatotic livers were chosen intentionally to identify differences in AAV vector tropism between these conditions. For all animal studies, mice were randomly assigned to control or experimental groups. |
| Blinding | Researchers were not blinded during treatments and analysis. The condition of human livers was visually discernible and the tracking of mice in experimental groups was necessary. |

# Reporting for specific materials, systems and methods

We require information from authors about some types of materials, experimental systems and methods used in many studies. Here, indicate whether each material, system or method listed is relevant to your study. If you are not sure if a list item applies to your research, read the appropriate section before selecting a response.

## Materials & experimental systems

| n/a | Involved in the study | |
|---|---|---|
| ☐ | ☒ | Antibodies |
| ☐ | ☒ | Eukaryotic cell lines |
| ☒ | ☐ | Palaeontology and archaeology |
| ☐ | ☒ | Animals and other organisms |
| ☒ | ☐ | Clinical data |
| ☒ | ☐ | Dual use research of concern |

## Methods

| n/a | Involved in the study | |
|---|---|---|
| ☒ | ☐ | ChIP-seq |
| ☐ | ☒ | Flow cytometry |
| ☒ | ☐ | MRI-based neuroimaging |

# Antibodies

**Antibodies used**

Detailed information on antibodies is shown in Supplementary Table 3.

**Validation**

Anti-human antibodies for flow cytometry were validated by qRT-PCR analysis of cell type-specific markers in target cell populations isolated by FACS (Extended Data Fig. 6).

Validation data for all antibodies used in this study, including anti-human and anti-mouse antibodies, are available on the suppliers' websites.
Anti-human CD45 https://www.bdbiosciences.com/en-us/products/reagents/flow-cytometry-reagents/research-reagents/single-color-antibodies-ruo/pe-cy-7-mouse-anti-human-cd45.557748
Anti-human CD14 https://www.bdbiosciences.com/en-us/products/reagents/flow-cytometry-reagents/research-reagents/single-color-antibodies-ruo/pe-mouse-anti-human-cd14.562691
Anti-human CD31 https://www.biolegend.com/en-us/products/apc-anti-human-cd31-antibody-6123
Anti-human EPCAM https://www.bdbiosciences.com/en-us/products/reagents/flow-cytometry-reagents/research-reagents/single-color-antibodies-ruo/bv711-mouse-anti-human-cd326.743544
Anti-human PDGFRB https://www.bdbiosciences.com/en-us/products/reagents/flow-cytometry-reagents/research-reagents/single-color-antibodies-ruo/buv395-mouse-anti-human-cd140b.743039
Anti-human CD90 https://www.bdbiosciences.com/en-us/products/reagents/flow-cytometry-reagents/research-reagents/single-color-antibodies-ruo/pe-mouse-anti-human-cd90.561970
Anti-human CD26 https://www.biolegend.com/en-us/products/biotin-anti-human-cd26-antibody-12862
Anti-human β2-microglobulin https://www.biolegend.com/en-us/products/pe-cyanine7-anti-human-beta2-microglobulin-antibody-13839
Anti-mouse CD45 https://www.biolegend.com/en-us/products/pe-cyanine7-anti-mouse-cd45-antibody-1903
Anti-mouse CD31 https://www.biolegend.com/en-us/products/pe-cyanine7-anti-mouse-cd31-antibody-3942
Anti-mouse EPCAM https://www.biolegend.com/en-us/products/pe-cyanine7-anti-mouse-cd326-ep-cam-antibody-5303
Anti-mouse PDGFRB https://www.thermofisher.com/antibody/product/CD140b-PDGFRB-Antibody-clone-APB5-Monoclonal/25-1402-82
Anti-Vimentin https://www.bdbiosciences.com/en-us/products/reagents/flow-cytometry-reagents/research-reagents/single-color-antibodies-ruo/buv395-mouse-anti-mouse-h-2kb.742864
Anti-PDGFRA https://www.rndsystems.com/products/human-pdgf-ralpha-antibody_af-307-na
Anti-ACTA2 https://www.abcam.com/en-us/products/primary-antibodies/alpha-smooth-muscle-actin-antibody-ab5694
Anti-CD31 https://www.ptglab.com/products/PECAM1-Antibody-11265-1-AP.htm?srsltid=AfmBOopalXuqy8AeDCl4nWJ8jGDpV5jj89KC_lyKsR8VKZbubOndqOFa
Anti-CD68 https://www.thermofisher.com/antibody/product/CD68-Antibody-Polyclonal/PA5-83940
Anti-GFP https://www.abcam.com/en-us/products/primary-antibodies/gfp-antibody-ab6673
Anti-CXCL8 https://www.thermofisher.com/antibody/product/IL-8-CXCL8-Antibody-clone-3IL8-H10-Monoclonal/M801
Anti-HNF4A https://www.scbt.com/p/hnf-4alpha-antibody-c-19?srsltid=AfmBOopceZIy5bguoGkz4HWMq2GMtGueKKItMlwlVQ-fOqphKrtafuVb
Anti-TROP2 https://www.abcam.com/en-us/products/primary-antibodies/trop2-antibody-epr20043-ab214488
Anti-TagFP https://nano-tag.com/product/fluotag-q-anti-tagfp/
Anti-ZsGreen https://www.takarabio.com/products/antibodies-and-elisa/fluorescent-protein-antibodies/green-fluorescent-protein-antibodies?srsltid=AfmBOor-9C7oRhm0g8AactUAT0jrWvWEs8ltSt8m2P4-rYa2Vp4KMzG4

# Eukaryotic cell lines

Policy information about cell lines and Sex and Gender in Research

**Cell line source(s)**

HEK293 and HeLaRC32 cells from ATCC; Huh-7 cells from JCRB Cell Bank; human induced pluripotent stem cells (WTC; GM25256; hPSCreg: UCSFi001-A) from Bruce Conklin at the Gladstone Institute of Data Science and Biotechnology, also available from Coriell Institute for Medical Research.

**Authentication**

Cell lines were authenticated by ATCC, JCRB Cell Bank and Bruce Conklin laboratory, followed by short tandem repeat analysis.

**Mycoplasma contamination**

Tested negative.

**Commonly misidentified lines**
(See ICLAC register)

No commonly misidentified cell lines were used in the study.

# Animals and other research organisms

Policy information about studies involving animals; ARRIVE guidelines recommended for reporting animal research, and Sex and Gender in Research

| | |
|---|---|
| Laboratory animals | Mice were housed in a barrier animal facility under standard conditions (12-h light/12-h dark cycle, 30-70% humidity, 20-26°C temperature). Wild-type mice (9-12-week-old C57BL/6J, Jackson Laboratory) and FRGN mice (24-30-week-old Fah-/-;Rag2-/-;Il2rg-/-;SirpaNOD/NOD) transplanted with human hepatocytes were used to test AAV vectors. |
| Wild animals | None used. |
| Reporting on sex | Wild-type mice (C57BL/6J, Jackson Laboratory): male; FRGN mice: male and female. |
| Field-collected samples | None used. |
| Ethics oversight | Mice were bred and maintained under barrier conditions and all procedures were approved by the Institutional Animal Care and Use Committee at UCSF. FRGN mice highly repopulated with human hepatocytes were bred and maintained at OHSU and all procedures were approved by the Institutional Animal Care and Use Committee at OHSU. |

Note that full information on the approval of the study protocol must also be provided in the manuscript.

# Flow Cytometry

## Plots

Confirm that:

☒ The axis labels state the marker and fluorochrome used (e.g. CD4-FITC).

☒ The axis scales are clearly visible. Include numbers along axes only for bottom left plot of group (a 'group' is an analysis of identical markers).

☒ All plots are contour plots with outliers or pseudocolor plots.

☒ A numerical value for number of cells or percentage (with statistics) is provided.

## Methodology

| | |
|---|---|
| Sample preparation | Isolation of human liver cells: after termination of NMP, the liver was cleared of perfusate by flushing with 10-15 l of ice-cold 0.9% NaCl solution through the portal vein and hepatic artery until the efflux was clear without any traces of blood. One liter of UW solution was then infused to maintain cell viability during sample processing. Fifty- to 70-g wedges of the right and left liver lobes were cut and immediately immersed in ice-cold UW solution. Sections were further flushed by manual perfusion of UW solution through open vessels before cell release. To release hepatocytes, sections were perfused with calcium- and magnesium-free HBSS containing 20 mM HEPES, penicillin-streptomycin (100 U/ml and 100 µg/ml, respectively) and 1 mM EGTA for 15 min at 37 °C. Sections were then perfused with 1 mg/ml collagenase IV in HBSS containing 20 mM HEPES, penicillin-streptomycin and 5 mM CaCl2·H2O for 22 min at 37 °C. The crude cell preparation was suspended in DMEM containing penicillin-streptomycin and 5% fetal bovine serum, filtered through sterile gauze, pelleted at 70g and washed twice with the same suspension medium. Hepatocytes were further purified by centrifugation through 45% Percoll at 200g for 15 min. Hepatocytes isolated in this fashion were used for flow cytometry/ FACS and scRNA-seq. NPCs were retrieved from the hepatocyte supernatant fraction from the first centrifugation at 70g and reserved for flow cytometry. For dedicated isolation of NPCs, a section was perfused for 15 min with HBSS containing 1 mM EGTA, followed by perfusion with 300 ml of 1 mg/ml pronase in DMEM and subsequently with 1.5 mg/ml collagenase IV in HBSS containing 5 mM CaCl2·H2O for 22 min. The crude cell preparation was filtered through sterile gauze and digested further in 200 ml of 0.5 mg/ml pronase for 30 min at 37 °C with constant shaking. The cell suspension was pelleted at 600g, washed twice and subjected to density gradient centrifugation with 9% Accudenz at 1,400g for 17 min. Hepatic stellate cells were retrieved from the top of the 9% Accudenz gradient, and other NPC populations were retrieved from the midportion of the gradient.<br><br>Isolation of mouse liver cells: the right lobe of the mouse liver was resected for immunofluorescence by ligating the vessels with a 5-0 suture and cutting the lobe above the ligation. The remaining liver tissue was dissociated by perfusing collagenase II in HBSS through the inferior vena cava. Cells were collected by centrifugation at 50g for 2 min and pelleted cells were subjected to Percoll density gradient centrifugation to isolate viable hepatocytes. |
| Instrument | FACSAria II (BD). |
| Software | FACSDiva (BD) was used to collect data and FlowJo (BD) was used to analyze data and generate plots. |
| Cell population abundance | In human liver, CD26+ human hepatocytes represented 18.4% of the CD45-CD31-EPCAM-PDGFRB- cell population. Among NPCs, CD14+ monocytes/macrophages represented 3.41%, CD45-CD31+ endothelial cells represented 2.07% and CD45-EPCAM+ cholangiocytes represented 0.43% of the parent cell population. In wildtype mouse liver, CD45-CD31-EPCAM-PDGFRB- hepatocytes represented 95.9%-99.4% of the parent cell population. In FRGN mouse liver, β2-microglobulin+ human hepatocytes represented 58.9%-66.5% of the parent cell population. |
| Gating strategy | For the hepatocyte population, cells were first gated based on the pattern of FSC-A versus SSC-A. Singlets were gated according to the pattern of SSC-A versus SSC-H followed by FSC-A versus FSC-H. After live cell gating, contaminating NPCs |

were excluded by gating on the CD45-CD31-EPCAM-PDGFRB- cell population. For the NPC population, cells were first gated based on the pattern of FSC-A versus SSC-A. Singlets were gated according to the pattern of SSC-A versus SSC-H followed by FSC-A versus FSC-H. After live-cell gating, the CD45+ and CD45- cell populations were gated for further identification of cell types with positive markers.

☒ Tick this box to confirm that a figure exemplifying the gating strategy is provided in the Supplementary Information.
